# Resolving the Mechanism for H_2_O_2_ Decomposition over Zr(IV)-Substituted Lindqvist Tungstate: Evidence of Singlet Oxygen Intermediacy

**DOI:** 10.1021/acscatal.3c02416

**Published:** 2023-07-24

**Authors:** Nataliya V. Maksimchuk, Jordi Puiggalí-Jou, Olga V. Zalomaeva, Kirill P. Larionov, Vasilii Yu. Evtushok, Igor E. Soshnikov, Albert Solé-Daura, Oxana A. Kholdeeva, Josep M. Poblet, Jorge J. Carbó

**Affiliations:** † Boreskov Institute of Catalysis, Pr. Lavrentieva 5, Novosibirsk 630090, Russia; # Departament de Química Física i Inorgànica, Universitat Rovira i Virgili, 43005 Tarragona, Spain

**Keywords:** DFT, hydrogen peroxide decomposition, Lindqvist tungstate, singlet oxygen, zirconium

## Abstract

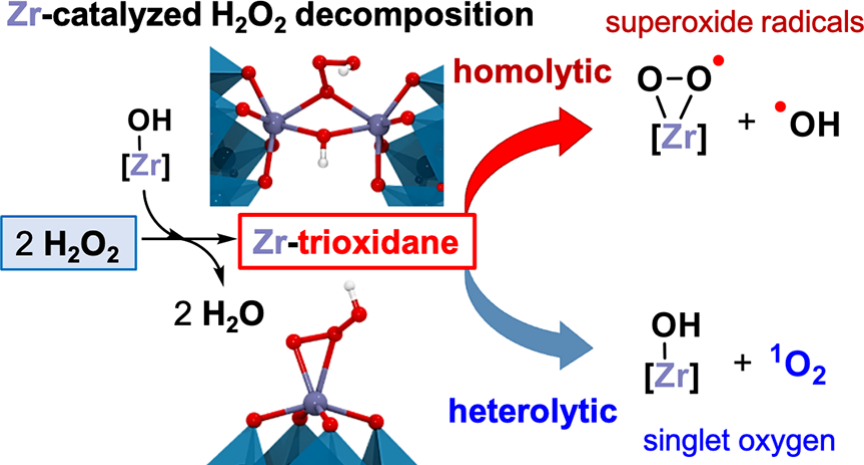

The decomposition of hydrogen peroxide (H_2_O_2_) is the main undesired side reaction in catalytic oxidation processes of industrial interest that make use of H_2_O_2_ as a terminal oxidant, such as the epoxidation of alkenes. However, the mechanism responsible for this reaction is still poorly understood, thus hindering the development of design rules to maximize the efficiency of catalytic oxidations in terms of product selectivity and oxidant utilization efficiency. Here, we thoroughly investigated the H_2_O_2_ decomposition mechanism using a Zr-monosubstituted dimeric Lindqvist tungstate, (Bu_4_N)_6_[{W_5_O_18_Zr(μ-OH)}_2_] (**{ZrW**_**5**_**}**_**2**_), which revealed high activity for this reaction in acetonitrile. The mechanism of the **{ZrW**_**5**_**}**_**2**_-catalyzed H_2_O_2_ degradation in the absence of an organic substrate was investigated using kinetic, spectroscopic, and computational tools. The reaction is first order in the Zr catalyst and shows saturation behavior with increasing H_2_O_2_ concentration. The apparent activation energy is 11.5 kcal·mol^–1^, which is significantly lower than the values previously found for Ti- and Nb-substituted Lindqvist tungstates (14.6 and 16.7 kcal·mol^–1^, respectively). EPR spectroscopic studies indicated the formation of superoxide radicals, while EPR with a specific singlet oxygen trap, 2,2,6,6-tetramethylpiperidone (4-oxo-TEMP), revealed the generation of ^1^O_2_. The interaction of test substrates, α-terpinene and tetramethylethylene, with H_2_O_2_ in the presence of **{ZrW**_**5**_**}**_**2**_ corroborated the formation of products typical of the oxidation processes that engage ^1^O_2_ (endoperoxide ascaridole and 2,3-dimethyl-3-butene-2-hydroperoxide, respectively). While radical scavengers ^*t*^BuOH and *p*-benzoquinone produced no effect on the peroxide product yield, the addition of 4-oxo-TEMP significantly reduced it. After optimization of the reaction conditions, a 90% yield of ascaridole was attained. DFT calculations provided an atomistic description of the H_2_O_2_ decomposition mechanism by Zr-substituted Lindqvist tungstate catalysts. Calculations showed that the reaction proceeds through a Zr-trioxidane [Zr-η^2^-OO(OH)] key intermediate, whose formation is the rate-determining step. The Zr-substituted POM activates heterolytically a first H_2_O_2_ molecule to generate a Zr-peroxo species, which attacks nucleophilically to a second H_2_O_2_, causing its heterolytic O–O cleavage to yield the Zr-trioxidane complex. In agreement with spectroscopic and kinetic studies, the lowest-energy pathway involves dimeric Zr species and an inner-sphere mechanism. Still, we also found monomeric inner- and outer-sphere pathways that are close in energy and could coexist with the dimeric one. The highly reactive Zr-trioxidane intermediate can evolve heterolytically to release singlet oxygen and also decompose homolytically, producing superoxide as the predominant radical species. For H_2_O_2_ decomposition by Ti- and Nb-substituted POMs, we also propose the formation of the TM-trioxidane key intermediate, finding good agreement with the observed trends in apparent activation energies.

## Introduction

The selective oxidation of organic compounds using environmentally friendly oxidants is one of the main goals in oxidation catalysis.^1−3^ A molecule of H_2_O_2_ contains 47% active oxygen, which can potentially be delivered to the organic substrate, leaving water as the only byproduct. Although most hydrogen peroxide is still produced by the anthraquinone process, selective oxidations that employ aqueous hydrogen peroxide are considered to be very attractive from the viewpoint of ecology and economy.^4,5^ However, the efficiency of such oxidation processes is often limited by the contemporaneous nonproductive degradation of H_2_O_2_ into O_2_ and H_2_O. In modern processes for the production of propylene oxide and hydroquinone/pyrocatechol, which are based on the use of the microporous titanium-silicalite-1 (TS-1), the oxidant utilization efficiency is usually 80–90%.^2^ For substrates with relatively low reactivity, the contribution of H_2_O_2_ decomposition becomes significant. Besides being detrimental to the efficiency of the process by itself, the nonproductive degradation of H_2_O_2_ can also deteriorate selectivity, promoting the formation of undesired homolytic oxidation products.

Therefore, the identification of active intermediates and the elucidation of the mechanisms of H_2_O_2_ decomposition would lead to an understanding of the main factors that can be employed to suppress side processes and increase the selectivity of the target oxidation reaction. These may prove to be highly valuable for increasing the oxidant utilization efficiency and product selectivity, with a positive impact on the development of cost-effective, clean, and safe industrial processes.

Several types of active oxygen species are known to form during the homolytic decomposition of hydrogen peroxide, namely, ^•^OH hydroxyl radicals, ^•^O_2_^–^ superoxide radicals (or its protonated form HO_2_^•^), and, more rarely, singlet oxygen ^1^O_2_.^6−11^ Fenton-type H_2_O_2_ degradation in the presence of redox-active transition metals involves electron-transfer steps, leading to the change in the metal oxidation state and formation of ^•^OH and ^•^HO_2_ radicals via the well-known Haber–Weiss mechanism.^12−15^ Other non-Fenton-type homolytic pathways were suggested for catalysts based on metals which are not able at all (Al and other group III metals)^16,17^ or not prone (e.g., Ti^18−20^ and Zr^21−24^) to change their oxidation state. The key feature of these mechanisms is that the metal itself does not participate in the electron-transfer step. Instead, it interacts with hydrogen peroxide to form a metal hydroperoxo species, [M]–OOH (eq 1), and the latter plays a crucial role in the formation of ^•^OH and HO_2_^•^ radicals.
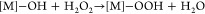
1

Homolytic cleavage of the O–O bond in a hydroperoxo titanium species leading to the generation of TiO^•^ and ^•^OH radicals (eq 2) was suggested to initiate the process of H_2_O_2_ degradation over titanium-silicate catalysts.^25^
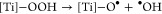
2

However, Don Tilley and co-workers performed DFT calculations on the Ti-, Si-catalyzed decomposition process and revealed that step 2 is energetically unfavorable.^18^ They suggested that the key step is the interaction of [Ti]–OOH with the second H_2_O_2_ molecule, resulting in the formation of TiO^•^ and HO_2_^•^ radicals (eq 3).
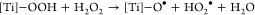
3

The mechanism of H_2_O_2_ unproductive decomposition involving steps 2 and 3 was also supported by Clerici on the basis of characteristic features of H_2_O_2_-based oxidations of hydrocarbons over TS-1.^19,20^ EPR studies confirmed the formation of superoxide radicals both adsorbed and covalently bound to Ti during H_2_O_2_ decomposition over TS-1 and other Ti-silicates,^26,27^ although spectroscopic identification of TiO^•^ still remains a challenge. [Ti]–O_2_^•^ superoxide was suggested to be a side product of hydrogen peroxide decomposition formed through the reaction of lattice [Ti]–OH groups with mildly acidic HO_2_^•^ radical.^19^
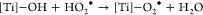
4

Although Zr catalysts have been less studied in their relation to selective oxidation with H_2_O_2_ than Ti-containing ones,^2,3,28−34^ the generation of active oxygen species during H_2_O_2_ decomposition over ZrO_2_ (both amorphous and crystalline) was investigated by several research groups,^21−23,35−38^ first of all, in relation to water radiolysis in nuclear reactors.^36−38^ While early works proposed a mechanism that involves homolytic O–O bond cleavage with the formation of ^•^OH as the first step of H_2_O_2_ decomposition,^21,22,36−38^ more recent studies implicated the simultaneous formation of ^•^O_2_^–^ and ^•^OH radicals.^23^

On the other hand, it is well known that hydrogen peroxide can disproportionate into singlet oxygen (^1^O_2_) and water in the presence of a few non-redox-active catalyst systems, including Na_2_MoO_4_, Na_2_WO_4_, Ca(OH)_2_, La(OH)_3_, and some others.^39−42^ So far, MoO_4_^2–^ is the most efficient catalyst in terms of ^1^O_2_ yield and reaction rate, which makes possible its application on an industrial scale for the production of some valuable fine chemicals.^40,43^ Due to unique nonradical reactivity and specific chemoselectivity, singlet oxygen finds a wide application in organic synthesis,^40,44^ which stimulates the search for new catalysts capable of the “dark” transformation of H_2_O_2_ to ^1^O_2_. Recently, Neumann and co-workers have discovered that a bismuth-substituted polyoxometalate (POM) of the sandwich structure [Zn_2_Bi^III^_2_(ZnW_9_O_34_)_2_]^14–^, combined with H_2_O_2_, realizes ene-type reactivity rather than epoxidation for alkenes and dienes, suggesting the involvement of ^1^O_2_ in the oxidation process.^45^

Our studies on molecular compounds, Zr-substituted polyoxometalates (Zr-POMs), demonstrated significant catalytic activity of Zr(IV) in the H_2_O_2_-based oxidation of organic compounds.^30−32,46^ Until recently, Zr-POMs, like Zr-based heterogeneous catalysts, were associated mostly with homolytic oxidation mechanisms.^32^ However, the recently discovered highly selective alkene epoxidation in the presence of the Lindqvist-type Zr-POM, (Bu_4_N)_6_[{W_5_O_18_Zr(μ-OH)}_2_] (**{ZrW**_**5**_**}**_**2**_), provided strong evidence that Zr(IV) is able to activate H_2_O_2_ heterolytically.^46^ Using experimental and computational methods, we implicated an electrophilic oxygen transfer mechanism for alkene epoxidation, where a monomeric Zr-hydroperoxo species, [W_5_O_18_Zr(η^2^-OOH)]^3–^ (**ZrOOH**), acts as the real epoxidizing agent. At the same time, **{ZrW**_**5**_**}**_**2**_ reveals high activity in H_2_O_2_ decomposition in the absence of an organic substrate. From the traditional viewpoint, high epoxidation selectivity seems to be incompatible with high rates of H_2_O_2_ degradation.^47^ This prompted us to focus our attention on the H_2_O_2_ decomposition over **{ZrW**_**5**_**}**_**2**_ and investigate the reaction mechanism by means of spectroscopic, kinetic, test product, and computational tools. We have found evidence that H_2_O_2_ underdoes **{ZrW**_**5**_**}**_**2**_-catalyzed disproportionation with the evolution of singlet oxygen. To the best of our knowledge, this is the first demonstration of the ability of a Zr catalyst to accomplish H_2_O_2_ activation with the production of ^1^O_2_ and fulfill ene-type reactivity under base-free conditions.

## Experimental Section

### Materials

Acetonitrile (Panreac, HPLC grade) that was used as a solvent in catalytic reactions was dried and stored over activated 3 Å molecular sieves. The concentration of hydrogen peroxide (30, 50, or 77% in water) was determined iodometrically prior to use. All of the other chemicals were of AR grade, obtained commercially from Sigma-Aldrich and used as received without further purification.

### Synthesis of POMs

The syntheses of tetrabutylammonium (TBA) salts of **{ZrW**_**5**_**}**_**2**_,^48^ (Bu_4_N)_2_[W_5_O_18_Zr(H_2_O)_3_] (**ZrW**_**5**_),^46^ (Bu_4_N)_6_[(μ-η^2^:η^2^-O_2_){ZrW_5_O_18_}_2_] (**{ZrW**_**5**_**}**_**2**_**(O**_**2**_**)**),^46^ (Bu_4_N)_8_[{PW_11_O_39_Zr(μ-OH)}_2_] (**{PW**_**11**_**Zr(OH)}**_**2**_),^31^ (Bu_4_N)_3_[(CH_3_O) TiW_5_O_18_] (**TiW**_**5**_),^49,50^ and (Bu_4_N)_4_[(NbW_5_O_18_)_2_O] ({**NbW**_**5**_**}**_**2**_**O**)^51^ were carried out following the literature protocols. The compounds were characterized by elemental analysis and IR and multinuclear NMR spectroscopy (see the SI).

### H_2_O_2_ Decomposition

The decomposition of H_2_O_2_ (0.2 M) was studied in the absence of an organic substrate at 50 °C in CH_3_CN (3 mL) in the presence of **{ZrW**_**5**_**}**_**2**_ (0.004 M). Aliquots of 0.2 mL were taken periodically during the reaction course, and the H_2_O_2_ concentration was determined by iodometric titration. Two or three experiments were carried out in parallel in temperature-controlled glass vessels under vigorous stirring (500 rpm).

#### Reaction Order in the Catalyst

The concentration of POM was varied in the range of 0.001–0.006 M. The concentration of H_2_O_2_ was held constant (0.2 M).

#### Reaction Order in H_2_O_2_

The initial H_2_O_2_ concentration was varied in the range of 0.05–0.4 M. The concentration of water in these experiments was kept constant (1.41 M) by the addition of corresponding amounts of H_2_O. The concentration of POM was 0.004 M.

#### Reaction Order in H_2_O

The initial concentration of water was varied from 0.71 to 6.26 M. Other parameters were held constant: [H_2_O_2_] = 0.2 M and [POM] = 0.004 M.

#### Determination of Activation Energies

The temperature dependence of the H_2_O_2_ decomposition was studied in the range of 25–70 °C in CH_3_CN using the following reaction conditions: [H_2_O_2_] = 0.2 M and [POM] = 0.004 M.

### Initial Rate Determination and Evaluation of the Rate Law

The initial rate method was employed to determine the reaction orders. Initial rates were calculated as d[H_2_O_2_]/d*t* at *t* = 0. The rate law was derived by applying a steady-state approximation to concentrations of all active species or by using a quasi-equilibrium approximation. For a detailed description of the kinetic modeling procedure and derivation of the rate law, see the Supporting Information (SI).

### Kinetic Modeling

The kinetic modeling was done in Python 2.7 using library numpy 1.16.6. The sums of the squares of the difference between the experimental and theoretical values were minimized. Optimization was performed using the Canonical PSO algorithm^52^ with parameters of φ = 4.1 and χ = 0.729.

### Catalytic Oxidation of Test Substrates

Catalytic oxidations were performed under vigorous stirring (500 rpm) in thermostated glass vessels. Each experiment was reproduced two to three times, and the average value was reported. The catalytic oxidations of tetramethylethylene (TME, 2,3-dimethyl-2-butene) and α-terpinene were initiated by the addition of H_2_O_2_ (0.1–0.3 mmol) to a solution of substrate (0.1 mmol) in 1 mL of acetonitrile containing **{ZrW**_**5**_**}**_**2**_ or another POM catalyst (0.004 mmol or 0.008 mmol of active heterometal) at 27 °C. The oxidation products were identified by ^1^H NMR, gas chromatography–mass spectrometry (GC–MS), and a comparison of their GC retention times with those of authentic (hydro) peroxides prepared using a conventional sodium molybdate catalyst^53^ (Figure S1). ^1^H NMR characteristics of the principal products, 2,3-dimethyl-3-buten-2-hydroperoxide (from TME) and ascaridole (from α-terpinene), were identical to those reported in the literature^54,55^ (see the SI). The product yields and substrate conversions were quantified by gas chromatography (GC) using biphenyl as an internal standard. For α-terpinene, GC analyses of the reaction mixture before and after reduction with an excess of PPh_3_ revealed no changes in the peroxide product yield, while the TME-derived hydroperoxide product was reduced to 2,3-dimethyl-3-buten-2-ol.

### Stoichiometric and Quasi-Stoichiometric Interaction of **{ZrW_5_}_2_O_2_** with α-Terpinene

Reactions between α-terpinene and the peroxo complex **{ZrW**_**5**_**}**_**2**_**O**_**2**_ (isolated or prepared in situ from **{ZrW**_**5**_**}**_**2**_ and H_2_O_2_) were performed at 27 °C in dry acetonitrile ([POM] = 0.0025 or 0.0125 M, [substrate] = 0.0125 M). The reaction course was monitored using GC.

### EPR Studies

Experiments with 5,5-dimethyl-1-pyrroline *N*-oxide (DMPO) and 2,2,6,6-tetramethyl-4-piperidone (4-oxo-TEMP) spin traps were carried out at room temperature under an Ar atmosphere. To a reaction mixture containing 0.2 μmol of POM and 20 μmol of H_2_O_2_ in 100 μL of CH_3_CN, 5.4 μmol of DMPO was added. In the case of experiments with 4-oxo-TEMP, 20 μmol of the spin trap was added to the reaction mixture containing 1 μmol of POM and 0.1 mmol of H_2_O_2_ in 100 μL of CH_3_CN under an Ar atmosphere. Aliquots of 30 μL were withdrawn immediately, placed on a flat quartz capillary, and analyzed by EPR at room temperature. For comparison, EPR spectra were recorded using POM solutions and a spin trap without the addition of H_2_O_2_ under the same conditions.

EPR experiments without spin traps were performed by using a solution of POM (0.012 mmol) in acetonitrile (200 μL) and 77% aqueous H_2_O_2_ (0.048 mmol). Once EPR tubes were prepared, they were immediately frozen in liquid nitrogen and then analyzed by EPR.

### Instrumentation and Methods

^1^H NMR spectra were recorded on a Bruker Avance 300 spectrometer operating at 300.0 MHz using 5 mm o.d. glass NMR tubes (0.5 mL solution volume) and referenced to residual CHD_2_CN at δ 1.97 ppm in CD_3_CN solvent. ^31^P, ^93^Nb, ^17^O, and ^183^W NMR spectra were recorded at 161.67, 97.94, 54.24, and 16.67 MHz, respectively, on an Avance-400 Brüker spectrometer using a high-resolution multinuclear probe head with 10 mm o.d. (3 mL solution volume) sample tubes. ^17^O NMR spectra were recorded at a natural ^17^O abundance (0.037%). Chemical shifts, δ, were referenced to 85% H_3_PO_4,_ NbCl_5_, H_2_O, and Na_2_WO_4_ for ^31^P, ^93^Nb, ^17^O, and ^183^W NMR spectra, respectively. FT-IR spectra (in KBr pellets) were recorded by using a Cary 660 FTIR spectrometer (Agilent Technologies). GC analyses were performed using a Chromos GH-1000 gas chromatograph equipped with a flame ionization detector and a quartz capillary column (30 × 0.25 mm^2^) filled with BPX5. GC–MS analyses were carried out using an Agilent 7000B system with an Agilent 7000 triple–quadrupole mass-selective detector and a GC Agilent 7890B apparatus (quartz capillary column 30 m × 0.25 mm/–5 MS). EPR spectra were measured on a CMS 8400 EPR spectrometer at 9.4 GHz, with a modulation frequency of 100 kHz and a modulation amplitude of 5 G. Frozen solution EPR measurements were conducted in a quartz finger Dewar filled with liquid nitrogen (−196 °C). A toluene solution of TEMPO (2 × 10^–3^ M) was used as an external standard. The exact EPR parameters of the detected species were obtained by spectra simulation using the *Easyspin* program.^56^

### Computational Details

DFT calculations were performed with *Gaussian 16*, rev. A03 software^57^ at the B3LYP level of theory.^58−60^ The LANL2DZ basis set and associated pseudopotentials^61^ were used for W and Zr atoms whereas the remaining atoms were described by the 6-31g(d,p) basis set.^62−64^ Geometry optimizations were full and without any symmetry constraints, and solvent effects of acetonitrile were included in both geometry optimizations and energy calculations by means of the IEF-PCM implicit solvation model^65^ as implemented in *Gaussian 16* using standard parameters (ε = 35.688). This level of theory has been proven to be accurate and reliable enough to study the reactivity concerning POMs and their transition-metal-substituted analogues, always showing a high degree of consistency with experimental outcomes and kinetic studies.^66−69^ Also, we have recently benchmarked the employed level of theory against other computational methods, assessing the size of the basis set and the dispersion effects, for the alkene epoxidation catalyzed by [α-B-SbW_9_O_33_(^*t*^BuSiO)_3_Ti(O^*i*^Pr)]^3–^ complex.^69^ The standard-state correction of 1.89 kcal mol^–1^ was applied to the free energy of all species to account for the conversion from the reference state of 1 atm used in Gaussian calculations to the standard state of 1 mol L^–1^ at 25 °C. A data set collection of the optimized structures for the most representative species is available in the ioChem-BD repository^70^ (see the Supporting Information).

## Results and Discussion

### General Regularities of H_2_O_2_ Decomposition over Zr-POM

The activity of **{ZrW_5_}_2_** in the decomposition of H_2_O_2_ was systematically investigated in the absence of an organic substrate. Taking into account the solubility of TBA salts of POM, acetonitrile was used as the solvent. The rate of H_2_O_2_ decomposition in the absence of any catalyst was negligible. Dimer **{ZrW_5_}_2_** revealed high catalytic activity in this reaction, much higher than that of Ti- and Nb-substituted Lindqvist tungstates (Figure S2).

**Figure 1:**
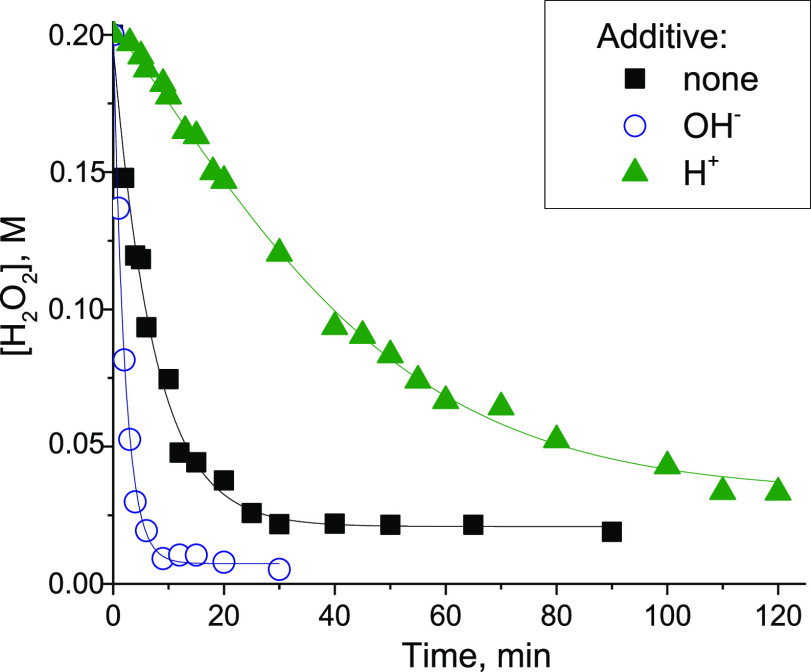
H_2_O_2_ decomposition in the presence of **{ZrW**_**5**_**}**_**2**_. Reaction conditions: 0.008 M Zr, 0.008 M HClO_4_,or Bu_4_NOH (if added), 0.2 M H_2_O_2_ (30%), and 3 mL of CH_3_CN at 50 °C.

Another interesting feature of the **{ZrW**_**5**_**}**_**2**_-catalyzed H_2_O_2_ decomposition was the pronounced rate-retarding effect of protons. The addition of 1 equiv of mineral acid (HClO_4_) greatly slowed down the H_2_O_2_ degradation rate, while the addition of base, on the contrary, accelerated the reaction (Figure 1). A similar effect of protons on the H_2_O_2_ unproductive decomposition was recently documented for Zr-MOFs.^34^

Note that the epoxidation of cyclohexene with H_2_O_2_ in the presence of **{ZrW**_**5**_**}**_**2**_ exhibited the opposite trend.^46^ The reaction was accelerated with the addition of acid, leading to a significant improvement of the oxidant utilization efficiency, while 1 equiv of Bu_4_NOH practically deactivated the catalyst. FT-IR spectroscopy revealed the disappearance of the 730 cm^–1^ feature attributed to the Zr–O(H)–Zr bond in the **{ZrW**_**5**_**}**_**2**_ dimer after the catalytic reactions; however, the main vibrations of the Lindqvist **ZrW**_**5**_ structure remained (Figure S3).

**Figure 2:**
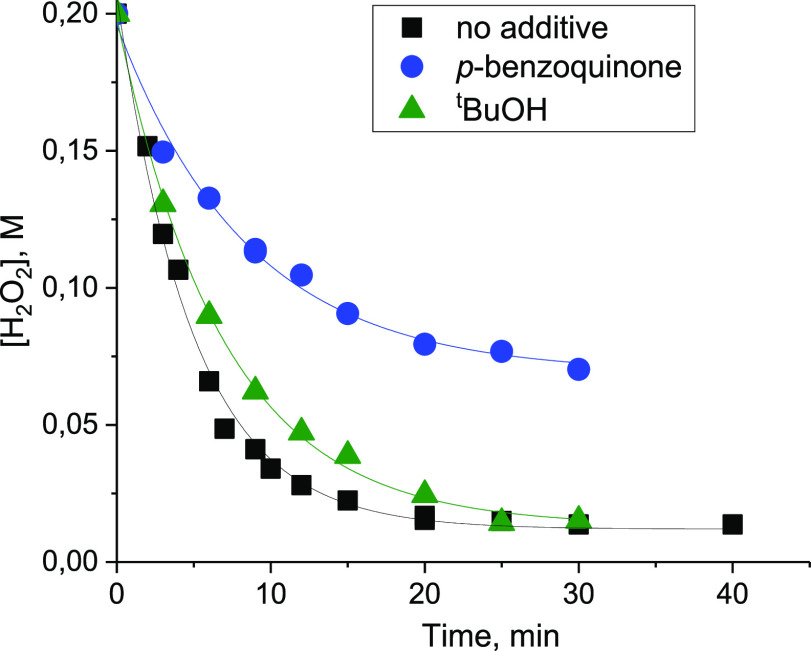
Influence of ^*t*^BuOH and *p*-benzoquinone on the rate of H_2_O_2_ decomposition in the presence of **{ZrW**_**5**_**}**_**2**_. Reaction conditions: 0.008 M Zr, 0.2 M H_2_O_2_ (30%), 0.002 M scavenger (if added), 3 mL of CH_3_CN, and 50 °C.

The effect of radical scavengers, ^*t*^BuOH (quencher for ^•^OH) and *p*-benzoquinone (quencher for ^•^O_2_^–^ radicals), was then investigated. While the influence of ^*t*^BuOH was minor, the impact of quinone was significant (Figure 2), indicating that superoxide radicals are certainly involved in the H_2_O_2_ decomposition process and the radical chains are relatively long.

### EPR Studies

To further investigate the H_2_O_2_ decomposition mechanism, EPR spectroscopy was employed. Frozen solution (−196 °C) EPR spectra of the sample **{ZrW**_**5**_**}**_**2**_ + H_2_O_2_ (CH_3_CN, [**{ZrW**_**5**_**}**_**2**_]:[H_2_O_2_] = 1:4) recorded immediately after reagent mixing displayed a signal with orthorhombic *g*-value anisotropy (*g*_1_ = 2.0375, *g*_2_ = 2.0121, and *g*_3_ = 2.0053; Figure S4). This signal resembles those assigned earlier to zirconium–superoxide species formed upon ZrO_2_ treated with H_2_O_2_.^21,22,71^ Note that [Zr]–O_2_^•^ can exist in equilibrium with the free superoxide HO_2_^•^. Hence, it is reasonable to suggest the formation of superoxide radicals in the **{ZrW**_**5**_**}**_**2**_/H_2_O_2_ system. This fully agrees with the fact that **{ZrW**_**5**_**}**_**2**_-catalyzed H_2_O_2_ decomposition is greatly decelerated by the addition of *p*-benzoquinone, a well-known superoxide scavenger (Figure 2).

DMPO, the diamagnetic spin-trap molecule, is widely used for the detection of ^•^OH and ^•^O_2_^–^ radicals.^72−78^ Since hydroxyl and superoxide radicals are often formed simultaneously during H_2_O_2_ dismutation, experimental EPR signals of DMPO-^•^O_2_^–^ and DMPO-^•^OH adducts may overlap.^79,80^ Based on the literature,^72,75,79,80^ we may assume that the EPR spectrum (Figure S5) recorded immediately after the addition of DMPO to the sample **{ZrW**_**5**_**}**_**2**_ + H_2_O_2_ (CH_3_CN, [H_2_O_2_]:[Zr] = 50) displays resonances characteristic of DMPO-^•^O_2_^–^ species, although some contribution of DMPO-^•^OH cannot be ruled out.

**Figure 3:**
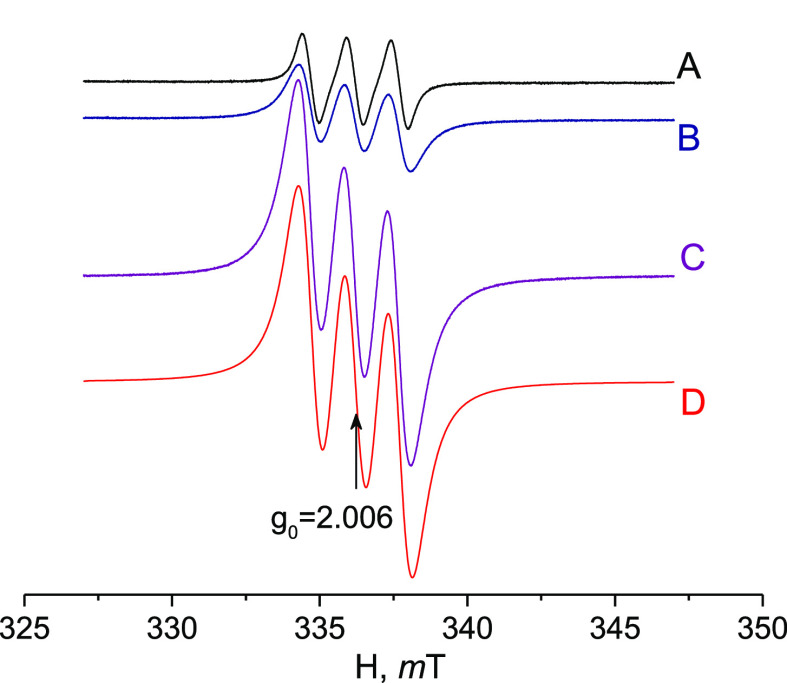
Liquid solution (25 °C) EPR spectra of the sample **{ZrW**_**5**_**}**_**2**_/H_2_O_2_/4-oxo-TEMP (CH_3_CN, [Zr]:[H_2_O_2_]:[4-oxo-TEMP] = 1:50:10, [Zr] = 0.02 M): (A) immediately after 4-oxo-TEMP addition; (B) sample in A, stored for 25 min at 25 °C; (C) sample in A, stored for 60 min at 25 °C; and (D) – simulated spectrum (C) (*g* = 2.006, A(^14^N) = 1.48 mT, and lwpp = [0.28 0.85]).

Sterically hindered 4-oxo-TEMP has long been used for singlet-oxygen-selective detection.^81−84^ The reaction between 4-oxo-TEMP and ^1^O_2_ gives the 4-oxo-TEMPO nitroxide radical displaying a characteristic 1:1:1 triplet signal in the EPR with the hyperfine splitting constant A(^14^N) = 1.50 mT.^81,84^ Liquid solution EPR spectra recorded immediately and 1 h after 4-oxo-TEMP addition to sample **{ZrW**_**5**_**}**_**2**_ + H_2_O_2_ (CH_3_CN, [H_2_O_2_]:[Zr] = 50) are presented in Figure 3. Three strong equal-intensity resonances (A(^14^N) = 1.48 mT) attributed to the 4-oxo-TEMPO nitroxide radical clearly indicate the formation of singlet oxygen in the **{ZrW**_**5**_**}**_**2**_/H_2_O_2_ system.

### Kinetics of H_2_O_2_ Decomposition over Zr-POM and the Rate Law

Kinetic curves of H_2_O_2_ degradation over **{ZrW**_**5**_**}**_**2**_ revealed no induction period. Light or molecular oxygen produced no effect on the reaction rate, which discarded any photochemical process or a radical chain process with the participation of O_2_.

**Figure 4:**
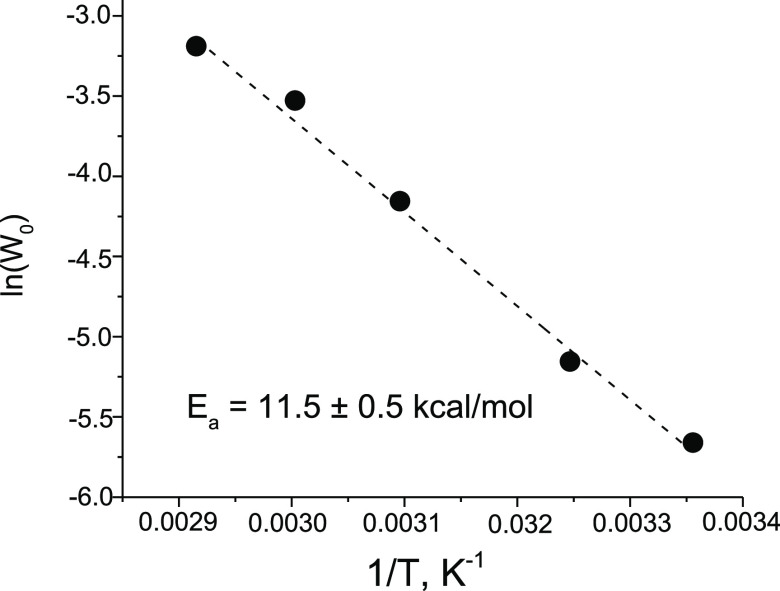
Arrhenius plot for H_2_O_2_ decomposition in the presence of **{ZrW**_**5**_**}**_**2**_ in CH_3_CN. Reaction conditions: 0.008 M Zr, 0.2 M H_2_O_2_ (30%), and 3 mL CH_3_CN.

The rate of **{ZrW**_**5**_**}**_**2**_-catalyzed H_2_O_2_ degradation showed a typical Arrhenius dependence (Figure 4), which implies no alteration of the rate-limiting step over the studied temperature range. The value of the apparent activation energy *E*_a_ estimated from the Arrhenius plot (11.5 kcal mol^–1^) turned out to be considerably lower than the values of *E*_a_ previously determined for Ti- and Nb-monosubstituted tungstates of the Lindqvist structure (14.6 and 16.7 kcal mol^–1^, respectively)^50^ and a bit higher than *E*_a_ reported for H_2_O_2_ decomposition over ZrO_2_ (8–10 kcal mol^–1^).^36,37,39^

**Figure 5:**
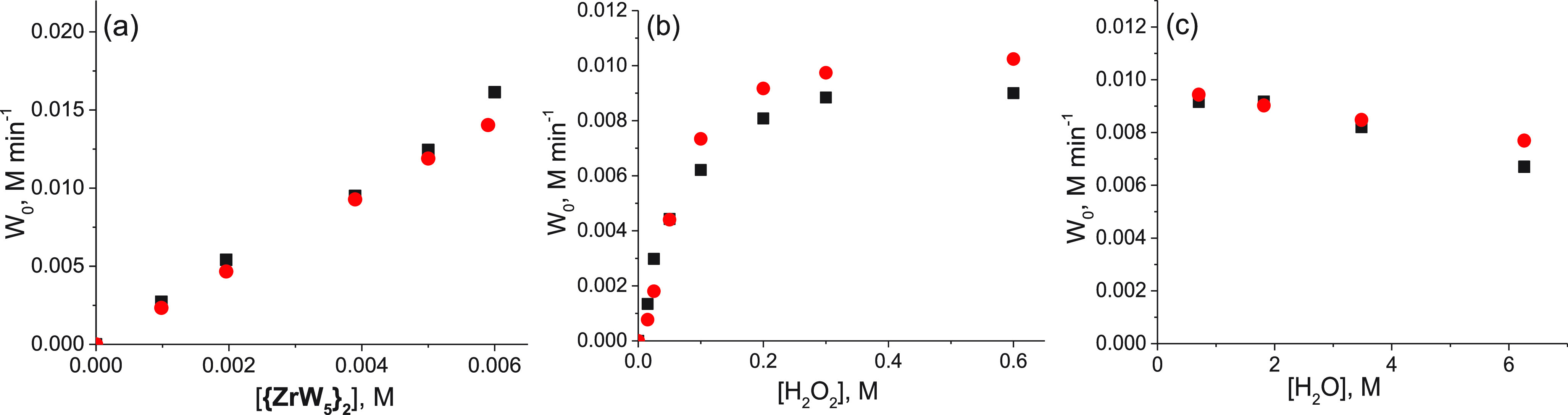
Experimental kinetic data (■) and fitted eq S37 (red circle) plots of the initial rate (*W*_0_) of H_2_O_2_ decomposition in the presence of **{ZrW**_**5**_**}**_**2**_ (50 °C) versus the concentration of (a) **{ZrW**_**5**_**}**_**2**_, (b) H_2_O_2_, and (c) H_2_O. For reaction conditions, see the Experimental Section.

The dependences of the initial rate of H_2_O_2_ decomposition on the concentrations of **{ZrW**_**5**_**}**_**2**_, H_2_O_2_, and water are plotted in Figure 5. The reaction is first order in the catalyst **{ZrW**_**5**_**}**_**2**_ (Figure 5a) and has a variable order (1–0) with respect to the H_2_O_2_ concentration (Figure 5b). The addition of extra water to the reaction system slightly decelerated the H_2_O_2_ decomposition rate (Figure 5c).

On the basis of the literature devoted to the mechanisms of H_2_O_2_ decomposition (see the Introduction), at least three alternative reaction pathways can be envisaged for interaction and transformation of H_2_O_2_ over dimeric **{ZrW**_**5**_**}**_**2**_. (See the SI for a detailed description.) The first two stages might be the same for all three mechanisms and include dimer monomerization and the formation of hydroperoxo complex **ZrOOH**. Then this hydroperoxo species (or a Zr-peroxo [HW_5_O_18_Zr(η^2^-OO)]^3–^ protonated at a bridging Zr–O–W site, which exists in equilibrium with **ZrOOH**)^46^ can interact with the second H_2_O_2_ molecule, leading either directly to decomposition products (mechanism **1**) or producing a diperoxo species followed by an inner-sphere process resulting in the peroxide degradation products (mechanism **2**). Alternatively, **ZrOOH** can dissociate with the formation of ZrO^•^ and ^•^OH radicals (mechanism **3**). All of the mechanisms are first order with respect to the concentrations of **{ZrW**_**5**_**}**_**2**_, which might be a consequence of the rapid step of dimer monomerization in the presence of water. Mechanisms **1** and **2a** (mechanism **2** under the assumption that the diperoxo species is not stable; see the SI for details) propose that the reaction order with respect to H_2_O_2_ must be equal to 1 or 2. However, the experimentally observed reaction order was 1 changing to 0 at high concentrations of H_2_O_2_ (Figure 5b). On the other hand, mechanisms **2b** and **3** suggest varied first to zero reaction order in H_2_O_2_, which is in agreement with the kinetic experiments. Despite this similarity, mechanisms **2b** and **3** can be distinguished by using reciprocal coordinates 1/*W*_0_ – 1/[H_2_O_2_] (Figure S6). Therefore, the experimental kinetic data better match the rate law derived from mechanism **2** under the assumption that both monoperoxo and diperoxo species are relatively stable and exist in chemical equilibria with ZrOH and ZrOOH, respectively (mechanism **2b**). Fitting the rate law deduced for mechanism **2b** (eq S37) to the experimental data is shown in Figure 5.

Unfortunately, it is difficult (if possible, at all) to determine the value of K_2_ with good accuracy. (See the Supporting Information for a detailed discussion.) On the other hand, the kinetic modeling study made it possible to estimate the value of *K*_4_ (mechanism **2b**) as ca. 20. Therefore, the formation of a diperoxo complex is certainly plausible if our kinetic model is correct. Note that H_2_O_2_ decomposition catalyzed by group III metals [M(H_2_O)_*n*_]^3+^ (M = Al, Ga, In, Sc, Y, or La) was suggested to involve the formation of a diperoxo species, *cis*-[M(H_2_O)_*n*−2_(OOH)(H_2_O_2_)]^2+^, with subsequent homolytic cleavage of the O–O bond, leading to the generation of hydroxyl radicals,^16,17^ while triperoxo molybdenum complexes were implicated as the active species responsible for H_2_O_2_ disproportionation to ^1^O_2_ and H_2_O.^85^

Taking into account that previous results demonstrated that the hydrolysis of **{ZrW**_**5**_**}**_**2**_ is a rather slow process (or thermodynamically unfavorable),^46,86^ we cannot also exclude mechanism **4** where the Zr dimer interacts with H_2_O_2_ directly to form a dimeric peroxo complex, [(μ-η^2^:η^2^-O_2_){ZrW_5_O_18_}_2_]^6–^ (**{ZrW**_**5**_**}**_**2**_**(O**_**2**_**)**). Then it can further react with the second H_2_O_2_ molecule to produce peroxide decomposition products or, alternatively, give a dimeric diperoxo species, followed by an inner-sphere peroxide transformation into the peroxide degradation products. Note that, in this case, the rate law will be similar to that derived from mechanism **2b** (see the SI for details). All four mechanisms will be analyzed computationally, providing an atomistic and an energetic description and identifying several active species for hydrogen peroxide decomposition (see below).

### Peroxidation of Test Substrates

**Scheme 1:**
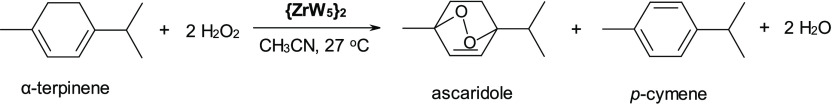
Oxidation of α-Terpinene with H_2_O_2_ in the Presence of **{ZrW**_**5**_**}**_**2**_

**Scheme 2:**
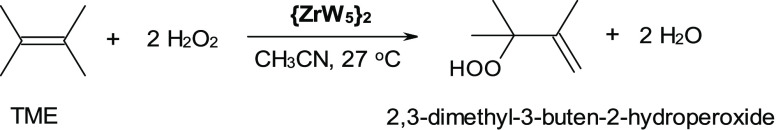
Oxidation of TME with H_2_O_2_ in the Presence of **{ZrW**_**5**_**}**_**2**_

To distinguish among different mechanisms of H_2_O_2_ activation, the oxidation of test organic substrates, indicative of the involvement of ^1^O_2_, was carried out. Oxidation of α-terpinene and TME with H_2_O_2_ in the presence of catalytic amounts of **{ZrW**_**5**_**}**_**2**_ revealed the formation of endoperoxide ascaridole and 2,3-dimethyl-3-butene-2-hydroperoxide, respectively (Schemes 1 and 2), the products typical of singlet oxygen participation.^39,45,53^ In particular, the oxidation of α-terpinene with a 2-fold excess of H_2_O_2_, corresponding to a stoichiometric amount of ^1^O_2_, at nearly room temperature afforded ascaridole in 40% yield at 50% substrate conversion, while the yield of the main byproduct *p*-cymene was 4%. The optimization of the reaction conditions resulted in a 90% yield of ascaridole (see Table S1 and Figure S7). Under optimized conditions, TME gave the characteristic hydroperoxide in a 70% yield. This allowed us to suggest that **{ZrW**_**5**_**}**_**2**_ possesses a pronounced ability to generate singlet oxygen upon interaction with hydrogen peroxide. It is noteworthy that the results were the same when the reaction was performed in the dark or under an atmosphere of argon.

Additives of 4-oxo-TEMP resulted in the appearance of a pronounced induction period on the kinetic curve of α-terpinene oxidation to ascaridole (Figure S8), which further supports the participation of singlet oxygen in this reaction. Oppositely, the addition of the radical scavengers ^*t*^BuOH and *p*-benzoquinone had no effect on the ascaridole formation rate and attainable yield (Figure S9), suggesting that ^1^O_2_ is generated directly upon interaction of H_2_O_2_ with **{ZrW**_**5**_**}**_**2**_. The addition of base (Bu_4_NOH in methanol) slowed the reaction but did not affect the selectivity to endoperoxide (Table S1, entry 6). On the other hand, selectivity to ascaridole dropped significantly and attained only 10% in the presence of small amounts of HClO_4_ (Table S1, entry 7). The latter is not surprising if we recall that acid additives prevent the dismutation of H_2_O_2_ over **{ZrW**_**5**_**}**_**2**_ (Figure 1). Note that the effect of acid on the alkene epoxidation in the presence of **{ZrW**_**5**_**}**_**2**_ was, on the contrary, positive,^46^ which indicates that different active zirconium intermediates are involved in the two oxidation processes.

The dimeric peroxo complex **{ZrW**_**5**_**}**_**2**_**(O**_**2**_**)**, while being inactive toward α-terpinene and TME under stoichiometric conditions, can be activated by the addition of quasi-stoichiometric amounts of H_2_O_2_ (Table 1)_._ The same reaction outcome could be achieved using both the peroxo complex and parent **{ZrW**_**5**_**}**_**2**_ if an appropriate amount of H_2_O_2_ is added (entries 4 and 6 in Table 1). These results suggest that a Zr complex with two peroxo (or hydroperoxo) moieties is responsible for the generation of singlet oxygen and peroxidation reactions. Note that the key role of a dimeric diperoxo species in the H_2_O_2_ degradation over **{ZrW**_**5**_**}**_**2**_ was supported by the kinetic study (mechanism **4** in the SI), although the participation of a monomeric diperoxo species could not be excluded (mechanism **2b**). Interestingly, dimeric diperoxo zirconium complexes have been described for the Keggin and some other structures,^87−89^ but they have not been isolated for the Lindqvist structure.

**Table 1:** Stoichiometric and Quasi-stoichiometric Interaction of **{ZrW**_**5**_**}**_**2**_**O**_**2**_ (or **{ZrW**_**5**_**}**_**2**_ + H_2_O_2_) with α-Terpinene^a^

				Yield, %	
Entry	POM	α-Terpinene:POM:H_2_O_2_ molar ratio	α-Terpinene conversion, %	Ascaridole	*p*-Cymene
1	**{ZrW**_**5**_**}**_**2**_**(O**_**2**_**)**	1:0.2	0	0	0
2	**{ZrW**_**5**_**}**_**2**_**(O**_**2**_**)**	1:0.2:0.4	30	8	2
3	**{ZrW**_**5**_**}**_**2**_**(O**_**2**_**)**	1:0.2:2.4	100	83	3
4	**{ZrW**_**5**_**}**_**2**_**(O**_**2**_**)**	1:1:1	50	27	4
5	**{ZrW**_**5**_**}**_**2**_	1:1:1	10	1	1
6	**{ZrW**_**5**_**}**_**2**_	1:1:2	50	27	4

a Reaction conditions: 0.0125 M α-terpinene, 0.5 mL CH_3_CN, and 27 °C.

The ene-type reactivity of **{ZrW**_**5**_**}**_**2**_ was unique among the other POMs studied (Table 2). Neither Nb- nor Ti-substituted Lindquist tungstates were able to produce ascaridole. More importantly, a Zr-substituted dimer of the Keggin structure, **{PW**_**11**_**Zr(OH)}**_**2**_, and Lindqvist monomer **{ZrW**_**5**_**}** revealed the formation of *p*-cymene instead of ascaridole, indicating no capability of generating singlet oxygen (Table 2). Interestingly, only traces of the characteristic peroxide products were recently found in the oxidation of TME and α-terpinene over Zr-MOFs.^90^

**Table 2:** α-Terpinene Oxidation with H_2_O_2_ over Various POMs^a^

		Selectivity, %	
POM	α-Terpinene conversion, %	Ascaridole	*p*-Cymene
–	5	20	50
**{ZrW**_**5**_**}**_**2**_	41	78	7
**ZrW**_**5**_	34	4	38
**{PW**_**11**_**Zr(OH)}**_**2**_	59	2	27
**(NbW**_**5**_**)**_**2**_**O**	96	2	25
**TiW**_**5**_	94	2	24

a Reaction conditions: 0.1 M α-terpinene, 0.004 M Zr, Nb or Ti, 0.1 M H_2_O_2_ (30%), 1 mL CH_3_CN, 27 °C, and 5 h.

### IR Studies

FT-IR studies on the catalyst recovered after completion of the reaction showed that the IR spectra (and therefore the catalyst state) were affected by the reaction conditions, specifically, the concentration of H_2_O_2_ and the oxidant addition mode (Figure S10). Similar changes in the IR spectra were previously observed for Zr peroxo complexes upon increasing the amount of H_2_O_2_ used for their preparation and have been rationalized by the formation of a dimeric μ-η^2^:η^2^-peroxo complex **{ZrW**_**5**_**}**_**2**_**(O**_**2**_**)** at a low H_2_O_2_ excess (H_2_O_2_/Zr < 20) and a monomeric η^2^-hydroperoxo species **ZrOOH** at H_2_O_2_/Zr > 35.^46^ The spectra of the catalyst remaining after α-terpinene oxidation with 2 equiv of H_2_O_2_ and with the dropwise addition of 3 equiv of H_2_O_2_ were nearly identical and close to the IR spectrum of **{ZrW**_**5**_**}**_**2**_ recovered after H_2_O_2_ decomposition in the absence of any organic substrate. In turn, these IR spectra resemble that of the dimeric peroxo complex **{ZrW**_**5**_**}**_**2**_**(O**_**2**_**)** reported in our previous work (see Figure S10),^46^ indicating that the stepwise addition of the oxidant produces a milder effect on the catalyst and disfavors monomerization of the dimeric structure (the latter is manifested by the higher-energy shift of the W=O vibrations).^46^ This agrees well with the results of the kinetic study, which supported mechanism **4** through a dimeric peroxo Zr species.

### DFT Calculations on the H_2_O_2_ Decomposition Mechanism

**Figure 6:**
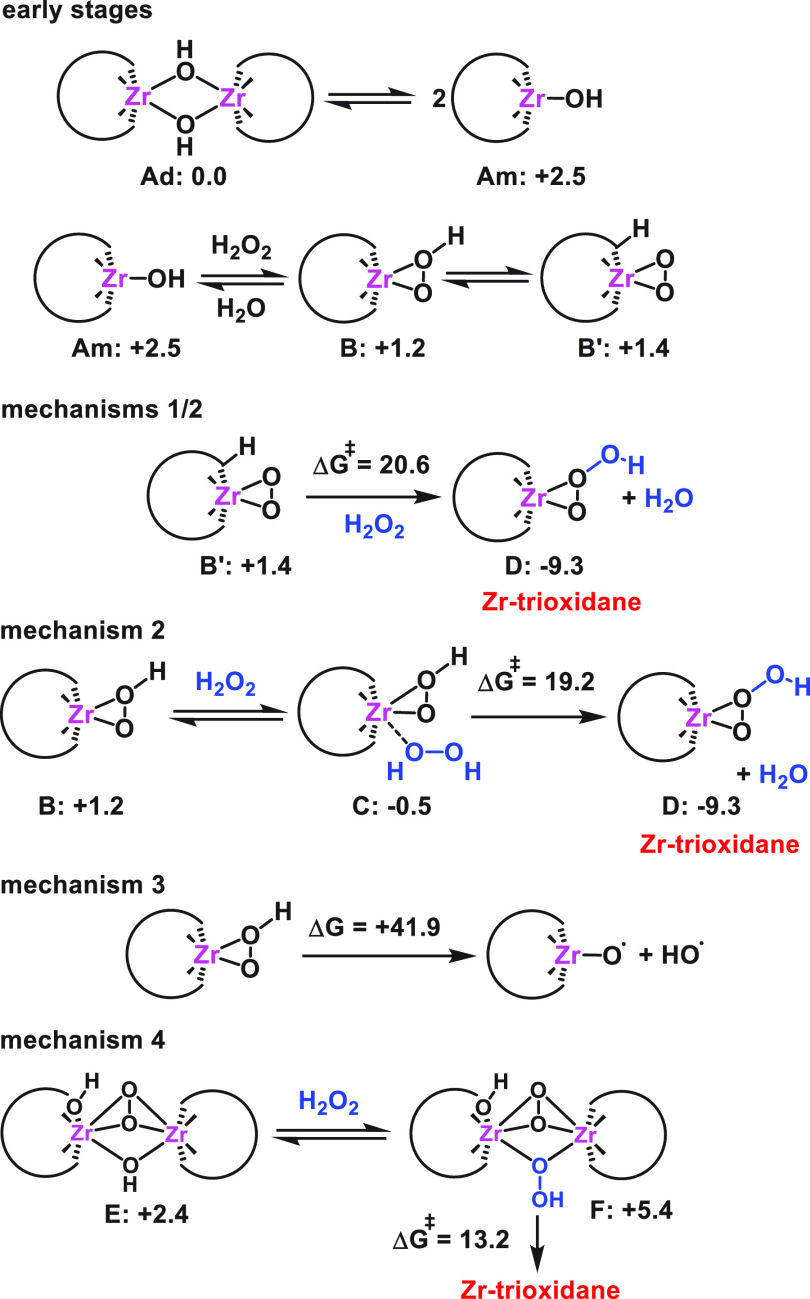
Possible reaction mechanisms for the H_2_O_2_ decomposition catalyzed by the [{W_5_O_18_Zr(μ-OH)}_2_]^6–^ (**Ad**) anion involving monomeric (mechanisms **1** to **3**) and dimeric (mechanism **4**) Zr-POM species, analyzed computationally. Relative Gibbs free energies and free-energy barriers are given in kcal·mol^–1^.

To better understand the mechanism responsible for the H_2_O_2_ decomposition, we have analyzed computationally all of the variants proposed in the kinetic studies (mechanisms **1** to **4**, see Figure 6 and the SI), providing a full atomistic and energetic description that is consistent with the experimental findings. Unlike previous studies for related systems in which only homolytic bond-cleavage processes are considered,^16,17,91−99^ we herein propose novel and plausible pathways involving the heterolytic H_2_O_2_ activation that leads to the formation of singlet oxygen, as observed experimentally. Concurrently, we have also characterized a competitive homolytic pathway that explains the formation of ^•^OH and ^•^O_2_^–^ radicals. As it is not possible to unequivocally discern whether the active species are monomeric or dimeric structures on the sole basis of experimental data, we have analyzed both types. First, we focused on the reaction pathways proceeding through monomeric species, as shown in Figure 6 (mechanisms **1** to **3**). Starting from the dimeric anion [{W_5_O_18_Zr(μ-OH)}_2_]^6–^ (**Ad**), the common early stages consist of monomerization and the subsequent heterolytic activation of H_2_O_2_ through a rapid proton transfer to the hydroxo ligand to release a water molecule, forming the Zr-hydroperoxo complex [W_5_O_18_Zr(η^2^-OOH)]^3–^ (**B**), which is in equilibrium with the Zr-peroxo complex [HW_5_O_18_Zr(η^2^-OO)]^3–^ (**B′**) protonated at a bridging Zr–O–W site. These initial steps have been previously studied in detail for Zr-substituted POMs,^46,100,101^ showing that all of the species involved lay within a narrow range of energies and that they have mild to low free-energy barriers for interconversion (20.6, 3.6, and 3.8 kcal·mol^–1^ for monomerization, H_2_O_2_ activation, and peroxo formation, respectively, as shown in Figure S11). Our calculations show that in both mechanisms **1** and **2** the Zr-peroxo and Zr-hydroperoxo species can interact with a second H_2_O_2_ molecule, decomposing it to form an unprecedented Zr-trioxidane intermediate [W_5_O_18_Zr(η^2^-OO(OH))]^3–^ (**D**) through moderate free-energy barriers (Figure 6). As we will discuss below, the Zr-trioxidane intermediate **D** can then easily evolve to generate either singlet oxygen or superoxide radicals, which have both been observed experimentally (vide supra). Conversely, we can discard the homolytic O–O bond breaking of the hydroperoxo moiety to produce the radical species [W_5_O_18_Zr(O^•^)]^3–^ and ^•^OH (mechanism **3**) because of the prohibitively high free-energy cost (+41.9 kcal·mol^–1^).

**Figure 7:**
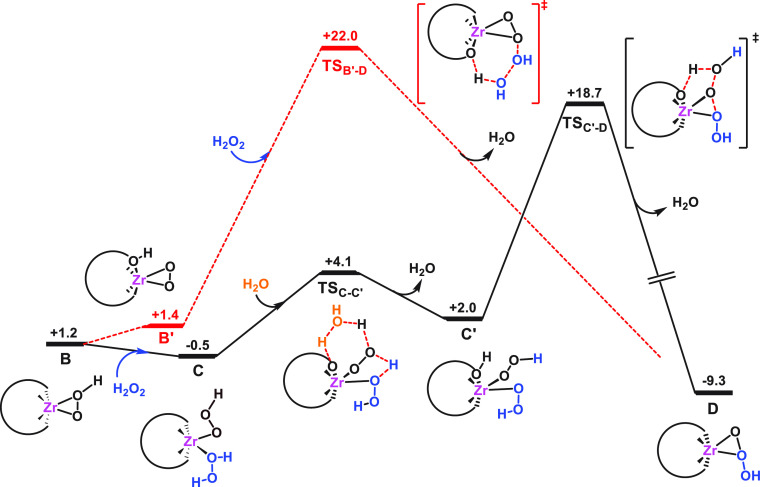
Gibbs free-energy profile (kcal·mol^–1^) for the formation of monomeric Zr-trioxidane intermediate **D**, via inner- and outer-sphere pathways (black solid lines and red dashed lines respectively). All free energies are relative to those of the initial dimeric structure **Ad**.

**Figure 8:**
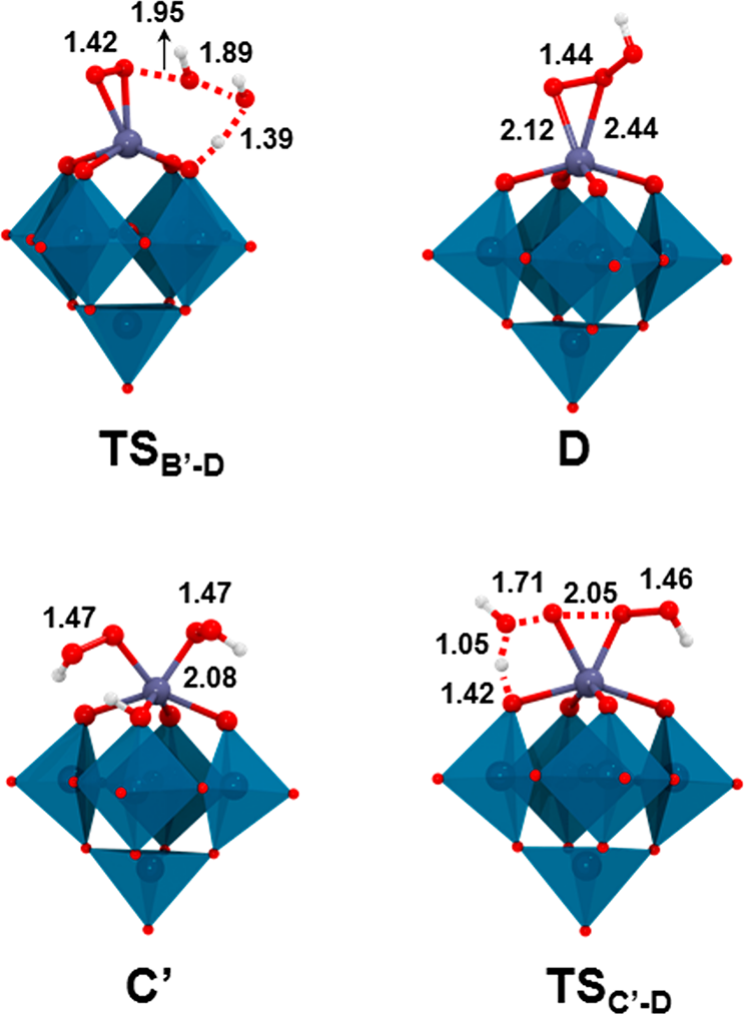
DFT-optimized geometries for the most relevant species in the reaction profile for the formation of monomeric Zr-trioxidane species. Selected distances are given in Å. Color code: Zr (violet), W (cyan), O (red), and H (white).

Figure 7 shows the calculated free-energy profile for the formation of Zr-trioxidane intermediate (**D**) through an outer- and an inner-sphere mechanism (red dashed and black solid lines, respectively). In line with mechanism **1**, the Zr-peroxo moiety in **B′** can perform an outer-sphere nucleophilic attack^102^ on an oxygen atom of a second H_2_O_2_ molecule, promoting the heterolytic O–O bond cleavage in the latter, which can occur concomitantly with a proton transfer from the POM framework to the leaving hydroxyl group to generate a water molecule (**TS**_**B′-D**_ in Figure 8). Here we cannot rule out that during the Zr-peroxo attack explicit solvent molecules participate in the polarization of the O–O bond instead of the mobile POM proton or in addition to it. Also, we note that alkene epoxidation involves a different active species, the Zr-hydroperoxo complex **B**, from which there is an electrophilic oxygen transfer to the substrate.^46^ This could explain the opposite effect of the acid additives in H_2_O_2_ decomposition and alkene epoxidation, in which the reactions are slowed or accelerated, respectively. In fact, the ambiphilic character of some metal-peroxide complexes has been previously observed.^103−105^ The computed process occurs through an affordable free-energy barrier of 20.6 kcal·mol^–1^ from **B′** and generates the Zr–trioxidane species **D**, Zr[η^2^-OO(OH)], and a water molecule, in an overall exergonic process (by more than 10 kcal mol^–1^, see Figure 7).

Alternatively, the second H_2_O_2_ molecule can coordinate to the flexible Zr center, forming the “diperoxo” complex [W_5_O_18_Zr(OOH)(H_2_O_2_)]^3–^ (**C**), which then yields the Zr-trioxidane complex **D** via an inner-sphere hydrogen peroxide degradation (mechanism **2**, as illustrated by solid lines in Figure 7). The participation of complexes similar to **C** ([L_*m*_M(OOH)(H_2_O_2_)]^*n*+^) has been proposed for the H_2_O_2_ decomposition by group III metals.^16,17^ Moreover, the computed free energy associated with the process (−1.7 kcal·mol^–1^, from **B** to **C**) is like the equilibrium constant *K*_4_ determined by kinetic modeling (estimated Δ*G* = −1.8 kcal·mol^–1^ for *K*_4_ = 20, see above). From **C**, the formation of the Zr-dihydroperoxo intermediate [HW_5_O_18_Zr(OOH)_2_]^3–^ (**C′**, see Figures 7 and 8) proceeds through a rapid proton transfer from the coordinated H_2_O_2_ to the POM framework (bridging Zr–O–W oxygen), assisted by the hydroperoxo ligand and a water molecule acting as proton shuttles (**TS**_**C–C′**_ in Figure 7) that results in a low free-energy barrier (Δ*G*^⧧^ = 4.6 kcal mol^–1^). Finally, the two α-oxygens of hydroperoxo ligands couple to form the new O–O bond of trioxidane, while one of the O–OH bonds is cleaved to form a water molecule out of the leaving hydroxyl moiety and the proton from the POM framework (see **TS**_**C′-D**_ in Figure 8). The computed overall free-energy barrier for the H_2_O_2_ decomposition through the monomeric, inner-sphere mechanism (**C** → **TS**_**C′-D**_) is 19.2 kcal·mol^–1^, with the transition state **TS**_**C′-D**_ lying 3.3 kcal·mol^–1^ below that of the outer-sphere path (**TS**_**B′-D**_). Thus, we can conclude that the inner-sphere pathway (mechanism **2**) is preferred over the outer-sphere pathway (mechanism **1**), although we cannot rule out that both mechanisms are operative, given the small free-energy difference between them.

**Figure 9:**
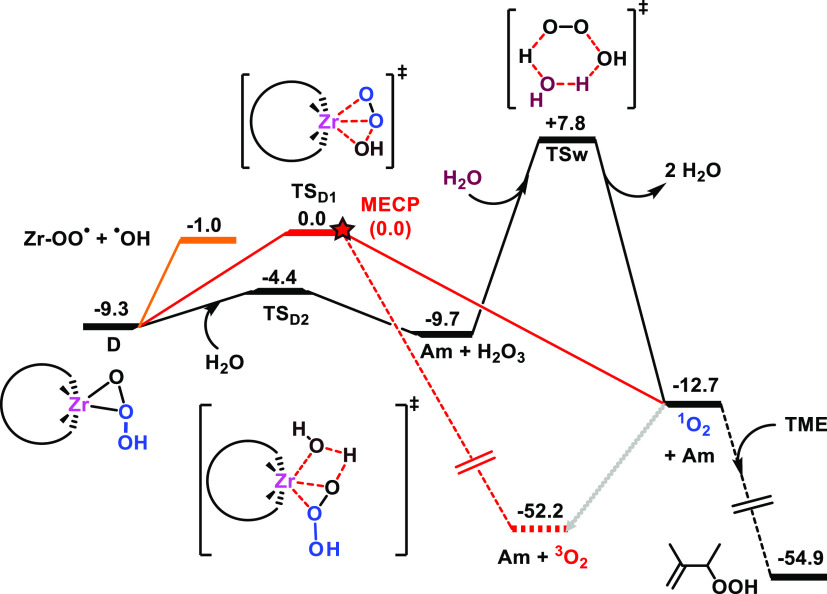
Free-energy profile (kcal·mol^–1^) for the evolution of monomeric Zr-trioxide intermediate **D** to produce singlet oxygen (black and red lines) and superoxide radicals (orange lines). Dashed lines denote the triplet state, and the red star stands for a minimum-energy crossing point (MECP) between the singlet and the triplet potential energy surfaces.

As anticipated above, the Zr-trioxidane intermediate **D** can then decompose through different pathways to give a singlet oxygen molecule or superoxide radicals (Figure 9). Specifically, species **D** can rapidly release singlet molecular oxygen and regenerate Zr-hydroxo species **Am** through transition state **TS**_**D1**_, overcoming a small free-energy barrier of 9.3 kcal·mol^–1^. Along the pathway, the system may hop from the singlet to the triplet potential energy surfaces, yielding **Am** and the more stable triplet molecular oxygen ^3^O_2_. We found a minimum-energy crossing point (MECP) very close to **TS**_**D1**_ in the free-energy landscape since their energies and geometries are almost identical. The transition state in the triplet-state surface could not be located, as geometry optimization algorithms brought the structure to products. The transition from the singlet to the triplet surface to produce ^3^O_2_ without the intermediacy of ^1^O_2_ could occur to some extent during the reaction, reducing the yield of the oxidation of organic substrates described above. Alternatively, in the presence of water, the hydrolysis of Zr-trioxidane complex **D** can release trioxidane (H_2_O_3_) into the medium, overcoming a very low free-energy barrier of 4.9 kcal mol^–1^ (**TS**_**D2**_ in Figure 9) and also regenerating **Am**, which can be reincorporated into the main catalytic cycle. Trioxidane is known to decompose in the presence of water into a water molecule and singlet molecular oxygen ^1^O_2_.^106−109^ At the employed level of theory, the free-energy barrier for the H_2_O_3_ decomposition is 17.5 kcal mol^–1^ (**TSw** in Figure 9), which is close to the enthalpy barriers previously found,^109^ indicating that the entropic contribution for this process is small. Although the free-energy barrier for H_2_O_3_ decomposition is feasible under the working conditions, the reverse free-energy barrier is very low (**Am** + H_2_O_3_ → **D**, Δ*G*^⧧^ = 5.3 kcal·mol^–1^), suggesting that the formation of H_2_O_3_ is reversible and that the reaction yielding ^1^O_2_ proceeds preferentially through the lower-energy path involving the direct decomposition of Zr-trioxidane (**TS**_**D1**_ in Figure 9). We also note that an additional water molecule could participate in the hydrolysis of Zr-trioxide, as we have previously reported for the hydrolysis of dimeric Zr structures^101^ and of the Zr–H_2_O_2_ complex.^46^ Nevertheless, in this case, adding a water molecule to reactive species does not provide new mechanistic insight (see Figure S12), while the specific nature of intermediates might depend on the macroscopic environment.^100,101^

The singlet oxygen (^1^O_2_) is known to undergo radiative decay to the ground-state triplet oxygen (^3^O_2_) on the microseconds timescale.^110^ Nonetheless, ^1^O_2_ has shown the ability to activate allylic C–H bonds in organic molecules;^111^ therefore, it is reasonable to think that if ^1^O_2_ reaches a TME substrate molecule in solution then it can react through an ene-like mechanism, as found by Houk et al.^112^ In the absence of organic substrates, we expect the heterolytic pathways for H_2_O_2_ decomposition to produce water and ^3^O_2_ through either the minimum-energy crossing point or the radiative decay of ^1^O_2_. This mechanism would also explain the formation of 2,3-dimethyl-3-butene-2-hydroperoxide from the oxidation of TME with H_2_O_2_, which is computed to be strongly exergonic by more than 40 kcal mol^–1^ (Figure 9). Also, we evaluated the effect of the homolytic O–O bond breaking on the Zr-trioxidane complex [W_5_O_18_Zr(η^2^-OO(OH))]^3–^ (**D**) to yield the Zr-superoxide [W_5_O_18_Zr(OO^•^)]^3–^ and ^•^OH (Figure 9). The computed free-energy cost for this process (8.3 kcal·mol^–1^) is significantly less demanding than that from the Zr-hydroperoxo species (41.9 kcal·mol^–1^), thus becoming competitive with the heterolytic trioxide decomposition to give ^1^O_2_.

**Figure 10:**
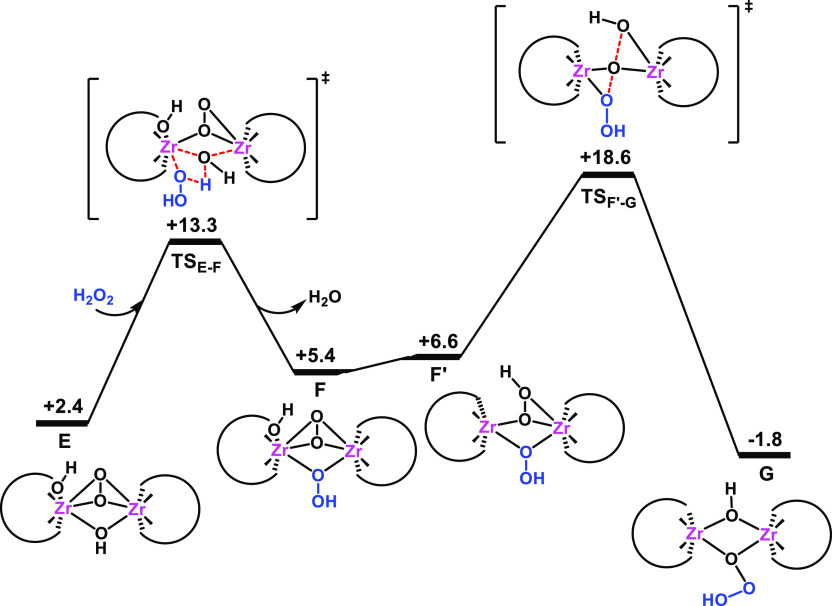
Gibbs free energy profile (kcal·mol^–1^) for the formation of dimeric Zr-trioxidane intermediate **G** via the inner-sphere pathway. All free energies are relative to those of structure **Ad**.

**Figure 11:**
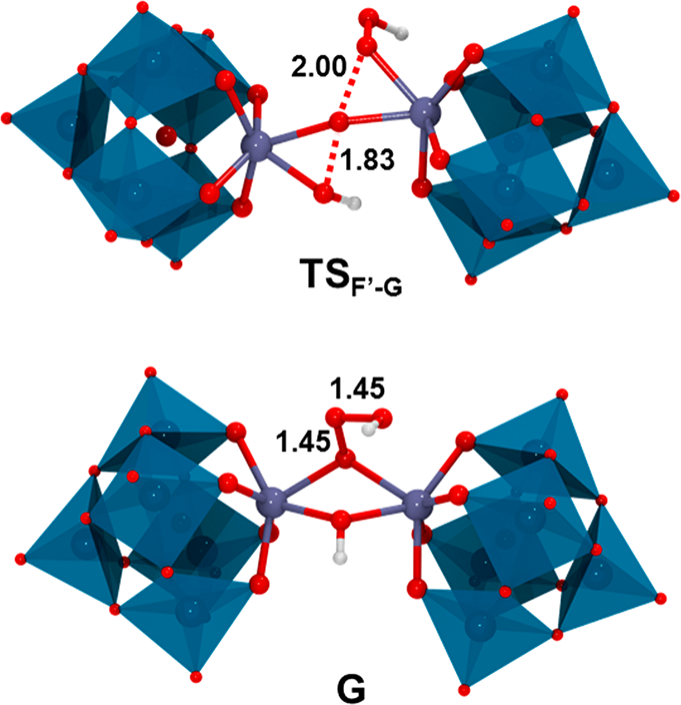
DFT-optimized geometries for the most relevant species in the reaction profile for the formation of the dimeric Zr-trioxidane species. Selected distances are shown in Å. Color code: Zr (violet), W (cyan), O (red), and H (white).

Our kinetic and spectroscopic observations (see above) suggest that the mechanism for H_2_O_2_ decomposition could also proceed through the active participation of dimeric peroxo Zr species (mechanism **4**). Previous DFT studies on the oxidation of organic substrates with H_2_O_2_ by group IV-metal-substituted POMs have shown that dimeric structures can also be active for oxygen-transfer processes when the substrates have low steric demand,^46,67,113,114^ as is the case of H_2_O_2_. Thus, we next investigated the H_2_O_2_ decomposition promoted by the dimeric species. In a previous contribution, we have analyzed in detail the first activation of H_2_O_2_ by the interaction with the **Ad** dimer that results in different type of peroxo-bridging, dimeric Zr species.^46^ From these complexes, here we found a favorable pathway for the activation of a second H_2_O_2_ molecule to give a dimeric Zr-trioxidane species from the [(μ-η^2^:η^2^-O_2_)(μ-OH)(H){ZrW_5_O_18_}_2_]^6–^ (**E**) complex, which bears one peroxo and one hydroxo bridging ligand (see Figure 10). The Supporting Information shows other computationally characterized pathways that are less energetically feasible or unlikely (see Figures S13 and S14). The proposed mechanism is analogous to that described for inner-sphere peroxide degradation by monomeric species (mechanism **2** in Figure 7). First, a second H_2_O_2_ molecule interacts with the μ-hydroxo ligand in **E**, releasing water and forming a bridging hydroperoxo ligand in intermediate [(μ-η^2^:η^2^-O_2_)(μ-OOH)(H){ZrW_5_O_18_}_2_]^6–^ (**F**) after getting over a low free-energy barrier of 10.9 kcal·mol^–1^. Then, an intramolecular proton transfer from the POM framework to the peroxo moiety yields the dihydroperoxo intermediate [(μ-OOH)_2_{ZrW_5_O_18_}_2_]^6–^ (**F′**), from which the two hydroperoxo ligands react through the transition state **TS**_**F′-G**_ (Figure 11), giving access to the hydroxo-trioxidane, dimeric species **G**, [(μ-OH)(μ-OO(OH)){ZrW_5_O_18_}_2_]^6–^ (see Figures 10 and 11). The overall process to form **G** is exergonic (−1.8 kcal·mol^–1^) and shows a moderate, overall free-energy barrier (18.6 kcal·mol^–1^, **Ad** → **TS**_**F′-G**_) that is somewhat lower than those computed for monomeric pathways (22.0 and 19.2 kcal·mol^–1^). For dimeric species, the outer-sphere mechanism whereby the peroxo group in **E** nucleophilically attacks an incoming, second H_2_O_2_ molecule (Figure S14) was energetically unfeasible, with a free-energy barrier of 28.6 kcal·mol^–1^.

**Scheme 3:**
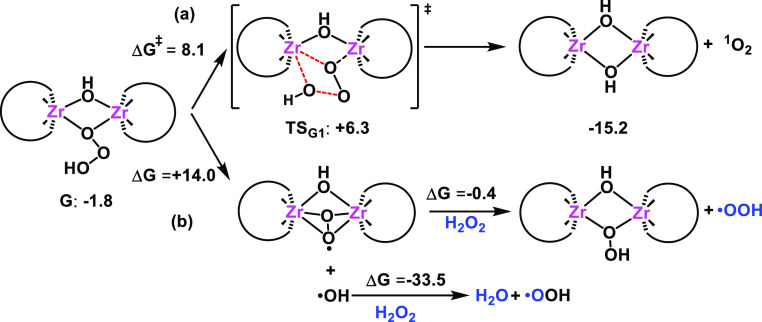
Possible Reaction Pathways for the Decomposition of the Dimeric Zr-Trioxidane Intermediate **G** to Produce a Singlet Oxygen Molecule via a Heterolytic Mechanism (a) and Superoxide Radicals via Homolytic O–O Bond Breaking (b) Gibbs free energies, relative free energies with respect to species **Ad**, and free-energy barriers are given in kcal·mol^–1^.

Similar to the monomeric path, the trioxidane intermediate **G** can decompose heterolytically to form ^1^O_2_ and recover the dimeric form of the catalyst **Ad** (Scheme 3a). This process occurs with a low free-energy barrier of 8.1 kcal·mol^–1^ through transition state **TS**_**G1**_ in which the α- and β-oxygens of the trioxidane ligand form the ^1^O_2_ product and the γ-O(H) moiety turns into the hydroxyl bridging ligand. As in the case of the monomeric path, here we also assume that a fraction of the reaction mixture can hop from the singlet to the triplet surface to produce ^3^O_2_. From **G**, the homolytic decomposition of the trioxidane ligand to yield the radical products Zr-superoxide and ^•^OH is also feasible with a free-energy cost of 14.0 kcal·mol^–1^ (Scheme 3b). The Zr-superoxide can further react with H_2_O_2_ to produce free hydroperoxyl radicals ^•^OOH and regenerate the dimeric peroxo intermediate (Δ*G* = −0.4 kcal·mol^–1^). In parallel, hydroxyl radicals ^•^OH can also react with hydrogen peroxide to give water and again (^•^OOH) as the radical product^115,116^^117^ in a favorable exergonic process of −33.5 kcal·mol^–1^. Note that the radical ^•^OOH can coexist as the corresponding conjugate base ^•^O_2_^–^ in aqueous or polar solvents.^118^ Interestingly, the generation of the singlet oxygen from trioxidane intermediate **G** is energetically preferred over that of the superoxide radicals (8.1 vs 14.0 kcal·mol^–1^), in qualitative agreement with the selectivity observed in the oxidation of α-terpinene and TME substrates (Schemes 1 and 2 and Table 1). Moreover, this trend is inverted for monomeric species (9.3 vs 8.3 kcal·mol^–1^ for singlet oxygen and superoxide generation, respectively), in line with the experimental decrease in singlet oxygen formation, which becomes the minor product, in moving from the dimer to the monomer (see Table 2). Experimentally, the ene-type reactivity is observed only for **{ZrW**_**5**_**}**_**2**_ and **{ZrW**_**5**_**}**_**2**_**(O**_**2**_**)** dimers, and the IR spectra indicate the retention of a dimeric structure after the oxidation reaction. Note, however, that the accurate evaluation of the selectivity is difficult due to the intrinsic limitations of DFT in evaluating homolytic bond-breaking processes.

To summarize, we have computationally characterized three viable pathways for H_2_O_2_ decomposition (mechanisms **1**, **2**, and **4**), all of them involving the heterolytic activation of a first H_2_O_2_ molecule to yield a Zr-(hydro) peroxo species, followed by the heterolytic degradation of a second H_2_O_2_ molecule to form an unprecedented Zr-trioxidane intermediate. The latter species can then evolve through either a heterolytic or a homolytic O_β_–O_γ_ (H) bond cleavage to produce a singlet oxygen molecule or superoxide radicals, respectively. In all three pathways, the rate-determining step corresponds to the formation of the Zr-trioxidane species via heterolytic O–O bond cleavage of a peroxide group, a H_2_O_2_ molecule, or a Zr-OOH ligand in outer- and inner-sphere mechanisms, respectively. The computed free-energy barriers lay in a narrow range (22.0, 19.2, and 18.6 kcal·mol^–1^ for mechanisms **1**, **2**, and **4**, respectively), and it is not possible to rule out the participation of any of them. Kinetic simulations, using rate constants derived from DFT free-energy results via transition-state theory, set the following reactivity prevalence: monomeric outer-sphere (mechanism **1**) ≪ monomeric inner-sphere (mechanism **2**) < dimeric inner-sphere (mechanism **4**), as detailed in Table S3. Moreover, in agreement with the experiments collected in Figure 5c, simulations showcase an acceleration of the reaction rate for H_2_O_2_ decomposition when decreasing the water content.

To further validate our mechanistic proposal, we compared the DFT results with the experimental Arrhenius activation energy for H_2_O_2_ decomposition in the presence of **{ZrW**_**5**_**}**_**2**_. The computed zero-point-corrected energy barriers for the corresponding rate-determining steps in mechanisms **1**, **2**, and **4** are 13.9, 10.1, and 8.8 kcal·mol^–1^, respectively. From the Boltzmann distribution of the three pathways, the computed, weighted-average activation barrier is 9.2 kcal·mol^–1^, which is rather close to the experimental *E*_a_ value of 11.5 kcal·mol^–1^. Moreover, we compared the barrier obtained for **{ZrW**_**5**_**}**_**2**_ with those for Ti- and Nb-substituted Lindqvist tungstate analogues, assuming that in all cases either heterolytic or homolytic H_2_O_2_ decomposition proceeds through TM-trioxidane formation, which is the rate-determining step. The computed barriers are 9.2, 14.7, and 18.6 kcal·mol^–1^ for Zr-, Ti-, and Nb-substituted POMs, in good agreement with the experimental values of 11.5, 14.6, and 16.7 kcal·mol^–1^, respectively. (See Figure S15 and Table S2 for details.) Note that for second-row transition metal Nb the inner-sphere mechanism is preferred, whereas for the first-row Ti metal the outer-sphere attack is more favorable. In fact, we did not succeed in characterizing an inner-sphere mechanism for Ti, most likely due to the stiffer nature of its coordination sphere compared to those of Nb and Zr.^46,50^ Another consequence is that the activation barrier increases on going from the Zr(IV)- to Ti(IV)-substituted POM, despite both metal centers having the same oxidation state. In going from Zr(IV) to Nb(V) within the same transition-metal row, the metal fragment becomes less nucleophilic^46^ and consequently the nucleophilic attack of the Nb-hydroperoxo moiety on the second H_2_O_2_ molecule becomes less favored, increasing the apparent activation energy. Overall, we can conclude that the central role of a metal-trioxidane intermediate that we have identified for the H_2_O_2_ decomposition by the Zr(IV)-substituted Lindqvist anion can also apply to other TM-substituted POMs such as Ti(IV) and Nb(V).

## Conclusions

In this work, we provide the first atomistic, full characterization of the reaction mechanism responsible for the hydrogen peroxide decomposition promoted by transition-metal-based catalysts using the Zr(IV)-monosubstituted dimeric Lindqvist tungstate anion [{W_5_O_18_Zr(μ-OH)}_2_]^6–^, namely, **{ZrW**_**5**_**}**_**2**_. Using kinetic, spectroscopic, and computational tools, we propose a novel mechanism that involves the formation of an unprecedented Zr-trioxidane [Zr-η^2^-OO(OH)] key intermediate upon nucleophilic attack of a Zr-peroxo species, which is generated after the heterolytic activation of a first H_2_O_2_ molecule, to a second molecule of H_2_O_2_, promoting the heterolytic O–O cleavage in the latter. This process was found to be the rate-determining step of the whole H_2_O_2_ decomposition reaction. The as-formed Zr-trioxidane intermediate is highly reactive and can evolve heterolytically to produce singlet oxygen (^1^O_2_) or homolytically to yield superoxide radicals (^•^O_2_^–^), showcasing that the formation of the main byproducts in catalytic oxidations with H_2_O_2_ is indeed related to the H_2_O_2_ disproportionation reaction. Moreover, we show that the formation of a TM-trioxidane intermediate is also feasible for Ti- and Nb-substituted POMs, in which the trioxidane species would evolve preferentially through the homolytic path rather than through ^1^O_2_ generation. Experimentally, we have demonstrated that the **{ZrW**_**5**_**}**_**2**_ complex exhibits high activity in the dismutation of hydrogen peroxide, producing singlet oxygen along with superoxide radicals. Depending on the reaction conditions and the organic substrate nature, **{ZrW**_**5**_**}**_**2**_ can realize either epoxidation or ene-type catalytic oxidation. So far, **{ZrW**_**5**_**}**_**2**_ is the only known representative of TM-substituted POMs capable of ^1^O_2_ generation and related ene-type catalytic oxidation. Overall, we expect the herein reported in-depth understanding of the reaction pathways that govern the unproductive decomposition of H_2_O_2_ to inspire the design of unique selective oxidation catalysts.

## Supporting Information Available

Additional experimental and computational details: ^1^H NMR, FT-IR, and EPR spectra; HPLC-ICP-AES chromatograms; synthesis procedures; catalytic results; additional energy profiles; results from microkinetic simulations; DFT structures of relevant intermediates and transition states; and Cartesian coordinates of computed structures (PDF)
