# Supplementary material for: Resolving the Mechanism for H2O2 Decomposition over Zr(IV)-Substituted Lindqvist Tungstate: Evidence of Singlet Oxygen Intermediacy
Source: ACS Catal. 2023 Jul 24;13(15):10324–39. doi: 10.1021/acscatal.3c02416 (PMC10407852; doi:10.1021/acscatal.3c02416)
Supplement: Supplementary file 1 — cs3c02416_si_001.pdf [file cs3c02416_si_001.pdf]

Supporting Information

Resolving the mechanism for H_2O_2 decomposition over Zr(IV)-substituted Lindqvist tungstate. Evidence of singlet oxygen intermediacy

Nataliya V. Maksimchuk[†] *Jordi Puiggalí-Jou,*[#] *Olga V. Zalomaeva,*[†] *Kirill P. Larionov,*[†] *Vasilii Yu. Evtushok,*[†] *Igor E. Soshnikov,*[†] *Albert Solé-Daura,*[#] *Oxana A. Kholdeeva,*[†,*] *Josep M. Poblet,*[#] *and Jorge J. Carbó*[#,*]

† Boreskov Institute of Catalysis, Pr. Lavrentieva 5, Novosibirsk 630090, Russia

# Departament de Química Física i Inorgànica, Universitat Rovira i Virgili, 43005 Tarragona, Spain

* Corresponding authors: khold@catalysis.ru; j.carbo@urv.cat

Table of Contents

**Experimental details**	p.
**Characterization of POMs**	4
**^{1}H NMR spectra of ascaridole and 2,3-dimethyl-3-buten-2-hydroperoxide**	5
**Alternative mechanisms of H_2O_2 decomposition over Zr-POM and rate law determination**	6
**Figure S1.** Oxidation of (a) α-terpinene and (b) tetramethylethylene with H_2O_2 (35% in water). Chromatograms of the reaction mixtures after the oxidation over **{ZrW5}**$_2$ in comparison with a blank reaction and oxidation in the presence of Na_2MoO_4.	13
**Figure S2**. H_2O_2 decomposition in the presence of **NbW$_5$**, **TiW$_5$**, and **{ZrW$_5$}$_2$**.	14
**Figure S3.** FT-IR spectra (in KBr pellets) of **{ZrW$_5$}$_2$**: (A) initial dimer (B) after H_2O_2 decomposition in the presence of **{ZrW$_5$}$_2$**, (C) after H_2O_2 decomposition in the presence of **{ZrW$_5$}$_2$** and 1 equiv. of Bu_4NOH, (D) after H_2O_2 decomposition in the presence of **{ZrW$_5$}$_2$** and 1 equiv. of $HClO_4$/	14
**Figure S4.** Frozen solution (−196 °C) EPR spectra: (A) - 0.06 M solution of **{ZrW$_5$}$_2$** in CH_3CN; (B) - sample in (A) immediately after addition of 77% H_2O_2 (2 equiv. per Zr); (C) - simulated spectrum for sample in (B); (D) - sample in (B) after 25 min storing at room temperature.	15
**Figure S5.** Experimental (A) and simulated (B) EPR spectra of the DMPO–radical adduct formed upon interaction of **{ZrW$_5$}$_2$** with H_2O_2 (0.004 *M* Zr, 0.2 M H_2O_2, 0.054 M DMPO, 100 μL CH_3CN, 25 °C).	15
**Figure S6.** Experimental plot of the initial rate of H_2O_2 decomposition in the presence of **{ZrW$_5$}$_2$** at 50 °C in double reverse coordinates ($1/W_0 – 1/[H_2O_2]$) with fitted linear (Eq. S39) and polynomial (Eq. S37) plots.	16
**Optimization of α-terpinene oxidation**	16
**Table S1.** α-Terpinene oxidation with H_2O_2 over **{ZrW$_5$}$_2$**.	17
**Figure S7.** The effect of H_2O_2 (50%) concentration on the ascaridole yield in α-terpinene oxidation in the presence of **{ZrW$_5$}$_2$**.	18
**Figure S8.** The effect of 4-oxo-TEMP on the rate of ascaridole formation in the α-terpinene oxidation with **{ZrW$_5$}$_2$**.	18
**Figure S9.** The influence of ^{t}BuOH and *p*-benzoquinone on the rate of ascaridole formation in the α-terpinene oxidation in the presence of **{ZrW$_5$}$_2$**.	19

**Figure S10.** FT-IR spectra (in KBr pellets) of **{ZrW$_5$}$_2$**: (A) initial dimer, (B) after α-terpinene oxidation with 0.2 M 50% H_2O_2, (C) after α-terpinene oxidation with 0.3 M 30% H_2O_2, (D) after α-terpinene oxidation with 0.3 M 50% H_2O_2 added dropwise, (F) ATR-FT-IR spectrum of $(Bu_4N)_6[(\mu-\eta^2:\eta^2-O_2)\{ZrW_5O_{18}\}_2]$.	19
**Figure S11.** Gibbs free-energy path of the monomerization and its 1st H_2O_2 activation. All free-energies are in kcal· mol^{-1} and are relative to the starting dimeric structure **Ad**.	20
**Figure S12.** Free-energy profile (kcal·mol^{-1}) for the evolution of monomeric Zr-trioxide intermediate **D** to produce singlet oxygen through a water mediated process where trioxidane is formed. All energies are relative to the initial dimeric specie **Ad**.	20
**Figure S13**. Alternative reaction pathways for the second H_2O_2 activation by Zr-dimeric species.	21
**Figure S14.** Representation of the **TS$_{E-G}$** involved in the outer-sphere H_2O_2 activation forming the dimeric trioxidane intermediate **G**.	22
**Figure S15.** Calculated Zero Point-corrected electronic energy barriers for H_2O_2 decomposition by Zr-, Ti-, and Nb-substituted Lindqvist POMs to yield TM-trioxidane intermediate.	22
**Table S2.** Comparison of the experimental activation energies (E_a) energies and calculated Zero Point-corrected electronic energy barriers ($\Delta E^{\ddagger}_{ZPE}$) for the rate-determining step of H_2O_2 decomposition reaction.	23
**Table S3.** Distribution of reaction paths for H_2O_2 decomposition by **Ad** at 50 °C derived from microkinetic modelling.	23
**Cartesian coordinates of the most representative optimized structures**	24

Experimental details

Characterization of POMs.

$(Bu_4N)_6[\{W_5O_{18}Zr(\mu\text{-}OH)\}_2]$ (**{ZrW$_5$}$_2$**). Anal. Calcd (%) for $C_{96}H_{218}N_6Zr_2W_{10}O_{38}$: C, 28.20; H, 5.33; N, 2.06; O, 14.88; Zr 4.47; W, 44.99. Found: C, 27.70; H, 5.57; N, 2.02; Zr, 4.58; W, 43.7. IR (KBr, 1000−400 cm^{-1}): 970 (sh, W=O), 945 (s, W=O), 881 (m), 812 (br, WOW), 731 (s, ZrOH), 645 (m), 625 (m), 557 (m), 430 (s), 419 (sh). ^{183}W NMR (ppm, in CD_3CN): 50.8 ($4W_{eq}$), 78.2 ($1W_{ax}$).

$(Bu_4N)_2[W_5O_{18}Zr(H_2O)_3]$ (**ZrW$_5$**). IR (1000–400 cm^{-1}): 962 (s, W=O), 883 (m), 832 (sh), 813 (s, WOW), 739 (sh), 690 (w), 665 (w), 636 (w), 592 (w), 420 (s).

$(Bu_4N)_6[(\mu\text{-}\eta^2\text{:}\eta^2\text{-}O_2)\{ZrW_5O_{18}\}_2]$ (**{ZrW$_5$}$_2$(O$_2$)**). Anal. Calcd (%) for $C_{96}H_{216}N_6Zr_2W_{10}O_{38}$: C, 28.20; H, 5.33; N, 2.06; Zr 4.47; W, 45.00. Found: C, 28.27; H, 5.60; N, 2.09; Zr, 4.40; W, 44.50. IR (ATR, 1000–400 cm^{-1}): 972 (sh, W=O), 947 (s, W=O), 881 (m), 800 (s, WOW), 781 (s), 629 (m), 567 (m), 494 (w), 475 (w), 419 (m). The presence of 0.5 peroxo groups per Zr was confirmed by titration with triphenylphosphine followed by monitoring with GC.

$(Bu_4N)_8[\{PW_{11}O_{39}Zr(\mu\text{-}OH)\}_2]$ (**{PW$_{11}$Zr(OH)}$_2$**). Anal. Calcd (%) for $C_{128}H_{294}N_8Zr_2W_{22}O_{82}$: C, 20.37; H, 3.90; N, 1.48; Zr, 2.42; W, 53.59. Found: C, 20.20; H, 3.85; N, 1.5; Zr, 2.33; W, 53.3. IR (1100–400 cm^{-1}): 1064, 965, 890, 807, 769 (ZrOZr), 685, 595, 514. ^{183}W NMR (ppm, 0.02 M in CD_3CN): δ -91.6 (2), -92.6 (2), -96.3 (1), -102.5 (2), -115.5 (2), -119.1 (2). ^{31}P NMR (ppm, 0.01 M in CH_3CN): δ -12.22. ^{183}W NMR (0.05 M in dry MeCN): δ -94.5(2), -95.5(2), -98.9(1), -105.5(2), -118.1(2), and -122.4(2). Potentiometric titration with methanolic TBAOH revealed 1.2 of acid proton per the molecule of POM.

$(Bu_4N)_3[(CH_3O)TiW_5O_{18}]$ (**TiW$_5$).** Anal. calcd (%) for $C_{49}H_{111}N_3TiW_5O_{19}$: C, 29.22; H, 5.55; N, 2.09. Found: C, 28.72; H, 5.60; N, 2.11. IR (KBr, 1200-400 cm^{-1}): 1152 (m, CO), 1096 (m, CO), 966 (w), 947 (s, W=O), 929 (w, sh), 904 (m), 883 (m), 777 (vs, WOW), 736 (sh, WOW), 592 (m, Ti-OC), 567 (sh), 537 (w, Ti-OC), 424 (s). ^{1}H NMR (ppm, in CD_3CN):4.10 (s, 3H, OCH_3), 3.23(m, 24H, NCH_2), 1.68 (m, 24H, CH_2), 1.44 (m, 24H, CH_2), 1.00 (t, 36H, CH_3). ^{17}O NMR (ppm, in CH_3CN):724 (W=O), 716 (W=O), 527 (TiOW), 393 (WOW), 383 (WOW), and –55 (μ6-O).

$(Bu_4N)_4[(NbW_5O_{18})_2O]$ (**{NbW$_5$}$_2$O**). Anal. Calcd (%) for $C_{64}H_{144}N_4Nb_2W_{10}O_37$: C, 21.44; H, 4.05; N, 1.56; O, 16.51; Nb, 5.18; W, 51.26. Found: C, 21.64; H, 4.07; N, 1.69; O, 16.37; Nb, 5.13; W, 50.2. IR (KBr, 1000−400 cm^{-1}): 994 (w, sh), 974 (s), 877 (m), 835 (w, sh), 812 (s), 733 (w, sh), 720 (m, sh), 695 (s, NbONb), 588 (m), 547 (m), 447 (s). ^{93}Nb NMR (ppm, in CH_3CN): −950.

^{1}H NMR spectra of ascaridole and 2,3-dimethyl-3-buten-2-hydroperoxide

Ascaridole ^{1}H NMR (400 MHz, $CDCl_3$, ppm): δ 0.9902 (d, J = 7.0 Hz, 6 H), 1.3696 (s, 3 H), 1.5112 (d, J = 9.5 Hz, 2 H), 1.88-1.95 (m, 1 H), 1.97-2.04 (m, 2 H), 6.4071 (d, J = 8.5, 1 H), 6.4905 (d, J = 8.5 Hz,1 H).

2,3-Dimethyl-3-buten-2-hydroperoxide ^{1}H NMR (400 MHz, $CDCl_3$, ppm): δ 1.3586 (s, 6 H), 1.8071 (s, 3 H), 4.96 (m, 1 H), 5.00 (m, 1 H), 7.4233 (s, 1 H).

Alternative mechanisms of H_2O_2 decomposition over Zr-POM and rate law determination

<u>Mechanism 1a</u>

$$M(OH)_2M \underset{}{\overset{K_1}{\rightleftharpoons}} 2MOH$$

$$MOH + H_2O_2 \underset{k_{-2}}{\overset{k_2}{\rightleftharpoons}} MOOH + H_2O$$

$$MOOH + H_2O_2 \xrightarrow{k_3} \text{products} \qquad \text{(slow step)}$$

Hereinafter, $M(OH)_2M$ is dimer **$\{ZrW_5\}_2$**, MOH – monomer **ZrW_5** existing in chemical equilibrium with $M(OH)_2M$ (Eq. S1), and MOOH is monomeric hydroperoxo complex **$Zr(OOH)W_5$**.

$$K_1 = \frac{[MOH]^2}{[M(OH)_2M]} \qquad \text{(S1)}$$

For **Mechanism 1a**, we assume that MOOH is not stable and disappears due to reverse transformation into MOH and/or reaction with H_2O_2. Therefore,

$$\frac{d[MOOH]}{dt} = k_2[MOH][H_2O_2] - k_{-2}[MOOH][H_2O] - k_3[MOOH][H_2O_2] = 0 \qquad \text{(S2)}$$

$$[MOOH] = \frac{k_2[MOH][H_2O_2]}{k_{-2}[H_2O]+k_3[H_2O_2]} \qquad \text{(S3)}$$

These approximations (in combination with the mass-balance equation S4) allow us to find all POM form ($M(OH)_2M$, MOH, and MOOH) concentration:

$$2[M(OH)_2M] + [MOH] + [MOOH] = 2[M(OH)_2M]_0 \qquad \text{(S4)}$$

$$\frac{2[MOH]^2}{K_1} + [MOH] + \frac{k_2[MOH][H_2O_2]}{k_{-2}[H_2O]+k_3[H_2O_2]} = 2[M(OH)_2M]_0 \qquad \text{(S4.1)}$$

$$[MOH] = \frac{K_1}{4}\left(\sqrt{\left(\frac{k_2[H_2O_2]}{k_{-2}[H_2O]+k_3[H_2O_2]}+1\right)^2 + \frac{16[M(OH)_2M]_0}{K_1}} - \left(\frac{k_2[H_2O_2]}{k_{-2}[H_2O]+k_3[H_2O_2]}+1\right)\right) \qquad \text{(S5)}$$

The reaction rate is:

$$W_0 = k_3[H_2O_2][MOOH] = k_3[H_2O_2]\frac{k_2[H_2O_2][MOH]}{k_{-2}[H_2O]+k_3[H_2O_2]} =$$

$$= \frac{k_3K_2K_1[H_2O_2]^2}{4(k_{-2}[H_2O]+k_3[H_2O_2])}\left(\sqrt{\left(\frac{k_2[H_2O_2]}{k_{-2}[H_2O]+k_3[H_2O_2]}+1\right)^2 + \frac{16[M(OH)_2M]_0}{K_1}} - \left(\frac{k_2[H_2O_2]}{k_{-2}[H_2O]+k_3[H_2O_2]}+1\right)\right) \qquad \text{(S6)}$$

If we assume that the major part of POM exists in the MOH form,

$$\left(\frac{k_2[H_2O_2]}{k_{-2}[H_2O]+k_3[H_2O_2]}+1\right) \gg \frac{16[M(OH)_2M]_0}{K_1} \qquad \text{(S7)}$$

we can simplify the right part of Eq. S6 by using the Taylor series:

$$\sqrt{\left(\frac{k_2[H_2O_2]}{k_{-2}[H_2O]+k_3[H_2O_2]}+1\right)^2+\frac{16[M(OH)_2M]_0}{K_1}} = \left(\frac{k_2[H_2O_2]}{k_{-2}[H_2O]+k_3[H_2O_2]}+1\right)\sqrt{1+\frac{16[M(OH)_2M]_0}{K_1\left(\frac{k_2[H_2O_2]}{k_{-2}[H_2O]+k_3[H_2O_2]}+1\right)^2}} \quad \text{(S8)}$$

$$\sqrt{\left(\frac{k_2[H_2O_2]}{k_{-2}[H_2O]+k_3[H_2O_2]}+1\right)^2+\frac{16[M(OH)_2M]_0}{K_1}} \approx \frac{1}{2}\left(\frac{k_2[H_2O_2]}{k_{-2}[H_2O]+k_3[H_2O_2]}+1\right)\left(1-\frac{16[M(OH)_2M]_0}{K_1\left(\frac{k_2[H_2O_2]}{k_{-2}[H_2O]+k_3[H_2O_2]}+1\right)^2}\right) \quad \text{(S9)}$$

The reaction rate after simplification is:

$$W_o = \frac{2k_2k_3[M(OH)_2M]_0[H_2O_2]^2}{(k_2+k_3)[H_2O_2]+k_{-2}[H_2O]} \quad \text{(S10)}$$

Therefore, **Mechanism 1a** is anticipated to give second reaction order in H_2O_2 when $[H_2O_2]$ is small and first reaction order in H_2O_2 when $[H_2O_2]$ is high.

Mechanism 1b

$$M(OH)_2M \overset{K_1}{\rightleftharpoons} 2MOH$$

$$MOH + H_2O_2 \overset{K_2}{\rightleftharpoons} MOOH + H_2O$$

$$MOOH + H_2O_2 \xrightarrow{k_3} \text{products} \quad \text{(slow step)}$$

For **Mechanism 1b**, we assume that MOOH is relatively stable and exists in chemical equilibrium with MOH (Eq. S11).

$$K_2 = \frac{[MOOH][H_2O]}{[MOH][H_2O_2]} \quad \text{(S11)}$$

Using this assumption in combination with mass-balance equations we can find all POM form ($M(OH)_2M$, MOH, and MOOH) concentrations.

The reaction rate before simplification is:

$$W_o = \frac{k_3K_2K_1[H_2O_2]^2}{4}\left(\sqrt{\left(\frac{K_2[H_2O_2]}{[H_2O]}+1\right)^2+\frac{16[M(OH)_2M]_o}{K_1}}-\frac{K_2[H_2O_2]}{[H_2O]}-1\right) \quad \text{(S12)}$$

After simplification (by using the Taylor series):

$$W_0 = \frac{2k_3K_2[M(OH)_2M]_0[H_2O_2]^2}{K_2[H_2O_2]+[H_2O]} \quad \text{(S13)}$$

Hence, we can conclude that **Mechanism 1b** also gives second reaction order in H_2O_2 when $[H_2O_2]$ is small and first reaction order in H_2O_2 when $[H_2O_2]$ is high.

Mechanism 2a

$$M(OH)_2M \underset{}{\overset{K_1}{\rightleftharpoons}} 2MOH \quad (1)$$

$$MOH + H_2O_2 \overset{K_2}{\rightleftharpoons} MOOH + H_2O \quad (2)$$

$$MOOH + H_2O_2 \xrightarrow{k_3} \text{products} \quad (3)$$

$$MOOH + H_2O_2 \underset{k_{-4}}{\overset{k_4}{\rightleftharpoons}} MOOH(H_2O_2) \quad (4)$$

$$MOOH(H_2O_2) \xrightarrow{k_5} \text{products} \quad (5)$$

For **Mechanism 2a**, we assume existence of a diperoxo complex $MOOH(H_2O_2)$ along with existence of monoperoxo MOOH. While MOOH is relatively stable and exists in chemical equilibrium with MOH, $MOOH(H_2O_2)$ is not stable and decomposes due to reverse transformation to MOOH and/or formation of peroxide degradation products.

$$K_2 = \frac{[MOOH][H_2O]}{[MOH][H_2O_2]} \quad \text{(S14)}$$

$$\frac{d[MOOH(H_2O_2)]}{dt} = k_4[MOOH][H_2O_2] - (k_{-4} + k_5)[MOOH(H_2O_2)] = 0 \quad \text{(S15)}$$

$$[MOOH(H_2O_2)] = \frac{k_4}{k_{-4}+k_5}[MOOH][H_2O_2] = k_{eff}[MOOH][H_2O_2]\,, \quad \text{(S16)}$$

where k_{eff} <<1 due instability of $MOOH(H_2O_2)$.

For **Mechanism 2a**, the rate of H_2O_2 decomposition has two contributions (Eq. S17): MOOH decomposition (step 3, W_{MOOH}) and $MOOH(H_2O_2)$ decomposition (step 5, $W_{MOOH(H2O2)}$):

$$W_0 = W_{MOOH} + W_{MOOH(H2O2)} \quad \text{(S17)}$$

Step 3 contribution to the rate of reaction:

$$W_{MOOH} = \frac{2k_3K_2[M(OH)_2M]_0[H_2O_2]^2}{k_{eff}K_2[H_2O_2]^2+K_2[H_2O_2]+[H_2O]} \approx \frac{2k_3K_2[M(OH)_2M]_0[H_2O_2]^2}{K_2[H_2O_2]+[H_2O]} \quad \text{(S18)}$$

Step 5 contribution to the rate of reaction:

$$W_{MOOH(H2O2)} = \frac{2k_5k_{eff}K_2[M(OH)_2M]_0[H_2O_2]^2}{k_{eff}K_2[H_2O_2]^2+K_2[H_2O_2]+[H_2O]} \approx \frac{2k_5k_{eff}K_2[M(OH)_2M]_0[H_2O_2]^2}{K_2[H_2O_2]+[H_2O]} \quad \text{(S19)}$$

Mechanism 2a gives second reaction order in H_2O_2 when $[H_2O_2]$ is small and first reaction order when $[H_2O_2]$ is high.

Mechanism 2b

$$M(OH)_2M \underset{}{\overset{K_1}{\rightleftharpoons}} 2\,MOH \qquad (1)$$

$$MOH + H_2O_2 \overset{K_2}{\rightleftharpoons} MOOH + H_2O \qquad (2)$$

$$MOOH + H_2O_2 \xrightarrow{k_3} \text{products} \qquad (3)$$

$$MOOH + H_2O_2 \overset{K_4}{\rightleftharpoons} MOOH(H_2O_2) \qquad (4)$$

$$MOOH(H_2O_2) \xrightarrow{k_5} \text{products} \qquad (5)$$

For **Mechanism 2b**, we assume that MOOH and $MOOH(H_2O_2)$ are relatively stable and exist in chemical equilibriums with MOH and MOOH, respectively.

$$K_2 = \frac{[MOOH][H_2O]}{[MOH][H_2O_2]} \qquad (S20)$$

$$K_4 = \frac{[MOOH(H_2O_2)]}{[MOOH][H_2O_2]} \qquad (S21)$$

The rate of H_2O_2 decomposition has two contributions: MOOH decomposition (step 3, W_{MOOH}) and $MOOH(H_2O_2)$ decomposition (step 5, $W_{MOOH(H2O2)}$):

$$W_0 = W_{MOOH} + W_{MOOH(H2O2)} \qquad (S22)$$

Step 3 contribution to the reaction rate:

$$W_{MOOH} = \frac{2k_3K_2[M(OH)_2M]_o[H_2O_2]^2}{K_4K_2[H_2O_2]^2+K_2[H_2O_2]+[H_2O]} \qquad (S23)$$

Step 5 contribution to the reaction rate:

$$W_{MOOH(H2O2)} = \frac{2k_5K_2K_4[M(OH)_2M]_o[H_2O_2]^2}{K_4K_2[H_2O_2]^2+K_2[H_2O_2]+[H_2O]} \qquad (S24)$$

The reaction rate is therefore:

$$W_0 = \frac{2(k_5K_4+k_3)K_2[M(OH)_2M]_o[H_2O_2]^2}{K_4K_2[H_2O_2]^2+K_2[H_2O_2]+[H_2O]} \qquad (S24.1)$$

Mechanism 2b gives first reaction order in H_2O_2 when $[H_2O_2]$ is small and zero reaction order when $[H_2O_2]$ is high.

Mechanism 3a

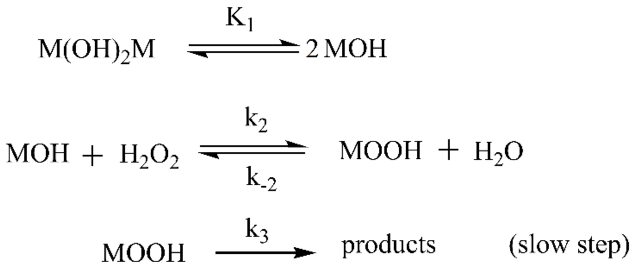

For **Mechanism 3a**, we assume that MOOH is not stable and prone to reverse transformation to MOH and/or decomposes to give peroxide degradation products.

$$\frac{d[MOOH]}{dt} = k_2[MOH][H_2O_2] - k_{-2}[MOOH][H_2O] - k_3[MOOH] = 0 \quad \text{(S25)}$$

Using this approximation in combination with mass-balance we can find all POM form ($M(OH)_2M$, MOH, and MOOH) concentrations.

The reaction rate after simplification (by using the Taylor series):

$$W_o = \frac{2k_3k_2[M(OH)_2M]_o[H_2O_2]}{k_2[H_2O_2]+k_{-2}[H_2O]+k_3} \quad \text{(S26)}$$

Therefore, **Mechanism 3a** gives first reaction order in H_2O_2 when $[H_2O_2]$ is small and zero reaction order when $[H_2O_2]$ is high.

Mechanism 3b

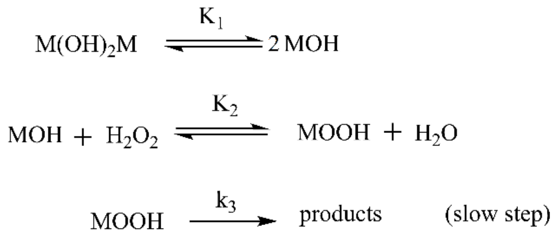

For **Mechanism 3b**, we assume that MOOH is relatively stable and exists in chemical equilibrium with MOH.

$$K_2 = \frac{[MOOH][H_2O]}{[MOH][H_2O_2]} \quad \text{(S27)}$$

Using that approximation in combination with mass-balance allows us to find all POM form ($M(OH)_2M$, MOH, and MOOH) concentrations.

The rate of reaction after simplification (by using Taylor series):

$$W_o = \frac{2k_3K_2[M(OH)_2M]_o[H_2O_2]}{K_2[H_2O_2]+[H_2O]} \quad \text{(S28)}$$

Mechanism 3b gives first reaction order in H_2O_2 when $[H_2O_2]$ is small and zero reaction order when $[H_2O_2]$ is high.

Mechanism 4

$$M(OH)_2M + H_2O_2 \overset{K_1}{\rightleftharpoons} M(O_2)(H_2O)M + H_2O \quad (1)$$

$$M(O_2)(H_2O)M + H_2O_2 \overset{K_2}{\rightleftharpoons} M(O_2)(H_2O_2)(H_2O)M + H_2O \quad (2)$$

$$M(O_2)(H_2O)M + H_2O_2 \xrightarrow{k_3} \text{products} \quad (3)$$

$$M(O_2)(H_2O_2)(H_2O)M \xrightarrow{k_4} \text{products} \quad (4)$$

For **Mechanism 4**, we assume that the dimeric form of POM, $\{ZrW_5\}_2$ ($M(OH)_2M$), is not hydrolyzed into the monomeric form MOH, but it reacts directly with H_2O_2 and exists in chemical equilibrium with mono- and diperoxo complexes ($\{\{ZrW_5\}_2(O_2)(H_2O)\}$ and $\{\{ZrW_5\}_2(O_2)(H_2O_2)(H_2O)\}$, respectively).

$$K_1 = \frac{[MOM(O_2)(H_2O)][H_2O]}{[M(OH)_2M][H_2O_2]} \quad (S29)$$

$$K_2 = \frac{[MOM(O_2)(H_2O_2)(H_2O)]}{[MOM(O_2)(H_2O)][H_2O_2]} \quad (S30)$$

These approximations in combination with the mass-balance equation allow us to find all POM form ($M(OH)_2M$, $\{M(O_2)(H_2O)M\}$ and $\{M(O_2)(H_2O_2)(H_2O)M\}$) concentrations.

$$[M(OH)_2M] + [M(O_2)(H_2O)M] + [M(O_2)(H_2O_2)(H_2O)M] = [M(OH)_2]_0 \quad (S31)$$

$$[M(O_2)(H_2O)M] = \frac{K_1[M(OH)_2M]_0[H_2O_2]}{K_2K_1[H_2O_2]^2+K_1[H_2O_2]+[H_2O]} \quad (S32)$$

$$[M(O_2)(H_2O_2)(H_2O)M] = \frac{K_2K_1[M(OH)_2M]_0[H_2O_2]^2}{K_2K_1[H_2O_2]^2+K_1[H_2O_2]+[H_2O]} \quad (S33)$$

For **Mechanism 4**, the rate of H_2O_2 decomposition has two contributions: $\{M(O_2)(H_2O)M\}$ decomposition (step 3, $W_{MOM(O2)}$) and $\{M(O_2)(H_2O_2)(H_2O)M\}$ decomposition (step 4, $W_{MOM(O2)2}$):

$$W_0 = W_{MOM(O2)} + W_{MOM(O2)2}$$

$$W_{MOM(O2)} = \frac{k_3K_1[M(OH)_2M]_0[H_2O_2]^2}{K_2K_1[H_2O_2]^2+K_1[H_2O_2]+[H_2O]} \quad (S34)$$

$$W_{MOM(O2)2} = \frac{k_4K_2K_1[M(OH)_2M]_0[H_2O_2]^2}{K_2K_1[H_2O_2]^2+K_1[H_2O_2]+[H_2O]} \quad (S35)$$

The reaction rate is therefore:

$$W_0 = \frac{(k_3+k_4K_2)K_1[M(OH)_2M]_0[H_2O_2]^2}{K_2K_1[H_2O_2]^2+K_1[H_2O_2]+[H_2O]} \quad (S35.1)$$

Mechanisms **2b**, **3a**, **3b**, and **4** give 1-0 reaction order in [H_2O_2] and first reaction order in [POM]. Thus, based only on the observed reaction orders in the reactants, we cannot distinguish between

these mechanisms. In order to choose the most probable mechanism, we have used double reverse coordinates $1/W_0$ -$1/[H_2O_2]$.

Mechanisms **2b** and **4** have the same expression for the reaction rate (compare Eqs. S24.1 and S35.1):

$$W_0 = \frac{ABC[H_2O_2]^2}{BC[H_2O_2]^2+C[H_2O_2]+[H_2O]}, \quad \text{(S36)}$$

where A, B, and C depend on the specific mechanism (**2b** or **4**) and $[M(OH)_2M]_0$.

Therefore, for mechanisms **2b** and **4,** the reaction rate function becomes polynomial in double reverse coordinates (Eq. 37):

$$\frac{1}{W_0} = \frac{[H_2O]}{ABC}\left(\frac{1}{[H_2O_2]}\right)^2 + \frac{1}{AB}\left(\frac{1}{[H_2O_2]}\right) + \frac{1}{A} \quad \text{(S37)}$$

On the other hand, mechanisms **3a** and **3b** have another expression for the reaction rate (see Eqs. S26 and S28):

$$W_0 = \frac{AB[H_2O_2]}{B[H_2O_2]+[H_2O]}, \quad \text{(S38)}$$

where A, B, and C depend on the mechanism type (**3a** or **3b**) and $[M(OH)_2M]_0$.

Therefore, for mechanisms **3a** and **3b**, the reaction rate function becomes linear in the double reverse coordinates (Eq. 39):

$$\frac{1}{W_0} = \frac{[H_2O]}{AB}\left(\frac{1}{[H_2O_2]}\right) + \frac{1}{A} \quad \text{(S39)}$$

The kinetic modelling study made also possible to estimate the value of K_4 (mechanism **2b**) as ca. 20. On the other hand, K_2 was impossible to determine because its value is determined by the position of the minimum of the polynomial, which we fit to the experimental data in inverse coordinates (Figure S6). To accurately determine the minimum, it is necessary to have experimental data points in both branches of the polynomial. However, at positive K_2, the minimum is in the negative region along the $1/[H_2O_2]$ axis; therefore, it is impossible to obtain experimental points for the second branch of the polynomial, since they correspond to negative H_2O_2 concentrations. Therefore, the value of K_2 cannot be determined with a good accuracy within the framework of our model.

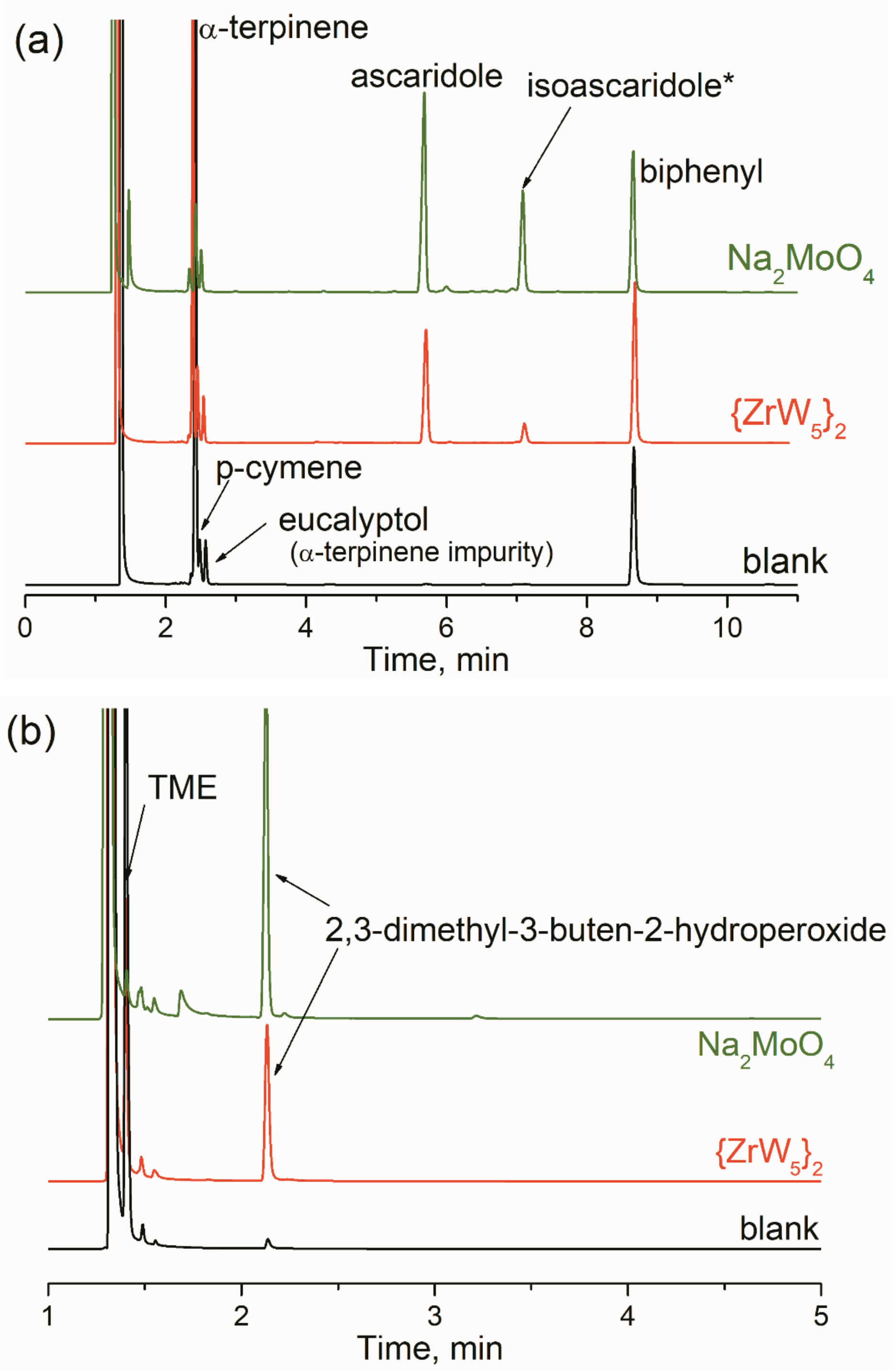

Figure S1. Oxidation of (a) α-terpinene and (b) tetramethylethylene with H_2O_2 (35% in water). Chromatograms of the reaction mixtures after the oxidation over **{ZrW5}$_2$** in comparison with a blank reaction and oxidation in the presence of Na_2MoO_4. Reaction conditions: for blank experiment, 0.1 *M* α-terpinene, 0.1 *M* H_2O_2 1 mL CH_3CN, 27 °C; for **{ZrW5}$_2$**, 0.1 *M* α-terpinene or TME, 0.004 *M* Zr, 0.1 *M* H_2O_2, 1 mL CH_3CN, 27 °C; for Na_2MoO_4 0.1 *M* α-terpinene or TME, 0.02 *M* Na_2MoO_4, 0.36 *M* H_2O_2 (35%), 1 mL EtOH, 40 °C.

* Isoascaridol was formed in injector from ascaridole during GC analysis, as confirmed in independent experiments.

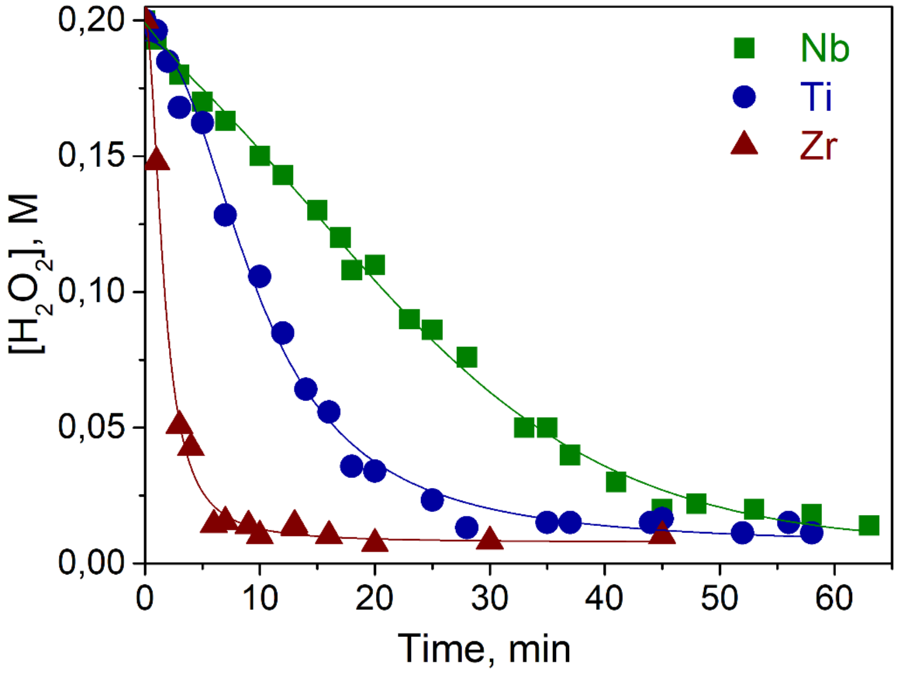

Figure S2. H_2O_2 decomposition in the presence of **NbW$_5$**, **TiW$_5$**, and **{ZrW$_5$}$_2$**. Reaction conditions: 0.008 *M* Nb, Ti or Zr, 0.2 *M* H_2O_2 (30%), 3 mL CH_3CN, 70 °C.

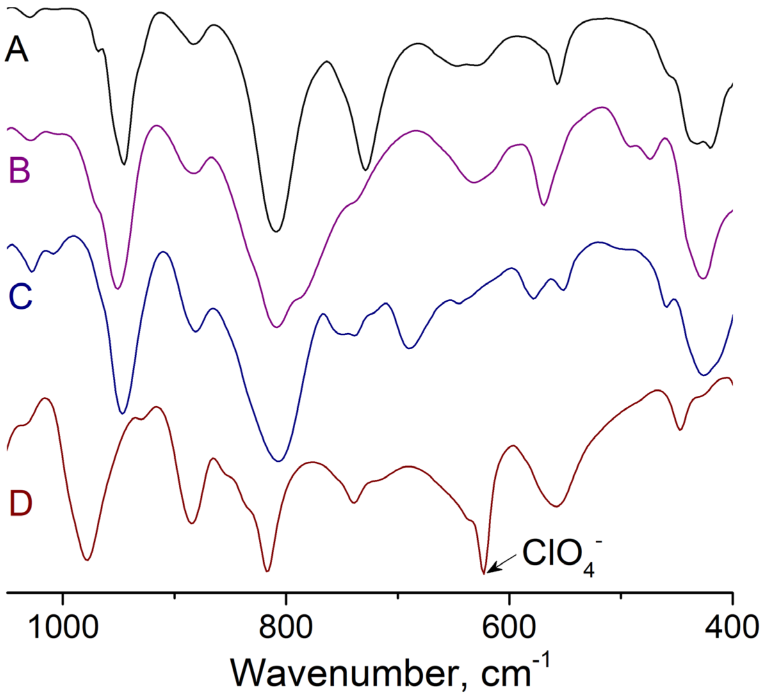

Figure S3. FT-IR spectra (in KBr pellets) of **{ZrW$_5$}$_2$**: (A) initial dimer (B) after H_2O_2 decomposition in the presence of **{ZrW$_5$}$_2$**, (C) after H_2O_2 decomposition in the presence of **{ZrW$_5$}$_2$** and 1 equiv. of Bu_4NOH, (D) after H_2O_2 decomposition in the presence of **{ZrW$_5$}$_2$** and 1 equiv. of $HClO_4$ (0.008 *M* Zr, 0.008 *M* $HClO_4$ or Bu_4NOH (if added), 0.2 *M* H_2O_2 (30%), 3 mL CH_3CN, 50 °C).

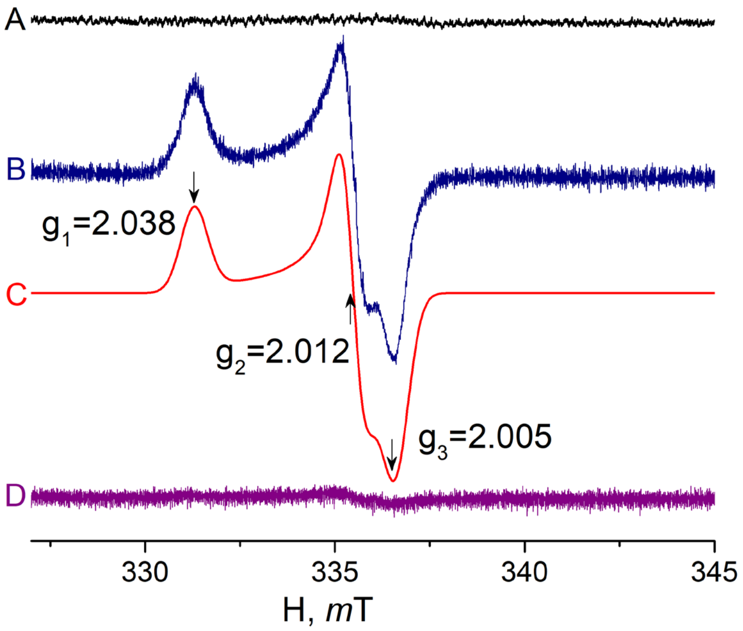

Figure S4. Frozen solution (−196 °C) EPR spectra: (A) - 0.06 M solution of **$\{ZrW_5\}_2$** in CH_3CN; (B) - sample in (A) immediately after addition of 77% H_2O_2 (2 equiv. per Zr); (C) - simulated spectrum for sample in (B); (D) - sample in (B) after 25 min storing at room temperature.

The following parameters were used for EPR spectrum simulation (C): g_1 = 2.0375; g_2 = 2.0121; g_3 = 2.0053; g_1Strain = 0.0051; g_2Strain = 0.0041; g_3Strain = 0.0049.

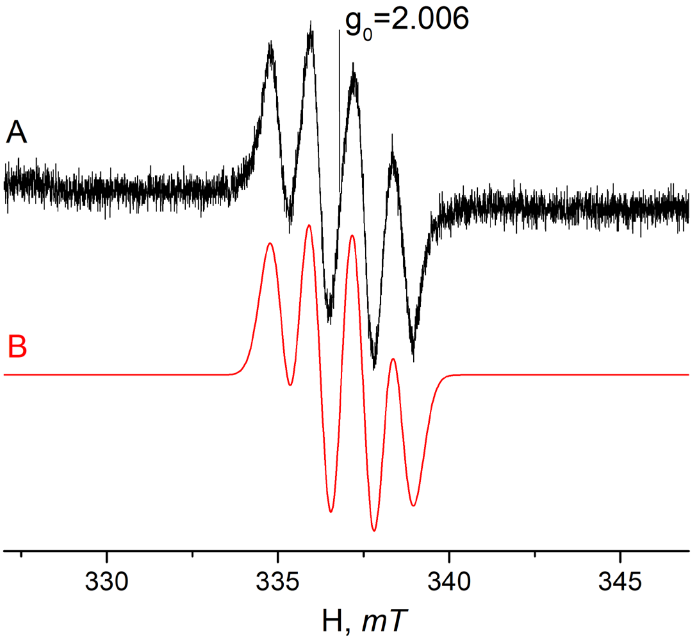

Figure S5. Experimental (A) and simulated (B) EPR spectra of the DMPO–radical adduct formed upon interaction of **$\{ZrW_5\}_2$** with H_2O_2 (0.004 *M* Zr, 0.2 M H_2O_2, 0.054 M DMPO, 100 μL CH_3CN, 25 °C). The following parameters were used for EPR spectrum simulation: g_0 = 2.0056; $A(^{14}N)$ = 1.28 mT, $A(^{1}H^{\beta})$ = 1.03 mT; $A(^{1}H^{\gamma})$ = 0.13 mT.

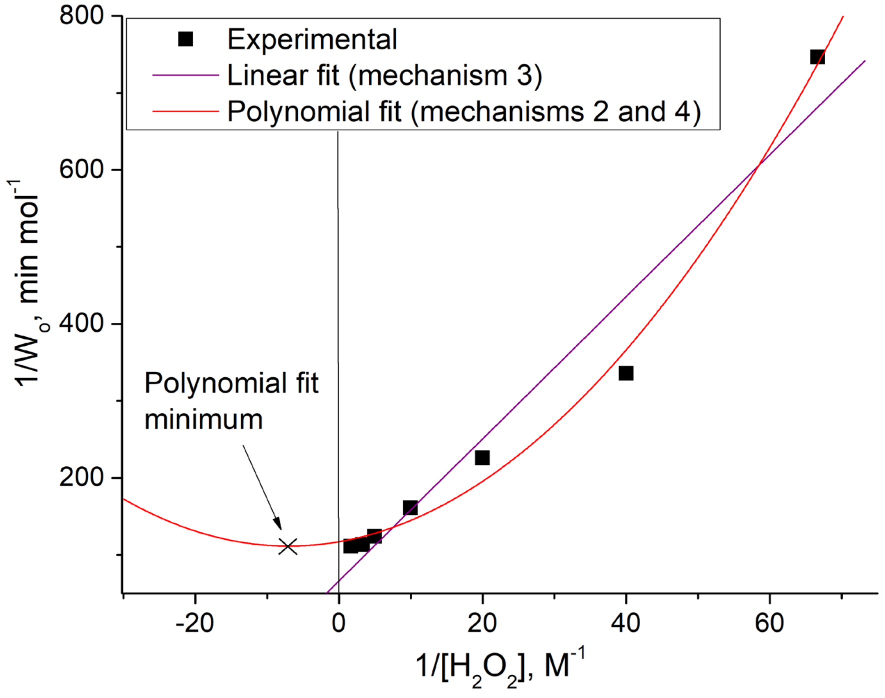

Figure S6. Experimental plot of the initial rate of H_2O_2 decomposition in the presence of **{ZrW$_5$}$_2$** at 50 °C in double reverse coordinates (1/W$_0$ – 1/[H_2O_2]) with fitted linear (Eq. S39) and polynomial (Eq. S37) plots.

Optimization of α-terpinene oxidation

The optimization of reaction conditions was carried out for the α-terpinene oxidation in order to obtain a maximum yield of ascaridole. Double increase of the catalyst amount (H_2O_2/Zr = 25 vs 50) led to the increase of α-terpinene conversion and product yield as well as reduced the reaction time (Table S1, compare entries 1 and 2). Note that at such molar ratio of H_2O_2 to Zr epoxidation of cyclohexene was negligible.[¡Error! Marcador no definido.] When a more concentrated H_2O_2 reagent (50 or 77% instead of 30%) was used, α-terpinene conversion and ascaridole yield increased (Table S1, entries 3 and 4). This indicates that less amount of H_2O in the reaction medium is more favorable for the formation of the endoperoxide. However, α-terpinene oxidation with anhydrous urea-hydrogen peroxide adduct was not successful: the ascaridole yield reached only 25% (Table S1, entry 5). Further optimization of the reaction conditions was performed using 50% H_2O_2 as more accessible and safer oxidant.

Table S1. α-Terpinene oxidation with H_2O_2 over **{ZrW$_5$}$_2$**.[a]

Entry	[Zr], M	Time, h	α-Terpinene conversion, %	Selectivity, %	
				Ascaridole	p-Cymene
1 [b]	0.004	5	50	80	8
2 [b]	0.008	2	65	82	11
3 [c]	0.008	2	70	89	9
4 [d]	0.008	2	75	91	7
5 [e]	0.008	1.5	39	64	12
6 [c,f]	0.008	5	63	89	5
7 [c,g]	0.008	4	97	10	16

[a] Reaction conditions: 0.1 *M* α-terpinene, 0.2 *M* H_2O_2, 1 mL CH_3CN, 27 °C. [b] 30% H_2O_2. [c] 50% H_2O_2. [d] 77% H_2O_2. [e] Urea-H_2O_2. [f] with 0.008 *M* Bu_4NOH (methanol solution). [g] with 0.008 *M* $HClO_4$.

The concentration of H_2O_2 in the reaction mixture turned out to be a crucial factor for the α-terpinene oxidation (Figure S7). Once the oxidant was added in one portion, the stoichiometric amount of H_2O_2 (i.e., 2 equiv. to substrate) was optimal. With 1 equiv. of oxidant, the yield of ascaridole reached only 32%, while the increase of H_2O_2 concentration up to 3 equiv. also reduced the yield down to 43% in comparison with 62% for 2 equiv. of H_2O_2. However, when 3 equiv. of the oxidant was added dropwise into the reaction mixture, endoperoxide yield increased significantly and attained 88% (Figure S7). In all cases the selectivity to ascaridole was 84-88%. Under the same conditions, with the dropwise addition of the oxidant, TME produced the corresponding hydroperoxide (Scheme 2, main text) with a 70% yield.

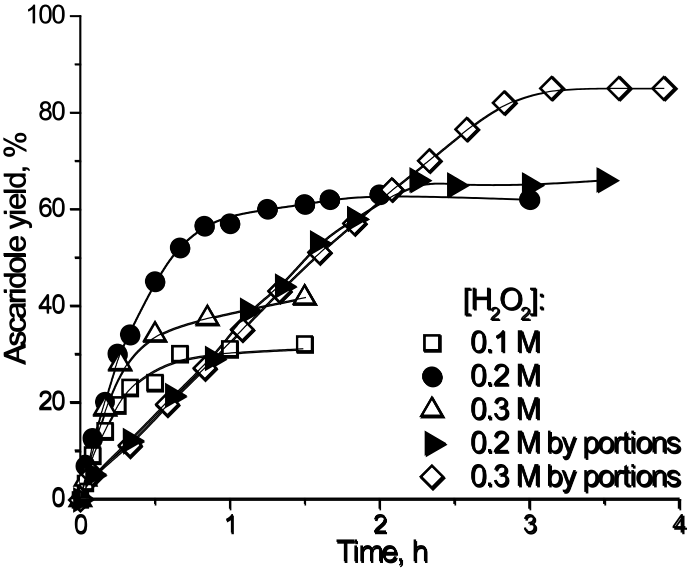

Figure S7. The effect of H_2O_2 (50%) concentration on the ascaridole yield in α-terpinene oxidation in the presence of {**ZrW**$_5$}$_2$. Reaction conditions: 0.1 *M* α-terpinene, 0.008 *M* Zr, 1 mL CH_3CN, 27 °C, H_2O_2 was added by portion of 0.05 mmol every 30 min, if mentioned.

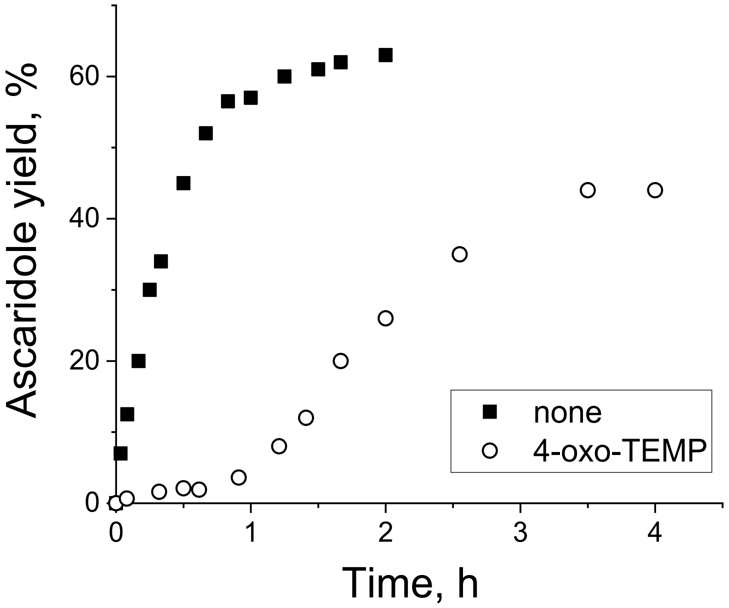

Figure S8. The effect of 4-oxo-TEMP on the rate of ascaridole formation in the α-terpinene oxidation with {**ZrW**$_5$}$_2$. Reaction conditions: 0.1 *M* α-terpinene, 0.008 *M* Zr, 0.2 *M* H_2O_2 (50%), 0.01 *M* 4-oxo-TEMP (if added), 1 mL CH_3CN, 27 °C.

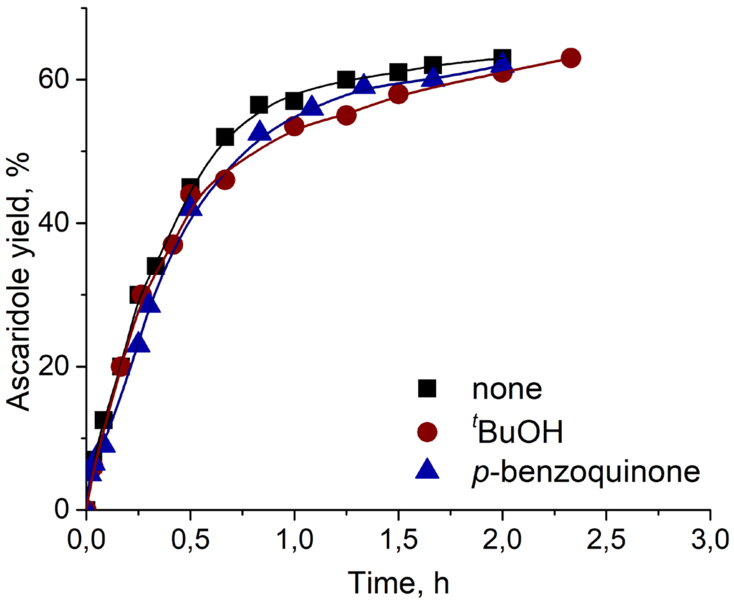

Figure S9. The influence of ^{t}BuOH and *p*-benzoquinone on the rate of ascaridole formation in the α-terpinene oxidation in the presence of **{ZrW**$_5$**}**$_2$. Reaction conditions: 0.1 *M* α-terpinene, 0.008 *M* Zr, 0.2 *M* H_2O_2 (50%), 0.0008 *M* ^{t}BuOH or *p*-benzoquinone (if added), 1 mL CH_3CN, 27 °C.

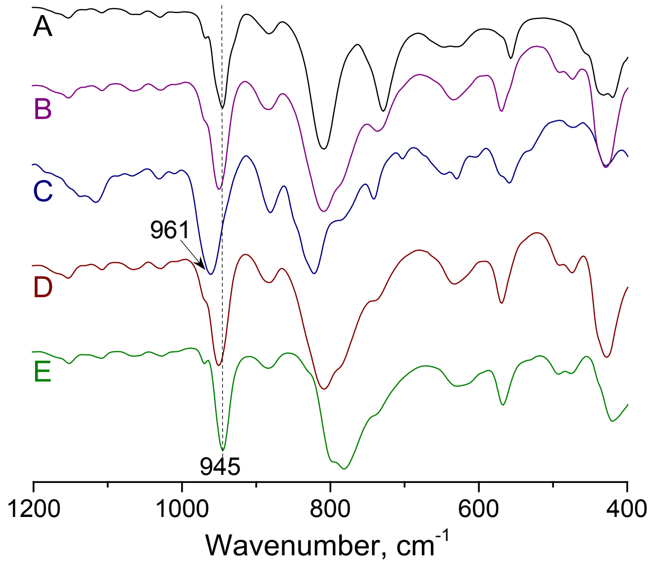

Figure S10. FT-IR spectra (in KBr pellets) of **{ZrW**$_5$**}**$_2$: (A) initial dimer, (B) after α-terpinene oxidation with 0.2 M 50% H_2O_2, (C) after α-terpinene oxidation with 0.3 M 30% H_2O_2, (D) after α-terpinene oxidation with 0.3 M 50% H_2O_2 added dropwise, and (E) ATR-FT-IR spectrum of $(Bu_4N)_6[(\mu\text{-}\eta^2\text{:}\eta^2\text{-}O_2)\{ZrW_5O_{18}\}_2]$ (taken from [Maksimchuk N.V., et al. *ACS Catal.* **2021**, *11*, 10589−10603]). Reaction conditions for (B)-(D): 0.1 *M* α-terpinene, 0.008 *M* Zr, 1 mL CH_3CN, 27 °C.

Additional computational data

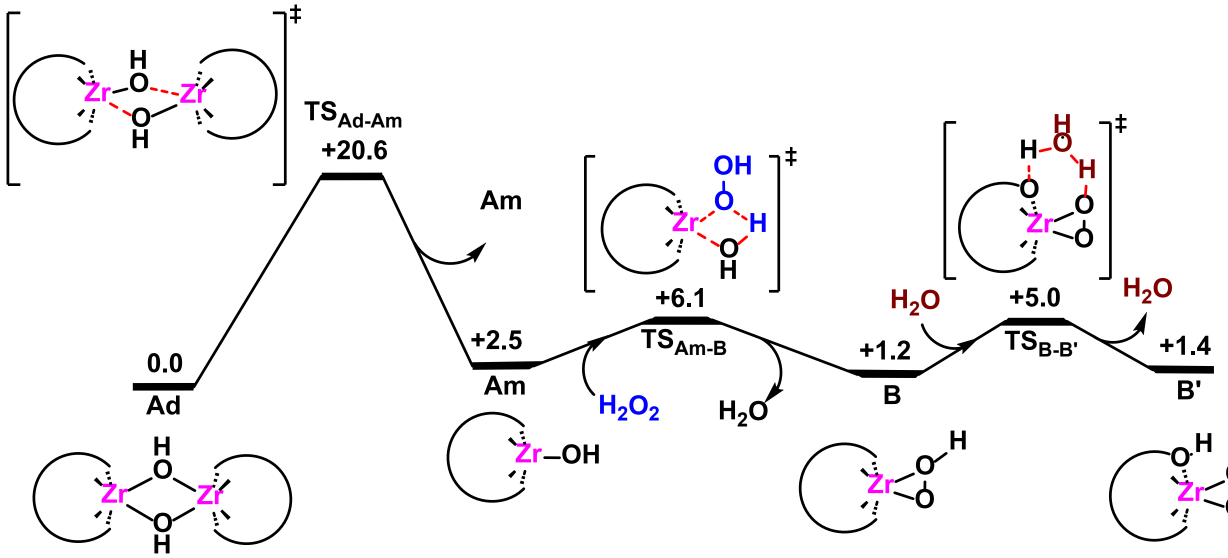

Figure S11. Gibbs free-energy path of the monomerization and its 1st H_2O_2 activation. All free-energies are in kcal·mol^{-1} and are relative to the starting dimeric structure **Ad**.

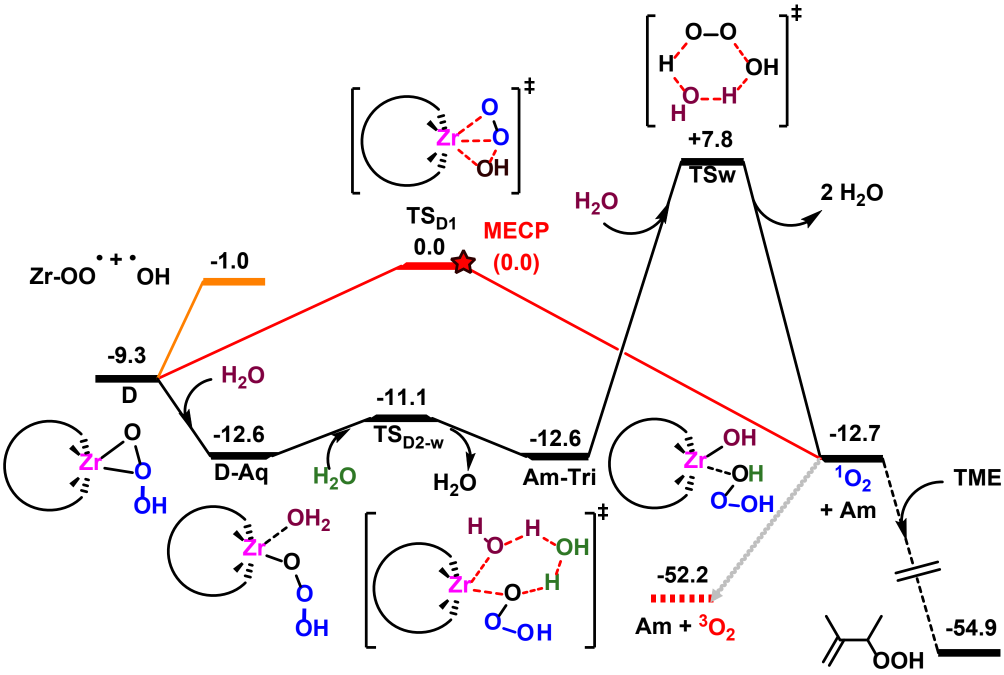

Figure S12. Free-energy profile (kcal·mol^{-1}) for the evolution of monomeric Zr-trioxide intermediate **D** to produce singlet oxygen through a water mediated process where trioxidane is formed. All energies are relative to the initial dimeric specie **Ad**.

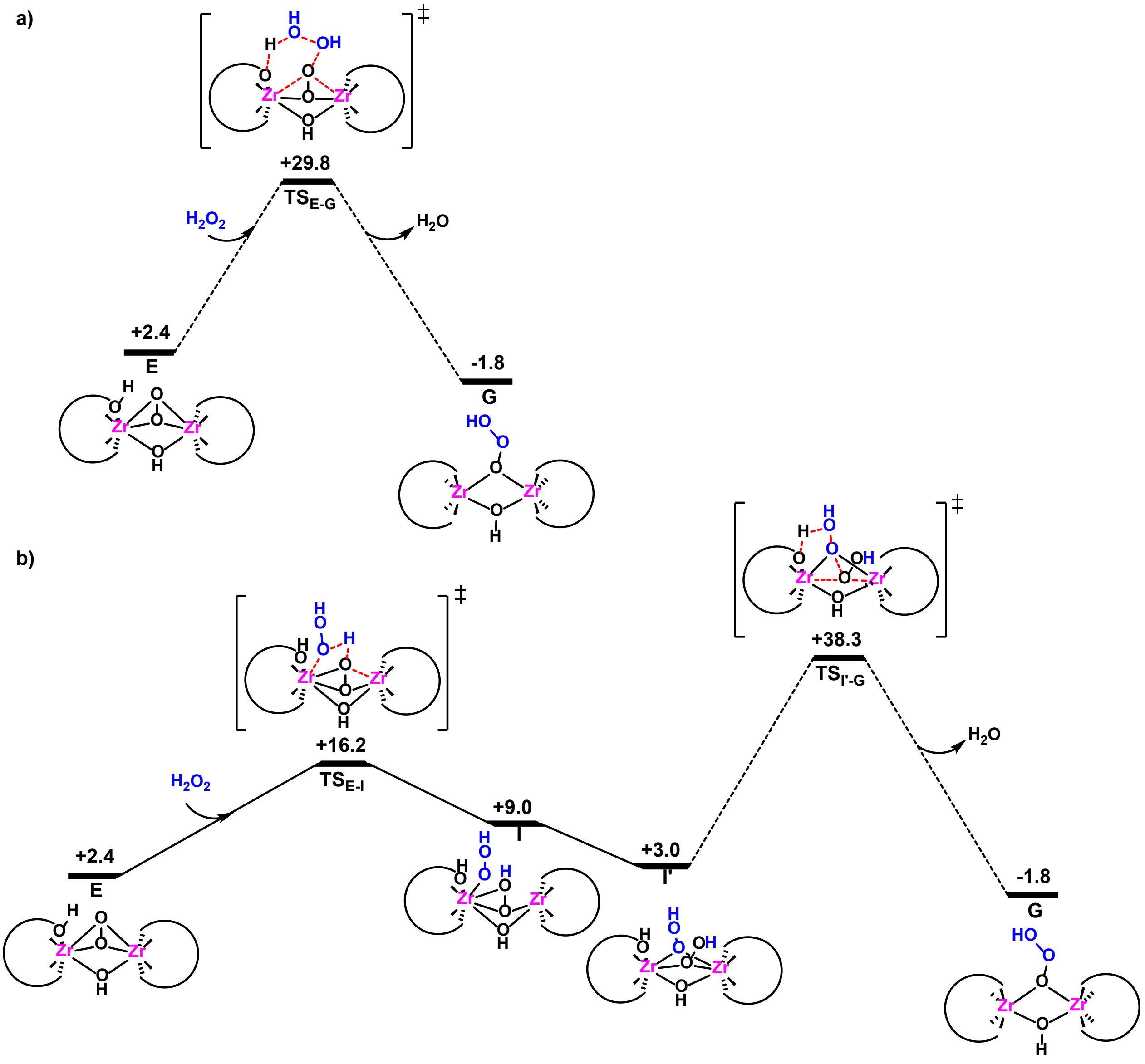

Figure S13. Alternative reaction pathways for the second H_2O_2 activation by Zr-dimeric species. Gibbs free energies with respect to species **Ad** are given in kcal·mol^{-1}.

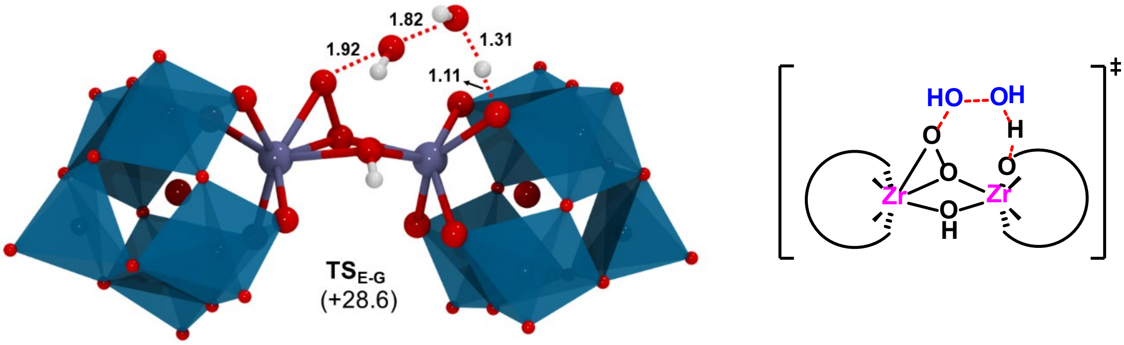

Figure S14. Representation of the **TS$_{E-G}$** involved in the outer-sphere H_2O_2 activation forming the dimeric trioxidane intermediate **G**. Selected distances in Å and free-energy (kcal·mol^{-1}) is relative to the starting dimeric structure **Ad**.

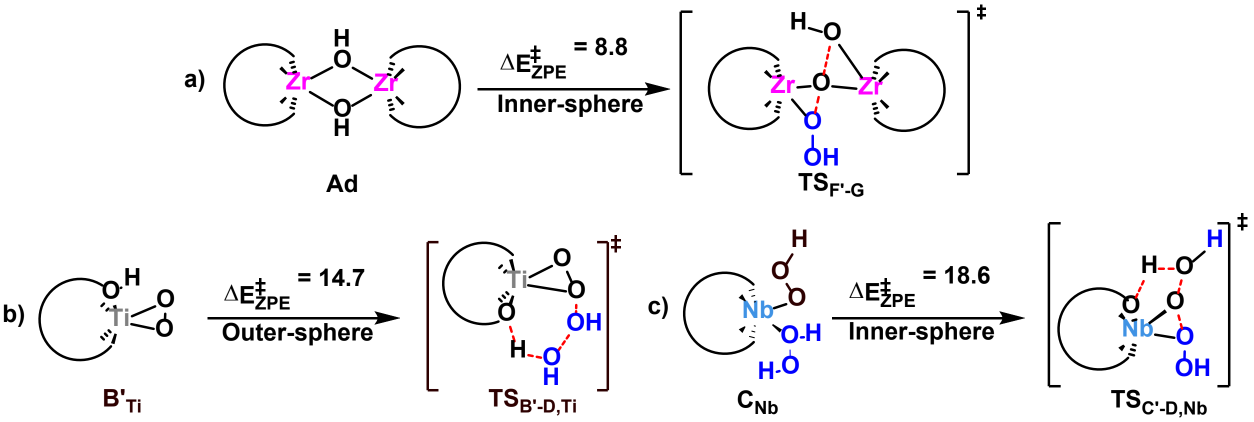

Figure S15. Calculated Zero Point-corrected electronic energy barriers ($\Delta E^{\ddagger}_{ZPE}$) for H_2O_2 decomposition by Zr-, Ti-, and Nb-substituted Lindqvist POMs (a, b and c respectively) to yield TM-trioxidane intermediate. All energies are given in kcal·mol^{-1}.

Table S2. Comparison of the experimental activation energies (E_a) energies and calculated Zero Point-corrected electronic energy barriers ($\Delta E^{\ddagger}_{ZPE}$) for the rate-determining step of H_2O_2 decomposition reaction. All energies are given in kcal·mol^{-1}.

Transition Metal	E_a (exp.)	$\Delta E^{\ddagger}_{ZPE}$	Path
Zr^{IV}	11.5	**9.2** [a]	Inner
Ti^{IV}	14.6	**14.7** [b]	Outer
Nb^{V}	16.7	**18.6** [c]	Inner

[a] Average of the three accessible pathways [i) **Ad** to **TS**$_{\mathbf{F'\text{-}G}}$; ii) **C** to **TS**$_{\mathbf{C'\text{-}D}}$; and iii) **C** to **TS**$_{\mathbf{B'\text{-}D}}$] weighed according to a Boltzmann distribution (see main text). [b] From **B'**$_{\mathbf{Ti}}$ to **TS**$_{\mathbf{B'\text{-}D,Ti}}$. [c] From **C**$_{\mathbf{Nb}}$ to **TS**$_{\mathbf{C'\text{-}D,Nb}}$.

Table S3. Distribution of reaction paths for H_2O_2 decomposition by **Ad** at 50 °C derived from microkinetic modelling. Rate constants for elementary steps were obtained from DFT-calculated free-energy barriers and by means of the Eyring equation and initial concentrations of $\{ZrW_5\}_2$, H_2O_2 and H_2O (0.08 M, 0.20 M and 0.66 M, respectively) were set to reproduce the experimental conditions. Entries 1-3 and 4-6 evaluate the impact of the water content on the reaction rate and overall path distribution. Reaction rates ($r_{rel.}$) were estimated as the time needed to consume 90% of the initial H_2O_2 and were normalized by the fastest one (Entry 3).

**Entry**	$[H_2O]$ (M)	mechanism **1** (%) (Monomeric Outer)	mechanism **2** (%) (Monomeric Inner)	mechanism **4** (%) (Dimeric)	$r_{rel.}$ (M s^{-1})
**1**	0.66	< 0.1	39.0	61.0	0.10
**2**	0.20	< 0.1	13.5	86.5	0.26
**3**	0.00	< 0.1	2.4	97.6	1.00
**4**[a]	0.66	< 0.1	2.9	97.1	0.03
**5**[a]	0.20	< 0.1	0.9	99.1	0.15
**6**[a]	0.00	< 0.1	1.4	98.6	0.75

[a] Energy correction of +2 kcal·mol^{-1} applied to the monomeric transition state involved in the trioxidane intermediate **D** formation.

Cartesian coordinates (Å) of the most representative optimized structures.

Dataset collection freely accessible online: https://doi.org/10.19061/iochem-bd-2-62

52

Ad G(ha): -3634.615365

W	1.99273	14.06439	4.75820
W	2.29631	14.71293	1.47730
W	2.48876	11.56880	2.57082
W	4.73627	15.58201	3.56273
W	4.93064	12.43875	4.65328
Zr	5.28354	13.07691	1.31658
O	0.82395	14.41581	5.98166
O	1.24627	15.56628	0.40117
O	1.58586	10.12234	2.28537
O	5.48455	17.07450	4.01015
O	5.81418	11.63374	5.90244
O	5.63558	12.49300	-0.77168
O	1.27420	14.85814	3.16876
O	1.42870	12.38219	4.02834
O	3.20669	15.54949	4.81361
O	3.35827	13.07679	5.67318
O	3.71832	14.19934	0.40203
O	1.61529	12.88900	1.44017
O	3.40385	16.11309	2.24552
O	3.89196	11.46818	1.36109
O	3.71575	11.07069	3.99820
O	5.80444	14.94050	2.18382
O	5.52972	14.28879	4.77767
O	5.98160	12.21504	3.14275
O	3.65916	13.56295	3.01630
Zr	7.74817	12.35346	-1.30486
O	7.38614	12.90400	0.78277
O	9.51593	11.59334	-0.34818
W	10.96281	11.20382	-1.43056
O	12.17101	10.63974	-0.32982
O	11.94090	11.05400	-3.14751
W	11.05821	11.55731	-4.77205
O	12.23546	11.27526	-6.00500
O	11.36161	13.37348	-4.22824
W	10.22481	14.14551	-2.80924
O	10.88361	15.73842	-2.69819
O	11.33446	13.11070	-1.59566
O	10.06792	9.57813	-2.01644
W	8.63250	9.75760	-3.31649
O	8.12358	8.12792	-3.58336
O	10.10056	9.90016	-4.63268
O	9.52857	12.22007	-5.71704
W	7.89965	12.70263	-4.69885
O	6.86512	13.22386	-5.98224

O	8.89471	14.30221	-4.22361
O	8.85099	14.14063	-1.55418
O	7.50572	10.36016	-1.95690
O	7.60837	10.77502	-4.61461
O	6.86599	12.90766	-3.17850
O	9.38204	11.96194	-3.01389
H	8.08656	12.79917	1.43789
H	4.97605	12.63217	-1.46203

58

TS_Ad-Am G(ha): -3787.424150

O	4.27617	0.26364	0.25809
W	4.83896	0.14082	2.54591
W	4.63921	2.58922	0.30597
W	3.86347	0.37774	-2.06656
W	4.05014	-2.07411	0.16491
Zr	1.95572	0.54445	0.79964
W	6.66775	-0.02200	-0.26776
O	5.06935	2.02466	2.11428
O	4.24216	2.21391	-1.56522
O	3.79033	-1.52720	-1.68153
O	4.53925	-1.70816	2.01226
O	2.83133	2.47848	0.70491
O	2.29465	-1.54252	0.52157
O	5.96413	-1.81111	-0.21948
O	6.43525	1.87220	-0.13144
O	2.14422	0.54769	-1.35391
O	3.01226	0.41976	2.65998
O	6.59400	-0.07362	1.64069
O	5.81262	0.12726	-1.98619
O	5.34057	0.02582	4.19561
O	3.60830	0.45349	-3.77237
O	8.34139	-0.22708	-0.64165
O	4.96747	4.28542	0.32153
O	3.94901	-3.79676	0.09410
O	-4.33130	-0.21369	-0.22074
W	-3.86951	0.03441	2.05956
W	-3.85758	2.05760	-0.57014
W	-4.81739	-0.47128	-2.49872
W	-4.85962	-2.48098	0.13012
Zr	-1.91006	-0.82509	-0.66487
W	-6.62894	0.35434	0.19357
O	-3.61636	1.82334	1.34994
O	-4.39379	1.41927	-2.32734
O	-5.23679	-2.21659	-1.76246
O	-4.43070	-1.82178	1.90940
O	-2.17964	1.31060	-0.84120
O	-3.06278	-2.58321	-0.26900
O	-6.58963	-1.54887	0.40355
O	-5.78334	2.04965	-0.15650
O	-3.01459	-0.90318	-2.49544

O	-2.17646	-0.44895	1.42224
O	-5.79259	0.44383	1.93054
O	-6.56285	0.05954	-1.69372
O	-3.61354	0.24026	3.75673
O	-5.27746	-0.62347	-4.15940
O	-8.27963	0.77267	0.50255
O	-3.59680	3.75125	-0.80532
O	-5.32917	-4.12388	0.40467
O	0.11132	1.20606	0.46888
O	-0.35329	-2.04118	-0.36429
H	-0.55528	1.61975	-0.10660
H	0.58981	-1.94572	-0.13838
O	0.45343	-0.53396	2.44187
H	0.55608	-0.08150	3.29027
H	-0.47600	-0.38692	2.14164
O	-0.29741	-0.06961	-2.36283
H	-0.12060	-0.83065	-2.93374
H	0.59199	0.17807	-1.99235
26			
Am	G(ha): -1817.307190		
O	-0.16545	0.01659	-0.00000
W	-0.36933	-2.32804	0.00000
W	2.22973	-0.22201	0.00000
W	0.10110	2.35822	-0.00000
W	-0.13928	0.01424	2.35520
W	-0.13928	0.01424	-2.35520
O	-2.16316	-1.81125	0.00000
O	1.58192	-2.03000	-0.00000
O	1.94925	1.68325	0.00000
O	-1.76428	2.20350	0.00000
O	-0.26213	-1.86916	1.88611
O	0.10704	1.88525	1.88560
O	0.10704	1.88525	-1.88560
O	-0.26213	-1.86916	-1.88611
O	1.76362	-0.17272	1.86275
O	-1.96692	0.19350	2.02598
O	-1.96692	0.19350	-2.02598
O	1.76362	-0.17272	-1.86275
O	-4.50060	0.75237	-0.00000
O	3.94821	-0.39239	-0.00000
O	-0.02943	0.00397	4.07815
O	-0.02943	0.00397	-4.07815
O	-0.43402	-4.05321	-0.00000
O	0.37179	4.06312	-0.00000
H	-5.28893	0.19660	-0.00000
Zr	-2.55757	0.26424	-0.00000
33			
TS_Am-B	G(ha): -2045.267592		
O	-0.02818	-0.00452	-0.29614
W	-1.62664	0.47523	-1.95464

W	1.60793	-0.30963	-1.97282
W	1.53591	-0.63689	1.34382
W	-1.70601	0.11793	1.36171
W	-0.60268	-2.40231	-0.55481
Zr	0.52783	2.29338	-0.06106
O	-2.64784	0.49230	-0.30083
O	-0.00689	0.09739	-2.96844
O	-0.12447	-0.41144	2.34528
O	2.52720	-0.77699	-0.31828
O	1.73264	1.21104	1.30541
O	0.74185	-2.36693	0.81104
O	1.80999	1.48584	-1.56960
O	0.81044	-2.12215	-1.80439
O	-0.95866	2.16437	-1.56247
O	-1.74614	-1.49390	-1.79305
O	-1.04251	1.87785	1.32383
O	-1.80689	-1.75441	0.81241
O	2.66697	-1.15370	2.54268
O	-2.81254	0.76974	-3.17516
O	2.78995	-0.59918	-3.19850
O	-2.94631	0.18135	2.56037
O	-1.01067	-4.07042	-0.72322
O	-0.37325	4.32494	0.33057
O	2.13440	3.78266	0.09678
H	3.01149	3.38625	0.17655
H	1.91429	4.71282	0.86861
O	-1.72785	4.35910	0.85769
H	-1.77539	3.43624	1.21287
H	0.34439	5.04732	1.02640
O	1.32193	5.54112	1.48300
H	1.36719	5.30567	2.42104
27			
B	G(ha): -1892.430677		
O	0.00911	0.06075	-0.30110
W	-1.54315	0.59874	-1.99446
W	1.69047	-0.22117	-1.94671
W	1.53212	-0.61970	1.36529
W	-1.70497	0.21025	1.32048
W	-0.58790	-2.30399	-0.61241
Zr	0.57145	2.29654	0.00407
O	-2.60199	0.59635	-0.36924
O	0.10318	0.23379	-2.96880
O	-0.14854	-0.37079	2.32383
O	2.56052	-0.72720	-0.27382
O	1.76054	1.23978	1.36309
O	0.73574	-2.32078	0.78358
O	1.90304	1.56373	-1.49237
O	0.86640	-2.01704	-1.82405
O	-0.85967	2.27924	-1.54398
O	-1.68240	-1.36575	-1.87942

O	-1.04968	1.95419	1.32875
O	-1.80659	-1.67087	0.72796
O	2.62762	-1.16852	2.58057
O	-2.69415	0.93646	-3.23557
O	2.89854	-0.49846	-3.14877
O	-2.97530	0.24209	2.48930
O	-1.00663	-3.96394	-0.82723
O	1.60086	4.11412	-0.15743
O	0.95441	4.28716	1.16442
H	0.28072	4.96923	0.99389

25

Zr_oxyl_rad G(ha): -1816.623947

O	0.02850	0.06529	-0.29598
W	-1.52518	0.63690	-1.97873
W	1.70143	-0.21504	-1.92518
W	1.54043	-0.62306	1.38019
W	-1.68446	0.23033	1.32730
W	-0.59017	-2.27954	-0.61139
Zr	0.62348	2.32582	0.00742
O	-2.58553	0.63062	-0.34510
O	0.11964	0.27682	-2.94842
O	-0.13118	-0.37335	2.33555
O	2.56974	-0.73731	-0.27219
O	1.79576	1.22252	1.37381
O	0.73547	-2.31990	0.77546
O	1.93820	1.58497	-1.47103
O	0.85917	-2.00067	-1.83489
O	-0.86296	2.31197	-1.49615
O	-1.68303	-1.32601	-1.86952
O	-0.97136	1.95693	1.32903
O	-1.80348	-1.64324	0.73987
O	2.63290	-1.19285	2.58899
O	-2.67269	0.98569	-3.22010
O	2.90506	-0.49464	-3.13020
O	-2.94509	0.29793	2.50384
O	-1.03041	-3.93330	-0.83122
O	0.66711	4.15763	0.99093

27

TS_B-B' G(ha): -1892.409135

O	0.03884	0.07168	-0.30050
W	-1.52109	0.62424	-1.98401
W	1.69757	-0.26808	-1.97504
W	1.56120	-0.65690	1.33868
W	-1.68598	0.15022	1.32328
W	-0.59662	-2.32901	-0.64330
Zr	0.69784	2.30850	-0.05035
O	-2.56021	0.64539	-0.32484
O	0.10489	0.20180	-2.97309
O	-0.11481	-0.37789	2.31215
O	2.55724	-0.82119	-0.32803

O	1.86841	1.16076	1.33626
O	0.69397	-2.34843	0.76002
O	1.98572	1.51844	-1.54885
O	0.82474	-2.06819	-1.86030
O	-0.83087	2.27905	-1.56575
O	-1.71273	-1.34686	-1.83775
O	-0.96859	1.96544	1.39482
O	-1.82462	-1.66313	0.74883
O	2.64729	-1.26581	2.53407
O	-2.68665	0.95434	-3.21379
O	2.87464	-0.59377	-3.19177
O	-2.93911	0.22635	2.50016
O	-1.06293	-3.97566	-0.84781
O	1.00197	3.93992	1.16254
O	-0.36370	4.09981	0.59203
H	-0.99700	3.07204	1.21386

30

B_Adduct G(ha): -1968.858227

O	0.02992	0.20142	-0.38267
W	-1.43164	0.65596	-2.17677
W	1.73735	-0.36266	-1.91678
W	1.45689	-0.37363	1.40881
W	-1.71576	0.62640	1.14732
W	-0.70598	-2.13624	-0.45781
Zr	0.75658	2.43821	-0.32433
O	-2.53801	0.89291	-0.59199
O	0.21969	0.09349	-3.04108
O	-0.23344	0.07130	2.27023
O	2.52355	-0.73248	-0.17872
O	1.81565	1.43936	1.21913
O	0.56738	-2.08532	0.97242
O	2.06062	1.45873	-1.64976
O	0.79373	-2.07547	-1.65038
O	-0.68240	2.33341	-1.87309
O	-1.70290	-1.27189	-1.85023
O	-0.92147	2.32536	0.99390
O	-1.92668	-1.28608	0.76703
O	2.47409	-0.86437	2.71428
O	-2.52130	0.91605	-3.48981
O	2.95999	-0.83803	-3.03780
O	-3.00845	0.88571	2.25943
O	-1.23005	-3.77914	-0.50965
O	1.32674	4.39776	-0.79073
O	1.35270	4.40176	0.69024
H	0.48376	4.83773	0.95394
O	-1.09053	5.00991	1.47240
H	-1.08633	5.21585	2.41751
H	-1.27750	4.04341	1.41920

30

TS_B-B'w G(ha): -1235498.8105489

O	-0.00926	0.09528	-0.46854
W	-1.43851	0.40055	-2.31765
W	1.78065	-0.39983	-1.95104
W	1.40993	-0.34104	1.36428
W	-1.83128	0.35230	0.99574
W	-0.55851	-2.32469	-0.52899
Zr	0.58778	2.40091	-0.46952
O	-2.60167	0.59867	-0.75751
O	0.26590	-0.09956	-3.12194
O	-0.33892	-0.00024	2.17107
O	2.53139	-0.69711	-0.18631
O	1.67231	1.46674	1.15937
O	0.62499	-2.13048	0.94817
O	1.98861	1.43500	-1.72484
O	0.94894	-2.18946	-1.67166
O	-0.81748	2.11774	-2.08011
O	-1.59751	-1.54382	-1.92618
O	-1.13757	2.16992	0.82320
O	-1.90493	-1.52839	0.66952
O	2.41248	-0.75839	2.70741
O	-2.51683	0.52542	-3.66078
O	3.05907	-0.82800	-3.02657
O	-3.14730	0.58997	2.08072
O	-0.97322	-3.99867	-0.54874
O	0.26481	4.41492	-0.71268
O	0.97122	4.24514	0.58743
H	0.00067	4.33054	1.48100
H	-1.15500	3.11592	1.58207
O	-0.91002	4.15972	2.12339
H	-0.60609	3.97664	3.02458
27			
B'	G(ha): -1892.430274		
O	0.03303	0.05851	-0.25717
W	-1.46272	0.70980	-1.91654
W	1.73782	-0.22067	-1.94697
W	1.55722	-0.64023	1.35131
W	-1.70897	0.07841	1.35112
W	-0.57515	-2.28930	-0.66663
Zr	0.75331	2.37950	0.04425
O	-2.52875	0.71777	-0.26427
O	0.13645	0.26593	-2.92526
O	-0.13829	-0.33253	2.33798
O	2.56458	-0.82043	-0.28145
O	1.86794	1.18488	1.40087
O	0.65698	-2.32282	0.78962
O	2.04733	1.51802	-1.49937
O	0.84407	-2.04362	-1.85417
O	-0.75472	2.35613	-1.46008
O	-1.70508	-1.26690	-1.80768
O	-0.97069	2.02947	1.46811

O	-1.87164	-1.68053	0.76554
O	2.61017	-1.26785	2.56380
O	-2.62613	1.08183	-3.13381
O	2.91147	-0.56005	-3.16273
O	-2.95549	0.22047	2.52899
O	-1.05676	-3.92772	-0.89597
O	1.73484	4.11780	-0.27902
O	0.68131	4.26606	0.77359
H	-1.66019	2.69988	1.35680

31

C G(ha): -2043.972956

Zr	2.10142	0.68633	1.25200
O	2.70889	0.49707	-0.77975
O	0.55106	0.52278	2.68150
O	1.09003	2.43810	0.56631
O	2.18074	-1.41827	1.30640
W	0.78557	-2.31826	0.44699
O	-0.38761	-1.86806	1.92756
O	1.61379	-1.90583	-1.26412
O	-0.87896	-2.45892	-0.59729
O	1.15502	-3.98514	0.69650
W	-1.76414	-0.85660	-1.17320
O	-0.38995	-0.71384	-2.49713
O	-2.35819	-0.67773	0.63961
O	-1.87895	1.06172	-1.26317
O	-3.14973	-1.41233	-2.03694
W	-0.48316	2.14897	-0.41187
O	0.59909	1.66409	-1.94604
O	-1.39264	1.70411	1.25047
O	-1.03709	3.74142	-0.77699
W	1.41851	-0.10436	-1.96921
O	2.23825	-0.15905	-3.48744
O	0.22828	-0.05363	0.07065
W	-1.09256	-0.05427	2.02428
O	-2.11059	-0.07564	3.41817
O	4.20700	0.80253	1.68116
O	3.68175	0.25818	2.95226
H	3.84473	-0.69837	2.84589
O	2.51144	2.79774	2.68473
H	2.03330	3.29337	1.98451
O	3.88339	3.27352	2.55090
H	4.28949	2.42895	2.21297

34

TS_C-C' G(ha): -2120.384649

O	3.32304	2.70085	2.10374
Zr	2.43079	1.28373	0.75869
O	2.81017	0.64677	-1.25618
O	0.97461	1.34962	2.35023
O	1.25179	2.79355	-0.16347
O	2.51303	-0.80655	1.31448

W	1.06111	-1.90403	0.75232
O	0.03906	-1.15741	2.22458
O	1.76296	-1.83596	-1.05221
O	-0.65466	-2.28015	-0.06041
O	1.52393	-3.46339	1.32294
W	-1.64654	-0.82582	-0.89264
O	-0.39692	-0.98785	-2.32149
O	-2.06845	-0.30010	0.90539
O	-1.83495	1.00638	-1.36102
O	-3.08509	-1.57972	-1.47409
W	-0.37576	2.27800	-0.88636
O	0.54066	1.47577	-2.39592
O	-1.13597	2.17261	0.90111
O	-1.02717	3.74193	-1.52819
W	1.43384	-0.23158	-2.11530
O	2.09238	-0.61877	-3.66412
O	0.43402	0.22524	-0.03187
W	-0.70743	0.62291	1.99200
O	-1.58823	0.89218	3.45153
O	4.59366	1.05389	0.65033
O	5.27514	1.21873	1.95198
H	3.65600	-1.01950	2.10695
O	2.57090	3.24675	3.22394
H	1.79040	2.64368	3.20228
H	4.64426	1.92945	2.33860
O	4.55985	-0.97569	2.67428
H	4.97412	0.06873	2.43596
H	4.33105	-1.03625	3.61413
31			
C'	G(ha): -2043.968982		
O	2.87652	3.31117	2.73595
Zr	2.45985	1.61068	1.53746
O	2.90893	1.18714	-0.53662
O	0.80902	1.49398	3.03355
O	1.24693	3.17940	0.62501
O	2.44018	-0.56245	1.77007
W	1.08256	-1.53499	1.01599
O	-0.09547	-0.94384	2.49660
O	1.84231	-1.30421	-0.75039
O	-0.64058	-1.82798	0.06044
O	1.46117	-3.16766	1.42755
W	-1.52255	-0.35507	-0.76277
O	-0.20172	-0.31731	-2.08420
O	-2.19773	-0.00795	1.10471
O	-1.73288	1.53389	-0.93775
O	-2.90318	-1.03651	-1.53774
W	-0.31706	2.74228	-0.25176
O	0.70646	2.12689	-1.77584
O	-1.17809	2.44960	1.49279
O	-0.94612	4.26678	-0.75982

W	1.62865	0.42829	-1.60562
O	2.40851	0.23383	-3.13101
O	0.41011	0.62178	0.42865
W	-0.98427	0.72748	2.32282
O	-1.91699	0.87653	3.76226
O	4.51653	1.37491	1.78258
H	4.06736	-0.06335	2.97118
O	4.15439	0.89785	3.12791
O	1.64862	4.03320	3.08996
H	1.28526	4.18570	2.18883
H	0.78299	2.43496	3.33177

31

TS_B'-D G(ha): -2043.938437

O	2.68053	2.65337	2.44924
Zr	2.16658	0.76143	1.06009
O	2.64198	0.44502	-1.01524
O	0.62215	0.62402	2.56203
O	1.05843	2.47276	0.30466
O	2.20323	-1.35377	1.21073
W	0.80238	-2.30458	0.46791
O	-0.34329	-1.79964	1.94429
O	1.51809	-1.98263	-1.31081
O	-0.92813	-2.54136	-0.51333
O	1.19673	-3.95204	0.78706
W	-1.84581	-1.00676	-1.11534
O	-0.55111	-0.84250	-2.50341
O	-2.36240	-0.66315	0.68130
O	-2.03071	0.96518	-1.33044
O	-3.26126	-1.61648	-1.88667
W	-0.66126	2.05819	-0.62662
O	0.45179	1.56025	-2.10737
O	-1.37961	1.74730	1.13647
O	-1.19773	3.64921	-1.00477
W	1.29438	-0.21686	-2.08149
O	2.02024	-0.34386	-3.64250
O	0.18046	-0.05327	-0.02215
W	-1.02117	0.02493	1.99570
O	-1.99771	0.06614	3.41849
O	4.24556	0.91143	1.32910
O	3.65004	0.96576	2.62243
H	3.57177	3.00710	2.28806
O	1.78089	4.20805	1.87376
H	1.25429	3.31688	0.93855
H	2.49543	4.66359	1.39576

31

TS_C'-D G(ha): -2043.942446

O	2.81605	2.63679	2.06519
Zr	1.92463	0.96690	1.07277
O	2.52357	0.63516	-0.92739
O	0.39561	0.77285	2.51509

O	0.75289	2.58585	0.26993
O	2.13404	-1.17741	1.32623
W	0.80816	-2.21324	0.53975
O	-0.40525	-1.73115	1.97505
O	1.59086	-1.87475	-1.21024
O	-0.85580	-2.55558	-0.48963
O	1.27473	-3.83172	0.90894
W	-1.84611	-1.07736	-1.16799
O	-0.49943	-0.91730	-2.52079
O	-2.45219	-0.77302	0.62037
O	-2.11278	0.83391	-1.42188
O	-3.19217	-1.79385	-1.97317
W	-0.81874	2.06937	-0.68511
O	0.32535	1.55911	-2.15524
O	-1.64895	1.70721	1.03230
O	-1.48831	3.58378	-1.16100
W	1.26218	-0.15451	-2.04679
O	2.07311	-0.27310	-3.56515
O	0.07030	-0.02833	-0.03127
W	-1.21382	0.03854	1.93539
O	-2.21167	0.06529	3.34261
O	3.91100	0.90681	1.89664
H	3.36515	-0.75240	2.64682
O	3.78515	0.03796	3.05766
O	1.78297	4.00502	1.94917
H	1.21195	3.58326	1.16430
H	2.41602	4.62176	1.54594
28			
D	G(ha): -1967.568713		
O	3.58362	2.60837	3.10179
Zr	1.98758	0.86870	0.90182
O	2.51769	0.51961	-1.11266
O	0.53008	0.75611	2.43398
O	0.86345	2.48768	0.15053
O	2.17299	-1.20597	1.16380
W	0.79989	-2.25816	0.45929
O	-0.32696	-1.73428	1.95293
O	1.52893	-1.96389	-1.32150
O	-0.89913	-2.57299	-0.48451
O	1.27260	-3.87289	0.84303
W	-1.89464	-1.06915	-1.14732
O	-0.59262	-0.98874	-2.55469
O	-2.41350	-0.76189	0.67090
O	-2.11185	0.82076	-1.40443
O	-3.28782	-1.76795	-1.88627
W	-0.72955	2.04653	-0.71775
O	0.29869	1.47463	-2.26282
O	-1.54691	1.70761	1.01627
O	-1.37953	3.57287	-1.19318
W	1.19876	-0.25100	-2.17942

O	1.94812	-0.40553	-3.72661
O	0.10048	-0.07203	-0.09489
W	-1.11548	0.04537	1.92595
O	-2.05846	0.10687	3.37032
O	4.04879	1.18034	1.28504
O	3.63243	1.22254	2.65989
H	4.52772	2.77536	3.27803

34

D_Adduct G(ha): -2120.418884

O	-0.10021	-0.01317	-0.23999
W	-1.86015	0.45790	-1.74160
W	1.40102	-0.11652	-2.04344
W	1.63141	-0.64747	1.23788
W	-1.62441	-0.04030	1.54697
W	-0.56633	-2.40888	-0.58191
Zr	0.35335	2.31422	0.10230
O	-2.72868	0.35450	-0.00994
O	-0.30614	0.26132	-2.89224
O	0.05179	-0.56970	2.37184
O	2.48195	-0.63044	-0.50673
O	1.74077	1.20571	1.30588
O	0.88833	-2.38999	0.65746
O	1.53706	1.66391	-1.53466
O	0.71312	-1.97332	-1.93395
O	-1.24190	2.17823	-1.30284
O	-1.86015	-1.49764	-1.68859
O	-1.04147	1.73272	1.55551
O	-1.68528	-1.89842	0.89519
O	2.88327	-1.17492	2.30342
O	-3.15789	0.76415	-2.83646
O	2.49223	-0.25586	-3.37444
O	-2.75916	-0.10817	2.84625
O	-0.90649	-4.08316	-0.82338
O	-0.25891	4.37292	0.29653
O	2.24369	3.65859	0.48301
H	2.79178	3.07008	1.02590
H	2.10954	4.51760	0.98013
O	-1.68629	4.55206	0.21820
H	0.61311	5.56831	1.20669
O	1.44972	5.89999	1.61890
H	1.32463	5.77578	2.57002
O	-2.03433	4.69788	-1.16553
H	-1.91892	3.75296	-1.44752

26

Zr_OO_rad G(ha): -1891.818572

O	0.02075	0.10310	-0.30838
W	-1.55250	0.60492	-1.99525
W	1.68358	-0.22818	-1.96317
W	1.54537	-0.60047	1.35382
W	-1.68568	0.25520	1.32329

W	-0.60014	-2.29378	-0.59497
Zr	0.60011	2.31372	-0.03703
O	-2.59885	0.61709	-0.35862
O	0.08922	0.23466	-2.97391
O	-0.12373	-0.33432	2.31979
O	2.56201	-0.72333	-0.29512
O	1.78022	1.25784	1.32448
O	0.74010	-2.29866	0.78499
O	1.92151	1.56218	-1.52921
O	0.84497	-2.01390	-1.82003
O	-0.88069	2.28876	-1.55239
O	-1.69491	-1.35683	-1.86035
O	-1.01244	1.98868	1.31165
O	-1.79511	-1.63611	0.74817
O	2.64931	-1.14054	2.56481
O	-2.71043	0.93152	-3.23225
O	2.87752	-0.52732	-3.17333
O	-2.94568	0.30307	2.50212
O	-1.02740	-3.95268	-0.79215
O	1.96584	4.06698	0.15146
O	1.06583	4.16067	1.13298

28

TS_D1 G(ha): -1967.553904

O	-4.08455	1.71330	0.86833
Zr	-2.34704	0.41551	0.44956
O	-1.69138	-0.96130	1.89374
O	-1.88452	1.59065	-1.20872
O	-1.28405	1.84031	1.59982
O	-2.26677	-1.21647	-0.89669
W	-0.57098	-1.76033	-1.42797
O	-0.47971	-0.20997	-2.60898
O	-0.30615	-2.60372	0.30003
O	1.40348	-1.67150	-1.48220
O	-0.86819	-3.08311	-2.49620
W	2.31747	-0.34865	-0.44245
O	1.93390	-1.45109	1.07980
O	1.76185	0.91356	-1.78272
O	2.29327	1.13315	0.77633
O	3.98995	-0.61907	-0.76490
W	0.56695	1.81708	1.44162
O	0.60300	0.24536	2.59644
O	0.41289	2.64079	-0.31273
O	1.09231	3.10295	2.46646
W	0.10723	-1.46170	1.82339
O	0.28371	-2.57223	3.13217
O	-0.08423	0.04344	0.01907
W	-0.10919	1.51910	-1.81498
O	-0.08822	2.59634	-3.16234
O	-4.73931	-0.84074	0.82794
O	-4.64409	0.10661	-0.02096

H	-4.62387	1.59449	1.66718

31

D-Aq G(ha): -2043.993848

O	-0.11201	-0.03425	-0.24455
W	-1.86750	0.43916	-1.74788
W	1.39794	-0.12507	-2.04471
W	1.61773	-0.64882	1.24051
W	-1.64433	-0.06403	1.54075
W	-0.56295	-2.42224	-0.58657
Zr	0.33629	2.28959	0.09905
O	-2.74438	0.32217	-0.02089
O	-0.31047	0.25202	-2.89462
O	0.03575	-0.58431	2.36850
O	2.47575	-0.62425	-0.50091
O	1.71597	1.20916	1.31120
O	0.89170	-2.39602	0.65495
O	1.52208	1.65791	-1.53789
O	0.72103	-1.98117	-1.93731
O	-1.26632	2.16475	-1.29831
O	-1.85591	-1.51839	-1.69898
O	-1.07188	1.70767	1.55284
O	-1.68819	-1.92476	0.88739
O	2.86930	-1.16706	2.31060
O	-3.16292	0.73689	-2.84766
O	2.49406	-0.25855	-3.37230
O	-2.78356	-0.14331	2.83562
O	-0.89135	-4.09867	-0.83119
O	-0.14267	4.34061	0.35097
O	2.24814	3.69905	0.41345
H	2.78038	3.24963	1.08929
H	1.94266	4.54146	0.78815
O	-1.55995	4.58761	0.29029
O	-1.92653	4.74425	-1.09087
H	-1.87526	3.79575	-1.37565

31

TSD2 G(ha): -2043.980759

O	-0.11937	-0.09754	-0.17490
W	-1.88992	0.53355	-1.60407
W	1.36281	-0.06938	-2.00672
W	1.61364	-0.90566	1.21432
W	-1.63569	-0.28654	1.62039
W	-0.63428	-2.45549	-0.74048
Zr	0.38332	2.12605	0.37543
O	-2.74415	0.26615	0.11938
O	-0.35193	0.42653	-2.78358
O	0.04985	-0.91179	2.36525
O	2.44714	-0.73054	-0.53307
O	1.75110	0.94104	1.44831
O	0.84074	-2.56701	0.47352
O	1.52731	1.65831	-1.33207

O	0.64077	-1.90653	-2.06086
O	-1.25308	2.19469	-0.98995
O	-1.91876	-1.42077	-1.73841
O	-1.02365	1.46496	1.79160
O	-1.72148	-2.07421	0.79288
O	2.86760	-1.54651	2.21079
O	-3.18765	0.96102	-2.65553
O	2.43603	-0.09617	-3.35759
O	-2.75563	-0.46861	2.92020
O	-1.00044	-4.09295	-1.13642
O	-0.46365	4.30161	0.96508
O	1.75405	3.84073	0.71588
H	2.39551	3.82662	1.43852
H	0.71036	4.47109	0.87403
O	-1.31973	5.05791	0.08733
O	-0.91562	4.82852	-1.27143
H	-1.21970	3.89095	-1.39332

34

TS_D2W G(ha): -2120.410831

O	-0.09759	-0.05125	-0.19049
W	-1.82981	0.56399	-1.66707
W	1.41765	-0.06305	-1.98669
W	1.60137	-0.83377	1.25119
W	-1.64263	-0.17881	1.57664
W	-0.61710	-2.41068	-0.71194
Zr	0.41873	2.23572	0.33103
O	-2.72309	0.35030	0.03811
O	-0.26830	0.41419	-2.82113
O	0.00843	-0.81102	2.37410
O	2.47242	-0.70833	-0.47863
O	1.74207	1.00250	1.46302
O	0.82566	-2.51294	0.53626
O	1.58632	1.67299	-1.34571
O	0.68924	-1.90412	-2.01615
O	-1.17509	2.23014	-1.10090
O	-1.87409	-1.39105	-1.76464
O	-1.03160	1.57105	1.71938
O	-1.73982	-1.98550	0.78366
O	2.82981	-1.46788	2.28695
O	-3.10547	0.98268	-2.75158
O	2.52051	-0.13462	-3.31464
O	-2.79514	-0.32220	2.85513
O	-0.99059	-4.05499	-1.08024
O	-0.46609	4.29880	0.75218
O	1.98967	3.62650	0.84497
H	2.79292	3.21644	1.18831
H	1.83069	4.89586	0.90387
O	-1.79234	4.63162	0.32464
H	0.28731	5.29414	0.80333
O	1.28917	5.90001	0.86351

H	1.34914	6.32092	1.73366
O	-1.77557	4.77197	-1.10253
H	-1.67923	3.80964	-1.34302

31

D-Tri G(ha): -2043.985967

O	-0.07618	-0.13165	-0.17264
W	-1.79268	0.59881	-1.60897
W	1.43436	-0.12826	-1.97569
W	1.61270	-0.99572	1.23459
W	-1.60527	-0.22955	1.60876
W	-0.67324	-2.43094	-0.74614
Zr	0.54036	2.12609	0.41122
O	-2.68306	0.36405	0.09942
O	-0.24618	0.44427	-2.77284
O	0.04131	-0.93510	2.37213
O	2.47573	-0.85111	-0.50250
O	1.82931	0.84164	1.46454
O	0.78427	-2.62272	0.48229
O	1.67223	1.58434	-1.28343
O	0.63628	-1.93513	-2.05620
O	-1.09555	2.23153	-0.97626
O	-1.90788	-1.34695	-1.76290
O	-0.93899	1.49335	1.79878
O	-1.76229	-2.01828	0.77391
O	2.83056	-1.68655	2.24423
O	-3.05830	1.09403	-2.67124
O	2.52115	-0.19144	-3.31592
O	-2.74769	-0.37761	2.89501
O	-1.10217	-4.05109	-1.15755
O	-0.66467	4.39673	1.01572
O	1.72445	3.75342	0.79600
H	2.68076	3.63686	0.86682
H	0.13880	4.94686	0.88653
O	-1.66824	4.97661	0.14307
O	-1.22860	4.81353	-1.19712
H	-1.30978	3.81704	-1.29812

5

H2O3 G(ha): -226.683437

O	-1.65542	3.25380	1.16363
O	-1.36062	4.35928	0.30317
O	-2.17278	4.23272	-0.86894
H	-1.59343	3.71881	-1.46085
H	-0.99963	2.59295	0.87507

8

TS_w G(ha): -303.073915

O	-1.70607	2.86602	0.92146
O	-1.32342	4.44011	0.19613
O	-2.09205	4.59659	-0.85456
H	-1.77941	3.43805	-1.47724
O	-1.53936	2.35007	-1.42554

H	-2.28427	1.84760	-1.78936
H	-1.59815	2.35271	-0.30670
H	-0.84909	2.74639	1.36651

18

TME G(ha): -235.738504

C	-1.16899	1.07871	-0.65413
C	-0.49782	2.24914	-0.71708
C	-2.67590	0.96285	-0.56255
H	-3.20071	1.91797	-0.55146
H	-3.06619	0.37826	-1.40663
H	-2.96271	0.41463	0.34504
C	-0.51022	-0.28456	-0.66631
H	-0.77581	-0.84549	0.23999
H	-0.88024	-0.88035	-1.51168
H	0.57737	-0.25812	-0.73167
C	1.00906	2.36498	-0.80902
H	1.53377	1.40982	-0.82172
H	1.29547	2.91437	-1.71604
H	1.39983	2.94843	0.03563
C	-1.15653	3.61242	-0.70457
H	-0.89248	4.17296	-1.61156
H	-2.24399	3.58606	-0.63723
H	-0.78501	4.20856	0.13990

20

TMEO2 G(ha): -386.072776

C	0.70179	4.59840	-0.73883
C	1.38766	5.71512	-0.46987
H	0.90467	6.67660	-0.34445
H	2.46971	5.68855	-0.37246
C	1.40359	3.27115	-0.91278
H	2.46703	3.36909	-0.68104
H	0.98525	2.49694	-0.25897
H	1.31487	2.89806	-1.93938
C	-0.82638	4.58460	-0.85977
C	-1.30277	3.79367	-2.08700
H	-1.09071	2.72703	-1.97966
H	-2.38192	3.91741	-2.19934
H	-0.81160	4.15831	-2.99410
C	-1.45295	4.04442	0.43657
H	-1.16663	4.67421	1.28408
H	-2.54273	4.02499	0.35613
H	-1.11287	3.02491	0.63618
O	-1.18218	5.98368	-1.03574
O	-2.62861	6.11329	-1.08698
H	-2.81649	6.41876	-0.18380

53

E G(ha): -3709.732972

W	1.75965	14.00898	4.50802
W	3.39985	15.80290	2.19011
W	1.98640	12.90725	1.34785

W	5.10089	14.28567	4.60750
W	3.71468	11.38967	3.73884
Zr	5.43717	13.17635	1.35528
O	0.46144	14.31055	5.61258
O	3.18923	17.44732	1.69172
O	0.75365	12.42455	0.23127
O	6.14322	14.80593	5.88922
O	3.74445	9.78486	4.38743
O	5.81670	13.01441	-0.83082
O	1.99261	15.67365	3.57872
O	0.88171	13.39232	2.91459
O	3.33293	14.47265	5.49505
O	2.23628	12.18190	4.81490
O	4.80339	15.19800	1.13428
O	2.13189	14.83265	1.07899
O	4.61095	15.94091	3.70544
O	3.55723	12.66313	0.41686
O	2.36279	11.27744	2.34745
O	6.24716	13.87966	3.23129
O	4.86172	12.38111	4.95317
O	5.05077	11.37769	2.45514
O	3.59421	13.58892	2.93428
Zr	7.91085	12.41343	-0.98011
O	7.53720	13.69830	0.77007
O	9.88747	13.15748	-0.57918
W	11.23674	12.92313	-1.79603
O	12.61056	13.66386	-1.05795
O	12.03945	12.36973	-3.53469
W	11.00417	11.35043	-4.76643
O	12.06866	11.00801	-6.07876
O	10.27462	13.17084	-5.28264
W	9.06580	13.95154	-4.10091
O	8.66878	15.44448	-4.86112
O	10.45824	14.38722	-2.88424
O	11.39615	11.05665	-1.31205
W	10.01513	9.84181	-1.94366
O	10.49238	8.30814	-1.31846
O	11.13758	9.86643	-3.64057
O	9.29157	10.75940	-5.34351
W	7.73273	10.89756	-4.10250
O	6.52033	10.13088	-5.06333
O	7.67345	12.77090	-4.69862
O	7.87127	14.15388	-2.39277
O	8.82377	10.48543	-0.70228
O	8.59158	9.41497	-3.17482
O	6.85997	11.35273	-2.55902
O	9.45699	11.94528	-2.93645
H	5.16930	12.47887	-1.30883
O	7.47091	12.25694	1.12498
H	6.93058	14.31295	-2.56583

57
TS_E-F G(ha): -3861.255145

W	1.94649	13.52146	4.76695
W	3.26349	16.03919	3.06242
W	1.94990	13.50085	1.41972
W	5.26579	13.90615	4.55931
W	3.75823	11.33236	3.12221
Zr	5.45763	14.03176	1.00591
O	0.77812	13.31856	6.02666
O	2.95271	17.73428	3.14538
O	0.64754	13.30688	0.30046
O	6.45487	14.04340	5.80432
O	4.02540	9.63024	3.11693
O	5.70330	13.16852	-1.32837
O	1.97655	15.37887	4.46634
O	0.95971	13.27593	3.15005
O	3.62434	13.63439	5.67506
O	2.50058	11.58139	4.47099
O	4.52470	15.86908	1.73238
O	1.90665	15.38406	1.82159
O	4.59721	15.73659	4.40885
O	3.43852	13.68702	0.36315
O	2.47839	11.61460	1.75901
O	6.21630	14.14098	2.99678
O	5.17269	11.97876	4.26663
O	5.09569	11.86937	1.61225
O	3.51899	13.66226	3.08694
Zr	8.01426	12.62119	-1.29339
O	7.42980	14.27179	0.19720
O	9.93697	13.32950	-0.73333
W	11.40058	12.83906	-1.75236
O	12.73413	13.61994	-0.97566
O	12.32266	11.92449	-3.24069
W	11.28762	10.86805	-4.46363
O	12.44959	10.25532	-5.58762
O	10.71194	12.53116	-5.22484
W	9.35796	13.61280	-4.26126
O	9.20840	14.95173	-5.34544
O	10.85016	14.11234	-3.11946
O	11.35661	11.07387	-0.93174
W	9.98850	9.83248	-1.54010
O	10.30249	8.40353	-0.61806
O	11.21652	9.55106	-3.07713
O	9.61467	10.15226	-5.07127
W	7.96887	10.58885	-4.06772
O	6.79156	9.72374	-4.99383
O	8.09146	12.31412	-4.96485
O	8.17889	14.02365	-2.88454
O	8.71268	10.73833	-0.54821
O	8.61572	9.26561	-2.79459

O	6.98436	11.37177	-2.71170
O	9.62820	11.74782	-2.86091
H	5.14805	12.57537	-1.85253
O	7.40794	12.90781	0.77156
O	4.18300	16.19727	-0.95295
H	3.94589	16.33553	-0.01315
O	5.42099	15.44756	-0.76371
H	5.45063	14.20663	-1.42530
H	5.98782	11.54659	1.80949
54			
F	G(ha): -3784.849438		
W	2.30537	12.96418	4.92076
W	2.61764	15.61143	2.89721
W	2.37984	12.56852	1.57153
W	5.25015	14.58490	4.64354
W	4.89659	11.48022	3.46875
Zr	5.32148	14.25849	1.24675
O	1.25467	12.49742	6.20292
O	1.67532	17.05072	2.82210
O	1.24998	11.77679	0.53803
O	6.23059	15.28472	5.87755
O	5.73412	9.99690	3.69937
O	1.67292	14.65477	4.37954
O	1.53648	12.22639	3.33924
O	3.78079	13.82089	5.75927
O	3.55393	11.41313	4.78568
O	3.89175	15.83934	1.57135
O	1.65914	14.35548	1.76207
O	3.94584	15.97726	4.25059
O	3.68728	13.18744	0.41992
O	3.59151	11.11470	2.12499
O	6.14605	14.97918	3.08204
O	5.87510	12.73007	4.55909
O	5.95635	12.24809	1.92166
O	3.85668	13.57362	3.07053
Zr	8.68405	12.71654	-0.61398
O	7.48051	14.03176	0.62498
O	10.78721	12.82209	-0.31780
W	11.84506	12.09876	-1.66528
O	13.46114	12.34467	-1.10401
O	12.19508	11.20510	-3.39477
W	10.70629	10.68179	-4.48506
O	11.42954	9.95280	-5.87477
O	10.54341	12.53355	-4.95133
W	9.75743	13.79153	-3.63919
O	9.84976	15.26973	-4.52985
O	11.48958	13.66654	-2.76237
O	11.40415	10.31247	-1.03668
W	9.65231	9.59061	-1.48789
O	9.66867	8.00407	-0.80304

O	10.46702	9.22972	-3.25577
O	8.81735	10.55946	-4.81001
W	7.56654	11.28161	-3.46333
O	6.05235	10.93146	-4.21704
O	8.06013	13.01371	-4.19313
O	8.98912	14.28698	-2.02553
O	8.89172	10.64457	-0.16389
O	7.97971	9.65502	-2.47831
O	7.09109	12.10730	-1.86040
O	9.67953	11.71747	-2.51298
O	8.27647	13.06971	1.44063
O	4.50952	16.12185	-1.00831
H	4.03974	16.34280	-0.17455
O	5.63313	15.35473	-0.48360
H	6.96678	12.32834	1.92731

54

TS_F-F' G(ha): -3784.850256

W	2.41727	12.99399	5.05744
W	2.48471	15.51464	2.85304
W	2.38981	12.37481	1.74596
W	5.23099	14.78404	4.58362
W	5.06543	11.60384	3.57945
Zr	5.20982	14.19826	1.23387
O	1.43589	12.55519	6.40459
O	1.45394	16.88848	2.70778
O	1.27840	11.45262	0.80297
O	6.20115	15.63245	5.73074
O	5.97714	10.18258	3.91211
O	1.65841	14.61352	4.42083
O	1.61718	12.12470	3.55443
O	3.83871	14.02866	5.79080
O	3.74250	11.55042	4.96320
O	3.70618	15.72287	1.46699
O	1.57364	14.13230	1.83080
O	3.83769	16.06661	4.12040
O	3.62365	12.99547	0.50862
O	3.69622	11.04243	2.35400
O	6.06212	15.10766	2.96484
O	5.95751	12.97432	4.61642
O	5.99863	12.32155	2.02655
O	3.85223	13.58711	3.11510
Zr	8.62383	12.68442	-0.69356
O	7.43300	13.99013	0.56460
O	10.69106	12.59448	-0.22128
W	11.80028	11.87399	-1.52907
O	13.37792	11.95789	-0.83108
O	12.21393	11.05622	-3.28098
W	10.78372	10.71948	-4.51242
O	11.55480	10.01663	-5.88804
O	10.80319	12.60514	-4.86504

W	10.01167	13.84079	-3.54210
O	10.28233	15.35791	-4.32144
O	11.65267	13.52607	-2.54382
O	11.17347	10.09459	-1.05374
W	9.41443	9.54677	-1.67651
O	9.25440	7.92856	-1.09490
O	10.33219	9.22641	-3.40007
O	8.92248	10.77648	-4.98756
W	7.62615	11.50990	-3.69663
O	6.15357	11.33119	-4.57986
O	8.31094	13.24159	-4.26645
O	9.16169	14.29729	-1.94766
O	8.63366	10.58374	-0.34714
O	7.83456	9.80655	-2.78550
O	7.09570	12.28400	-2.08430
O	9.68129	11.72330	-2.55395
O	8.16316	13.02527	1.42772
O	4.37239	15.99189	-1.08737
H	3.87638	16.18882	-0.26126
O	5.48309	15.21912	-0.54705
H	7.16799	12.49613	1.85220
54			
F'	G(ha): -3784.847513		
W	2.48354	12.98994	5.12556
W	2.47395	15.46757	2.86892
W	2.38753	12.30100	1.83258
W	5.25840	14.80943	4.57431
W	5.12787	11.61899	3.59845
Zr	5.17990	14.14627	1.24465
O	1.52587	12.57597	6.49938
O	1.42787	16.82958	2.70558
O	1.26945	11.34880	0.92573
O	6.23750	15.69813	5.68456
O	6.04439	10.20571	3.96438
O	1.69207	14.60011	4.46085
O	1.64133	12.09945	3.65434
O	3.88782	14.08295	5.81628
O	3.80858	11.58313	5.03155
O	3.67269	15.65587	1.44903
O	1.56356	14.05875	1.88063
O	3.85119	16.07147	4.08772
O	3.60447	12.89797	0.56648
O	3.70826	10.99523	2.45198
O	6.06721	15.09728	2.93371
O	5.99821	13.01282	4.64017
O	6.00108	12.30957	2.06082
O	3.86273	13.57089	3.14839
Zr	8.62271	12.73057	-0.77011
O	7.42917	14.00962	0.52503
O	10.65169	12.53811	-0.21561

W	11.77863	11.77192	-1.48561
O	13.32808	11.77142	-0.72421
O	12.21672	10.94917	-3.22444
W	10.82230	10.69155	-4.51773
O	11.61337	9.96023	-5.86564
O	10.95357	12.57829	-4.84921
W	10.17330	13.84129	-3.55157
O	10.55176	15.35149	-4.29641
O	11.75002	13.44033	-2.48541
O	11.03826	10.02632	-1.04805
W	9.28272	9.57366	-1.75084
O	9.01330	7.96610	-1.18421
O	10.25408	9.21622	-3.43311
O	8.99077	10.84775	-5.06262
W	7.67819	11.64289	-3.81971
O	6.23659	11.54496	-4.76308
O	8.47409	13.33617	-4.35142
O	9.26919	14.32142	-1.99046
O	8.49990	10.65136	-0.44349
O	7.76708	9.92218	-2.91740
O	7.12596	12.42585	-2.22178
O	9.69387	11.73984	-2.59215
O	8.21562	13.12840	1.41848
O	4.27667	15.82594	-1.13397
H	3.80282	16.04721	-0.30037
O	5.39795	15.06274	-0.60040
H	7.42387	12.59362	1.82965
54			
TS_F'-G		G(ha): -3784.825364	
W	1.96022	13.19024	5.32653
W	2.80286	15.41678	2.96187
W	1.63821	12.42604	2.07348
W	5.18489	13.94980	4.77894
W	4.01782	10.96014	3.89595
Zr	4.88534	13.17762	1.49211
O	0.91289	13.18936	6.69946
O	2.29011	17.04763	2.71390
O	0.27096	11.86516	1.17972
O	6.41040	14.51135	5.85869
O	4.39541	9.32978	4.32243
O	1.76993	14.94591	4.58084
O	0.85474	12.58224	3.87986
O	3.64642	13.79170	6.01228
O	2.72768	11.42879	5.31177
O	3.99066	15.09873	1.56906
O	1.46052	14.36123	2.02811
O	4.30520	15.58116	4.18677
O	2.99251	12.54413	0.79997
O	2.44728	10.79523	2.76085
O	6.05097	13.82892	3.13741

O	5.27279	12.01385	4.94397
O	5.05646	11.28421	2.38242
O	3.45139	13.18828	3.37314
Zr	7.19949	11.80692	-1.91490
O	5.81059	12.45501	-0.39802
O	8.56249	12.85176	-0.63111
W	10.31764	12.99701	-1.25955
O	11.12443	13.88469	-0.02055
O	11.78805	12.70848	-2.52674
W	11.45871	11.75731	-4.16786
O	12.98579	11.73669	-4.96895
O	10.60723	13.35244	-4.77855
W	8.78749	13.79319	-4.12125
O	8.49715	15.26739	-4.96877
O	9.72080	14.35970	-2.51107
O	10.59519	11.13168	-0.77637
W	9.90397	9.76131	-1.96341
O	10.39718	8.28434	-1.22424
O	11.46396	10.16697	-3.07251
O	10.29116	10.79726	-5.33189
W	8.38641	10.55822	-4.82946
O	7.80892	9.66889	-6.19166
O	8.21454	12.42211	-5.36863
O	7.23025	13.53231	-3.12839
O	8.18073	10.05988	-1.24443
O	9.07333	9.19165	-3.62309
O	6.89795	10.75267	-3.75207
O	9.26862	11.77811	-2.99668
O	6.33040	14.11540	0.16029
O	5.41431	10.68588	-1.24341
H	7.28184	13.85600	0.15284
O	5.68949	9.48131	-0.53276
H	6.66806	9.38998	-0.65740
54			
G	G(ha): -3784.861027		
W	1.88095	13.17805	4.41464
W	2.91305	15.66914	2.41981
W	2.40691	12.63232	1.14233
W	5.00258	14.38870	4.67569
W	4.50406	11.35335	3.39127
Zr	5.58082	13.85630	1.36968
O	0.56432	12.93448	5.50282
O	2.24826	17.23702	2.13185
O	1.37167	11.97857	-0.07620
O	5.85483	15.01134	6.04399
O	5.00635	9.75713	3.81527
O	6.22636	14.19696	-0.75295
O	1.61755	14.95034	3.72784
O	1.22584	12.55589	2.72173
O	3.26352	13.92988	5.50327

O	2.87735	11.53846	4.49216
O	4.43349	15.62051	1.33507
O	1.98446	14.53491	1.14523
O	4.04908	15.93527	3.97366
O	3.98597	13.00774	0.24531
O	3.25655	11.06875	1.92779
O	6.21463	14.52877	3.28508
O	5.33143	12.47105	4.74861
O	5.78883	11.92015	2.15971
O	3.76733	13.52522	2.85940
Zr	8.05864	13.01506	-1.19829
O	8.41783	14.83611	0.69449
O	10.12801	13.18997	-0.70075
W	11.35247	12.39776	-1.83526
O	12.88547	12.73638	-1.11255
O	11.91729	11.37392	-3.43324
W	10.58343	10.72156	-4.64208
O	11.47944	9.90397	-5.87130
O	10.43839	12.52909	-5.27060
W	9.47795	13.85595	-4.16473
O	9.63333	15.26945	-5.14605
O	11.10003	13.86092	-3.09525
O	10.86963	10.65238	-1.11601
W	9.19945	9.85667	-1.70709
O	9.15689	8.33848	-0.88522
O	10.22161	9.37160	-3.32884
O	8.75048	10.52374	-5.17668
W	7.33078	11.30680	-4.05098
O	5.92414	10.85654	-4.94726
O	7.87386	12.99798	-4.85101
O	8.51893	14.44804	-2.67801
O	8.25587	11.00632	-0.58264
O	7.65848	9.78093	-2.89024
O	6.65519	12.23610	-2.59170
O	9.29798	11.89204	-2.88704
H	5.53140	14.03186	-1.40578
O	7.87704	13.52607	1.01773
O	7.34970	15.80451	0.84838
H	6.93551	15.76253	-0.04808

57

TS_E-G Energy: -3861.230715

W	1.91402	13.19879	4.44748
W	2.69721	15.57529	2.25858
W	2.44545	12.42557	1.21017
W	4.96916	14.61955	4.49313
W	4.61584	11.45259	3.52486
Zr	5.59556	13.80027	1.23768
O	0.62308	12.99921	5.57203
O	2.00598	17.08611	1.80799
O	1.41821	11.62172	0.08040

O	5.79834	15.41043	5.78368
O	5.19483	9.93118	4.09083
O	6.13554	14.05204	-0.87186
O	1.51277	14.94563	3.60014
O	1.26419	12.46722	2.81204
O	3.25127	14.17623	5.39300
O	2.97792	11.65204	4.65606
O	4.28325	15.52104	1.06188
O	1.92079	14.31021	1.04395
O	3.92843	16.05365	3.66364
O	3.98237	12.77974	0.25550
O	3.33779	10.97887	2.13969
O	6.15929	14.68863	3.08612
O	5.37990	12.73481	4.76004
O	5.83279	11.94488	2.21898
O	3.74468	13.54482	2.81035
Zr	8.14272	12.99847	-1.18696
O	8.33563	14.59624	0.39150
O	10.22999	12.99399	-0.66976
W	11.43225	12.20106	-1.81358
O	12.96839	12.44080	-1.05265
O	11.98544	11.20663	-3.43680
W	10.64155	10.65171	-4.68238
O	11.52471	9.83197	-5.92313
O	10.59193	12.48526	-5.25674
W	9.67121	13.82087	-4.12488
O	9.91843	15.24999	-5.06840
O	11.27138	13.71907	-3.02625
O	10.86284	10.45342	-1.16641
W	9.15491	9.77858	-1.79885
O	9.02470	8.23350	-1.03288
O	10.19271	9.28255	-3.42631
O	8.81401	10.55811	-5.26400
W	7.41264	11.37521	-4.13842
O	6.00454	11.01432	-5.07736
O	8.04379	13.06388	-4.87864
O	8.70816	14.42781	-2.65130
O	8.26306	10.92737	-0.64940
O	7.64331	9.79752	-3.01941
O	6.75260	12.28150	-2.66972
O	9.38079	11.83092	-2.92421
H	5.50126	13.69436	-1.50911
O	7.77340	13.40006	0.98236
O	7.04430	16.01890	0.48700
H	6.76377	15.67728	-0.39426
O	5.78073	17.32464	0.50823
H	5.73428	17.57476	-0.42971
H	4.86911	16.42356	0.79841

54

TS_G1 G(ha): -3784.847997

W	1.54228	14.43478	4.24003
W	3.12738	15.33824	1.41764
W	2.13383	12.17936	1.82535
W	4.81807	15.16669	4.28048
W	3.82553	12.00946	4.68601
Zr	5.45857	12.89222	1.83072
O	0.14714	14.98353	5.09744
O	2.76644	16.59022	0.28361
O	1.05861	11.12266	0.98183
O	5.70942	16.29542	5.23783
O	3.98282	10.83602	5.94463
O	6.36447	12.60867	-0.14503
O	1.65239	15.58736	2.71368
O	0.87257	13.10306	3.03269
O	2.99115	15.45869	4.97147
O	2.20873	12.97598	5.29000
O	4.65868	14.54175	0.73341
O	2.07358	13.82452	0.79218
O	4.21090	16.22600	2.76446
O	3.80404	11.79423	1.10097
O	2.62008	11.16766	3.41570
O	6.10578	14.38815	3.18626
O	4.78579	13.55919	5.37309
O	5.25076	11.64398	3.55268
O	3.54073	13.64784	3.01324
Zr	8.07063	11.75569	-1.19598
O	8.03853	10.68313	0.96758
O	10.07154	11.99287	-0.54476
W	11.39202	12.10429	-1.85356
O	12.85594	12.27760	-0.95313
O	12.08761	12.13677	-3.70475
W	10.85993	11.97794	-5.16871
O	11.85017	12.05516	-6.58087
O	10.38779	13.80246	-4.81225
W	9.23678	14.21283	-3.26114
O	9.11835	15.93176	-3.38369
O	10.85880	13.93545	-2.21847
O	11.25424	10.18474	-2.13959
W	9.73855	9.52893	-3.16776
O	9.98685	7.81994	-3.22665
O	10.78208	10.10980	-4.74128
O	9.07473	11.77483	-5.83899
W	7.57231	11.63846	-4.56147
O	6.23705	11.47045	-5.64396
O	7.78966	13.56344	-4.38812
O	8.23059	13.79395	-1.75933
O	8.64968	9.77603	-1.68448
O	8.19210	9.81309	-4.31408
O	6.79119	11.58883	-2.87215
O	9.43807	11.86617	-3.14274

H	5.93835	13.25860	-0.72476
O	7.05572	9.99639	0.53249
O	7.53878	12.13981	2.09894
H	7.60229	11.62853	2.92198

52

rad_G G(ha): -3709.101804

W	1.91996	13.44204	4.72181
W	2.65687	15.43535	2.12026
W	2.23705	12.16423	1.63218
W	4.97595	14.80007	4.42492
W	4.56309	11.52655	3.93273
Zr	5.33148	13.51353	1.31599
O	0.70066	13.41119	5.94214
O	1.88263	16.86260	1.53420
O	1.15865	11.20112	0.68917
O	5.89527	15.75968	5.52706
O	5.17850	10.09572	4.67540
O	6.05061	13.65708	-0.71558
O	1.50968	14.98840	3.66615
O	1.18179	12.41269	3.27928
O	3.33868	14.48305	5.48323
O	3.01386	11.90827	5.09179
O	4.09523	15.18981	0.96335
O	1.70708	13.99430	1.23053
O	3.89225	16.09841	3.46978
O	3.72466	12.39192	0.53373
O	3.23160	10.86355	2.67797
O	6.07449	14.65105	2.92923
O	5.42537	12.96717	4.90863
O	5.72188	11.82158	2.49633
O	3.66709	13.48080	2.97326
Zr	8.10287	12.97390	-1.29402
O	8.21358	14.43860	0.49615
O	10.10303	12.78525	-0.55358
W	11.35617	12.03452	-1.68054
O	12.81971	12.06126	-0.76052
O	11.99343	11.18913	-3.35065
W	10.75757	10.89800	-4.78532
O	11.70417	10.15860	-6.02648
O	10.89226	12.78027	-5.12132
W	9.94064	14.04699	-3.93174
O	10.37611	15.55337	-4.65772
O	11.40815	13.68892	-2.70402
O	10.58971	10.27947	-1.30392
W	8.92645	9.80734	-2.18240
O	8.61493	8.20171	-1.62663
O	10.08304	9.43540	-3.74665
O	8.99614	11.01533	-5.53437
W	7.53709	11.80238	-4.46187
O	6.21410	11.66680	-5.56598

O	8.36786	13.50594	-4.92845
O	8.86324	14.54365	-2.49134
O	7.98317	10.88454	-0.98725
O	7.54533	10.10031	-3.52288
O	6.80068	12.60656	-2.96936
O	9.39113	11.96363	-2.99820
H	5.43804	13.49029	-1.44681
O	7.79720	13.26365	0.97901
53			
H	G(ha): -3709.730420		
W	1.78580	13.46423	4.56797
W	2.71226	15.56709	2.12009
W	2.16778	12.35512	1.42009
W	4.90800	14.69552	4.46110
W	4.36792	11.47780	3.75782
Zr	5.34768	13.57938	1.28439
O	0.52143	13.41777	5.74385
O	2.01852	17.05970	1.59386
O	1.07761	11.50205	0.38589
O	5.82196	15.54435	5.65728
O	4.89305	9.97895	4.43687
O	5.97796	13.79892	-0.77735
O	1.48248	15.08737	3.59830
O	1.05733	12.55587	3.04508
O	3.21986	14.38858	5.44358
O	2.79706	11.86045	4.88637
O	4.17728	15.33009	1.00274
O	1.72825	14.22727	1.11395
O	3.91107	16.09858	3.55611
O	3.69777	12.56493	0.39254
O	3.05244	10.94665	2.42613
O	6.05071	14.60657	3.00153
O	5.26239	12.81914	4.84559
O	5.58467	11.79521	2.38057
O	3.59585	13.52496	2.89218
Zr	7.94161	12.88421	-1.19464
O	8.15389	14.49998	0.60140
O	9.98958	13.03873	-0.47398
W	11.32245	12.37526	-1.57365
O	12.77755	12.67512	-0.69081
O	12.05690	11.51612	-3.19494
W	10.85710	10.94597	-4.57762
O	11.88416	10.25900	-5.78481
O	10.72111	12.79985	-5.05117
W	9.62292	14.00372	-3.92963
O	9.83694	15.49975	-4.76821
O	11.13642	13.94521	-2.71235
O	10.82081	10.56569	-1.06011
W	9.22634	9.80365	-1.87058
O	9.15205	8.21619	-1.19328

O 10.41355 9.48216 -3.42590
O 9.08915 10.76225 -5.29820
W 7.54796 11.41538 -4.25192
O 6.24684 11.01617 -5.31701
O 8.11830 13.17768 -4.84692
O 8.50464 14.44708 -2.50755
O 8.15766 10.82632 -0.74059
O 7.80967 9.79750 -3.20052
O 6.71169 12.19256 -2.79171
O 9.38192 11.93526 -2.84759
H 9.11174 14.33216 0.73323
H 5.32403 13.67248 -1.47774
O 7.60687 13.19218 1.01421
3
H2O G(ha): -76.421297
O 0.05104 0.05104 0.00000
H 1.01052 -0.06156 0.00000
H -0.06156 1.01052 0.00000
4
H2O2 G(ha): -151.542662
H -1.01286 -1.09772 -0.54675
O -0.24822 -1.18274 0.04675
O 0.24822 0.18274 0.04675
H 1.01286 0.09771 -0.54675
2
O2 G(ha): -150.332893
O -0.20767 -1.07033 0.04739
O 0.20767 0.07033 0.04739
2
singlet-O2 G(ha): -150.269961
O -0.20789 -1.07094 0.04739
O 0.20789 0.07094 0.04739
2
rad_OH G(ha): -75.739899
O -1.45681 -3.58682 1.34078
H -0.48564 -3.50521 1.23821
3
rad_OOH G(ha): -150.914682
H -1.01406 -1.10448 -0.54690
O -0.23111 -1.11972 0.04330
O 0.23298 0.12662 0.05090
